# Spatial Biodiversity Patterns of Madagascar's Amphibians and Reptiles

**DOI:** 10.1371/journal.pone.0144076

**Published:** 2016-01-06

**Authors:** Jason L. Brown, Neftali Sillero, Frank Glaw, Parfait Bora, David R. Vieites, Miguel Vences

**Affiliations:** 1 Department of Zoology, Southern Illinois University, Carbondale, Illinois, United States of America; 2 Centro de Investigação em Ciências Geo-Espaciais, Alameda do Monte da Virgem, Vila Nova de Gaia, Portugal; 3 Zoologische Staatssammlung, München, Germany; 4 Département de Biologie Animale, Université d’Antananarivo, BP 906, Antananarivo, Madagascar; 5 Museo Nacional de Ciencias Naturales, MNCN–CSIC, C/José Gutierrez Abascal 2, Madrid, Spain; 6 Zoological Institute, Technische Universität Braunschweig, Braunschweig, Germany; Trier University, GERMANY

## Abstract

Madagascar has become a model region for testing hypotheses of species diversification and biogeography, and many studies have focused on its diverse and highly endemic herpetofauna. Here we combine species distribution models of a near-complete set of species of reptiles and amphibians known from the island with body size data and a tabulation of herpetofaunal communities from field surveys, compiled up to 2008. Though taxonomic revisions and novel distributional records arose since compilation, we are confident that the data are appropriate for inferring and comparing biogeographic patterns among these groups of organisms. We observed species richness of both amphibians and reptiles was highest in the humid rainforest biome of eastern Madagascar, but reptiles also show areas of high richness in the dry and subarid western biomes. In several amphibian subclades, especially within the Mantellidae, species richness peaks in the central eastern geographic regions while in reptiles different subclades differ distinctly in their richness centers. A high proportion of clades and subclades of both amphibians and reptiles have a peak of local endemism in the topographically and bioclimatically diverse northern geographic regions. This northern area is roughly delimited by a diagonal spanning from 15.5°S on the east coast to ca. 15.0°S on the west coast. Amphibian diversity is highest at altitudes between 800–1200 m above sea-level whereas reptiles have their highest richness at low elevations, probably reflecting the comparatively large number of species specialized to the extended low-elevation areas in the dry and subarid biomes. We found that the range sizes of both amphibians and reptiles strongly correlated with body size, and differences between the two groups are explained by the larger body sizes of reptiles. However, snakes have larger range sizes than lizards which cannot be readily explained by their larger body sizes alone. Range filling, i.e., the amount of suitable habitat occupied by a species, is less expressed in amphibians than in reptiles, possibly reflecting their lower dispersal capacity. Taxonomic composition of communities assessed by field surveys is largely explained by bioclimatic regions, with communities from the dry and especially subarid biomes distinctly differing from humid and subhumid biomes.

## Introduction

Madagascar has long been renowned for its unique and diverse fauna and flora [1] and high proportion of microendemism, that is, range-restricted species characterized by exceedingly small distribution areas [2]. The island has long attracted the interest of biogeographers studying not only in the origins of Madagascar's biota, but also within-island distributional patterns and diversification mechanisms [2–8]. Current evidence suggests that the majority of Madagascar's vertebrate clades, but probably also most other animals and plants, colonized Madagascar over the Cenozoic and in many cases by overseas dispersal [9–12], after a major biotic change at the K/T boundary [13, 14]. A variety of factors (*i*.*e*. river barriers and montane refugia), have subsequently influenced speciation and community assembly within Madagascar [2, 6, 15–24], and only a combination of factors can explains the complex patterns observed [25].

In addition to plants [4] and lemurs [26], the herpetofauna have historically been one of the main biogeographic model groups in Madagascar. Explicit zoogeographic regions for the island were first proposed on the basis of reptile distribution patterns [3], and further discussions and analyses of both reptile [7, 27] and amphibian patterns [28] were published later on. Various pioneering biogeographic studies were entirely or partly based on herpetofaunal data. Some of these aimed to understand cladistic biogeographical relationships among sites in Madagascar [29, 30], defined null models of biodiversity patterns [16, 31], or modeled species’ distributions for species discovery and delimitation [32, 33]. Other herpetofauna-centered papers analyzed the impact of climate change on altitudinal distribution of montane faunas [34] and comprehensively assessed spatial and taxonomic conservation priorities in Madagascar [35–37]. Many of these works were made possible by an immense and intensified effort in inventorying these animals since the early 1990s, involving numerous survey studies across the island [38], routine application of bioacoustic and molecular methods [39], and inclusion of undescribed candidate species in many of the assessments [40]. Hence, although it is clear that many of Madagascar's amphibian and reptile species remain scientifically undescribed, the majority of them have been genetically characterized as candidate species [41, 42] and included in field guides [43], and thus are provisionally accessible for research and conservation. The paradoxical consequence is that Madagascar hosts one of the best studied and most scientifically accessible tropical herpetofauna, despite the large amount of undescribed species that have been revealed by these studies.

Notwithstanding this overall good state of knowledge, the study of classical biogeography patterns of Madagascar's herpetofauna remains patchy and elusive. Numerous studies provided information on species richness and weighted endemism, but were either based on rough distribution estimates [36, 37] or targeted only particular subgroups of amphibians and reptiles [22, 31, 44, 45]. Only recently, analyses of species richness, weighted endemism and turnover based on explicit distribution models became available for all amphibians, reptiles, and selected subgroups [25]. However, these have not yet been discussed from a taxon-specific perspective. Island-wide patterns of community composition of Madagascar's amphibians and reptiles have remained largely unstudied despite the availability of numerous surveys that followed roughly similar methodological approaches [38]. The relationship of range size and body size has not been comprehensively studied in Madagascar's amphibians and reptiles, although case studies in mantellid frogs suggest that body size might be an important factor influencing gene flow and diversification [20, 46].

Here, we provide a set of analyses aimed at partly filling these gaps in knowledge and providing a more complete baseline for future studies of biogeography, systematics, evolution and conservation of Madagascar's amphibians and reptiles. Our analyses include (i) calculation and comparison of species richness and endemism for various subgroups of the Malagasy herpetofauna, (ii) community turnover based on generalized dissimilarity modelling separately for amphibians and reptiles, (iii) range-body size relationships and range filling, and (iv) a comparison of the composition of real herpetofaunal communities across Madagascar as detected by survey work.

## Materials and Methods

### Terminology and taxonomy

The present analysis is based on distributional data of Malagasy amphibians and reptiles, from a compilation completed in 2008, and partly adjusted to account for subsequent taxonomic revisions. The compilation includes well-defined but scientifically undescribed confirmed candidate species [40] (i.e., species characterized by a substantial genetic divergence and by additional evidence for a status of independent evolutionary lineages). Given that the number of undescribed lineages keeps increasing [42] and their status is being modified in the course of taxonomic revisions, our data thus represent only a snapshot of taxonomic knowledge from 2008, with some updates; yet, our decision to include such candidate species leads to a more representative picture than the inclusion of nominal species only.

Furthermore, the majority of distributional information accumulated since 2008 has not been included in our dataset. As addressed again in the Discussion, the constantly changing taxonomy in some taxa, together with incomplete distribution range information and with the exclusion of some unrevised species at the time of compilation of our distributional dataset, might have led to biased representations of species richness and endemism in some subgroups (but does not invalidate general patterns reported herein).

We here use the term "reptiles" in its classical meaning, i.e., referring to all non-avian reptiles including squamates, chelonians and crocodylians. Given that only a limited number of turtles and one species of crocodile occur in Madagascar, our data mostly reflect the distributional patterns of squamates (lizards and snakes). The single crocodile species present in Madagascar (*Crocodylus niloticus*) was not included in our analysis.

**Fig 1:**
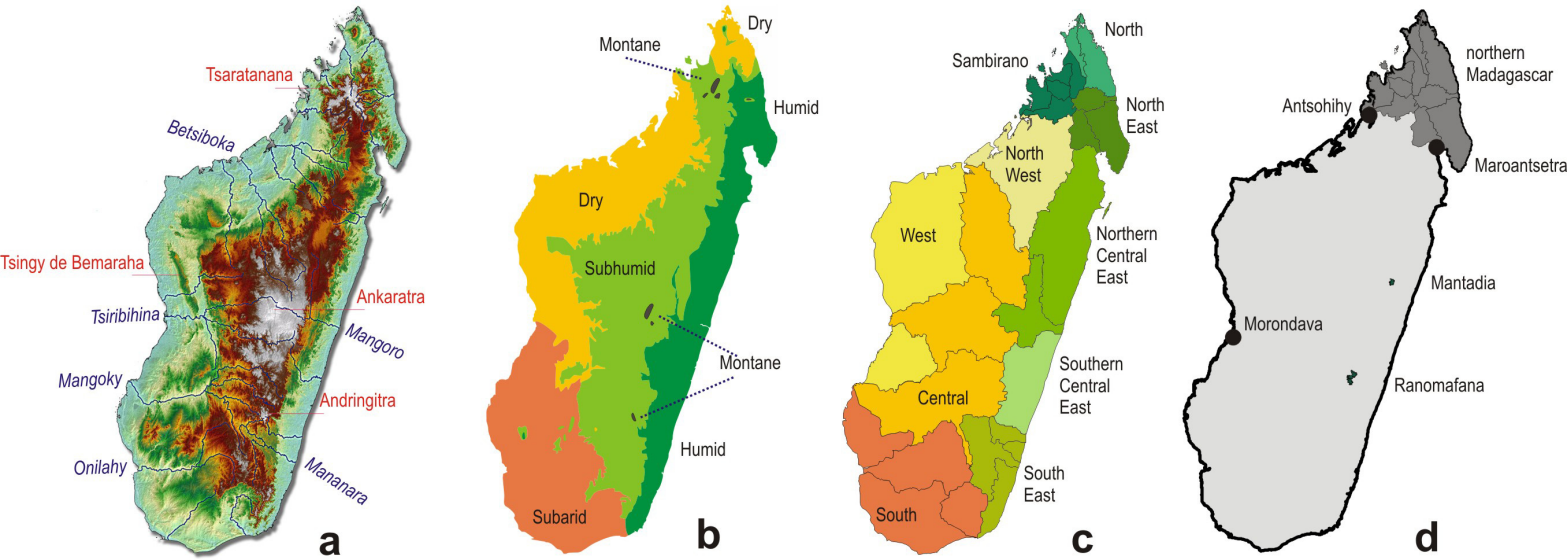
Maps of Madagascar. (a) topography with major mountain massifs and rivers, (b) major bioclimatic zones (herein called biomes) [47], (c) geographic regions ([48], boundaries based on watersheds ([2], and (d) delimitation of northern Madagascar as used herein including the Sambirano, North, and North East regions (map also shows a few towns and nature reserves discussed in the text).

Description of major biomes in Madagascar follows previous definitions of bioclimatic regions [47]. For convenience of naming particular geographic regions, we follow a previous approach [43, 48] that defined a series of regions with limits coinciding with those of major watersheds [2] (Fig 1C).

Northern Madagascar has been highlighted before as a center of endemism [48] and appeared as such also in our CWE (Corrected Weighted Endemism) analyses (see below). To understand differences in community composition we separated data from communities in humid and subhumid biomes (rainforest and montane forest) in this part of Madagascar (defined as the area north of a diagonal spanning from 15.5°S on the east coast to ca. 15.0°S on the west coast, i.e., roughly from Maroantsetra to Antsohihy; Fig 1D). Then, we compared the proportion of reptiles and amphibians among these two clusters of data points.

### Species Distribution Models

Because the distribution ranges of Madagascar's amphibians and reptiles have not been comprehensively mapped and distribution records are scattered, we used species distribution models (SDMs; also commonly called ecological niche models)[49] to obtain an estimate of the geographic ranges. The majority of our analyses are based on the SDMs compiled for a previous study [25]. These were calculated from 8362 occurrence records of 745 Malagasy amphibian and reptile species (325 and 420 species, respectively) and were limited to species that had, at minimum, 3 unique occurrence points at the spatial resolution (0.91 km^2^). For original occurrence records, see supplementary materials of Brown *et al*. [25].

Several species (n = 19) were excluded mostly because of convoluted taxonomy or controversial information on their distribution ranges, leaving a total of 727 species for final analysis (yielding a more inclusive dataset compared to the 679 species included previously; Brown *et al*.[25]). Species distribution models were generated in MaxEnt v3.3.3e [50] using parameters as described in Brown *et al*. [25] that accounted for sample selection biases [25, 51, 52]. The bias file up-weighted presence-only data points with fewer neighbors in the geographic landscape [53]. We used 19 standard variables characterizing current bioclimates for modeling (Worldclim 1.4; [54] as well as geology, aspect, elevation, solar radiation, and slope [55, 56]). All layers were projected to Africa Alber’s Equal-Area Cylindrical projection in ArcMap at a resolution of 0.91 km^2^. To limit over-prediction of SDMs we clipped each model as previously suggested [35]. Thus, we produced models representing suitable habitat within an area of known occurrence, based on a buffered minimum-convex-polygon of occurrence localities [25]. Continuous SDMs were converted to binary models using the ‘minimum training presence’ threshold.

### Species Richness, Corrected Weighted Endemism, and Generalized Dissimilarity Modeling

Species richness (SR) and corrected weighted endemism (CWE) were calculated from different taxonomic subsets of our estimate range maps (SDMs and buffered points). We used a hexagonal sampling grid at 5166km^2^, the same area used in previous studies [20, 25]. The hexagon is the most complex regular polygon and results in less orientation bias in analyses (vs. a square grid, as used in the aforementioned studies). CWE measures endemism by inversely weighting the proportion of endemics by their range size (species with smaller ranges are weighted more than those with large ranges; [57], and dividing this value by the local species richness [58]. CWE was calculated using SDMtoolbox v1 [52].

Generalized Dissimilarity Modeling (GDM; [59]) can be used to analyze and predict spatial patterns of turnover in community composition across large areas [25, 60]. To avoid computational limitations associated with pairwise comparisons of large datasets, we randomly sampled 2500 points throughout Madagascar from a ca. 10 km^2^ grid and then measured the absence or presence of each species at each locality [25]. The 23 environmental and geography layers used for SDMs were reduced to nine vectors in a principal component analyses and these were sampled at the same 2500 localities. Species communities as predicted by species occurrences at each of these sites, and environmental data, were then input into a generalized dissimilarity model using the R package: GDM R distribution package v1.1 (www.biomaps.net.au/gdm/GDM_R_Distribution_Pack_V1.1.zip). The GDM was then extrapolated based on the high resolution climate dataset [25, 59]. Classification of the GDMs was performed in SPSS v20 [61] using a two-step classification method that assesses AICc of a range of class numbers (here 2–30) to determine the optimum number of GDM classes; these were interpolated as described for the continuous model.

### Range Size and Body Size Relationships

For descriptive range-size statistics, distribution range-sizes were sampled for all species at 0.01 degrees^2^ from corrected binary SDMs (or buffered point data where applicable). We sampled for each modeled species two different range size measurements: corrected range size as in the SDMs clipped by buffered minimum-convex polygons (from [25]) and uncorrected range size based on SDM prediction without such clipping. We then calculated the ratio of the corrected range size divided by the uncorrected range size as a measure of range filling, that is, the proportion of the suitable habitat that is occupied by the species. We furthermore extracted the maximum, minimum and mean elevations predicted for each species from the adjusted SDMs and the buffered-point maps. Subsequently, we calculated the number of species estimated by the models to occur at different altitudes, at intervals of 100 m above sea level.

Body sizes of all species of Madagascar's amphibians and reptiles were compiled from the literature, mostly from a comprehensive field guide [43] and complemented with unpublished data and our own measurements where necessary (S1 Table). We used the maximum known male snout-vent length as the measurement of body size, as this variable was readily available for most species [43] and has previously been used in biogeographical and macroecological analysis [20, 62]. Although this variable ignores sexual dimorphism and different body shapes (of e.g. snakes and frogs), we are convinced it is an informative proxy in analyses over an entire and diverse herpetofauna, which in the case of Madagascar spans over three orders of magnitude with SVL values ranging from ca. 10 mm in *Stumpffia* frogs to ca. 2200 mm in *Acrantophis* snakes (larger crocodylians were not analyzed here).

We used Statistica 7.1 (Statsoft, Tulsa, USA) to calculate and visualize correlations between range size, altitudinal range, range filling, and body size, and for additional univariate tests (t-tests) comparing species numbers between regions or between taxa. We calculated univariate linear regressions and tested for differences between reptiles and amphibians, as well as between snakes and lizards, in analyses of variance (ANCOVA) defining body size as a covariable. Analyses of range filling were done using modeled species only; analyses of range size also included species known from only 1–2 sites.

### Herpetofaunal community analysis

From the plethora of herpetofaunal surveys published for Madagascar [38], we selected 20 surveys that were spatially representative of the most well surveyed areas at the time these data were compiled [8, 33, 63–81]. For this dataset we tabulated the amphibian and reptile species encountered in these inventories, and separated the species records for each site in case of multi-site inventories. Given the large number of new species described from Madagascar over the last years, ascertaining the taxonomic identity of species recorded during inventories over different decades is almost impossible, and any analysis uncritically using such unpublished species lists will inevitably be flawed. However, both the overall species numbers and the assignment of species to major clades (genera, subfamilies or families) can be considered as rather reliable, and we therefore based our analysis on such simplified taxon lists. We assigned all species to one of four major amphibian and eight reptile categories. Amphibians were (1) hyperoliid frogs, (2) microhylid frogs of the subfamily Cophylinae, (3) microhylid frogs of the subfamilies Scaphiophryninae and Dyscophinae, (4) mantellid frogs. Reptile categories were (1) turtles and tortoises, (2) typhlopid and xenotyphlopid snakes, (3) lamprophiid snakes, (4) iguanid lizards, (5) geckos, (6) skinks, (7) gerrhosaurids, and (8) chameleons. Some other major taxa with low number of species were not included in the analysis (i.e., boid snakes, ptychadenid and dicroglossid frogs) because they might have distorted the results due to the small sample size (1–3 species).

To better understand how well these ground-truthed communities identified in survey work differ from theoretical communities as calculated by SDM overlap and used in the GDM, we compiled the latter by extracting for each survey site the theoretical communities and comparing its composition (in species numbers of major taxonomic categories) with the observed communities.

## Results

### Species richness

**Fig 2:**
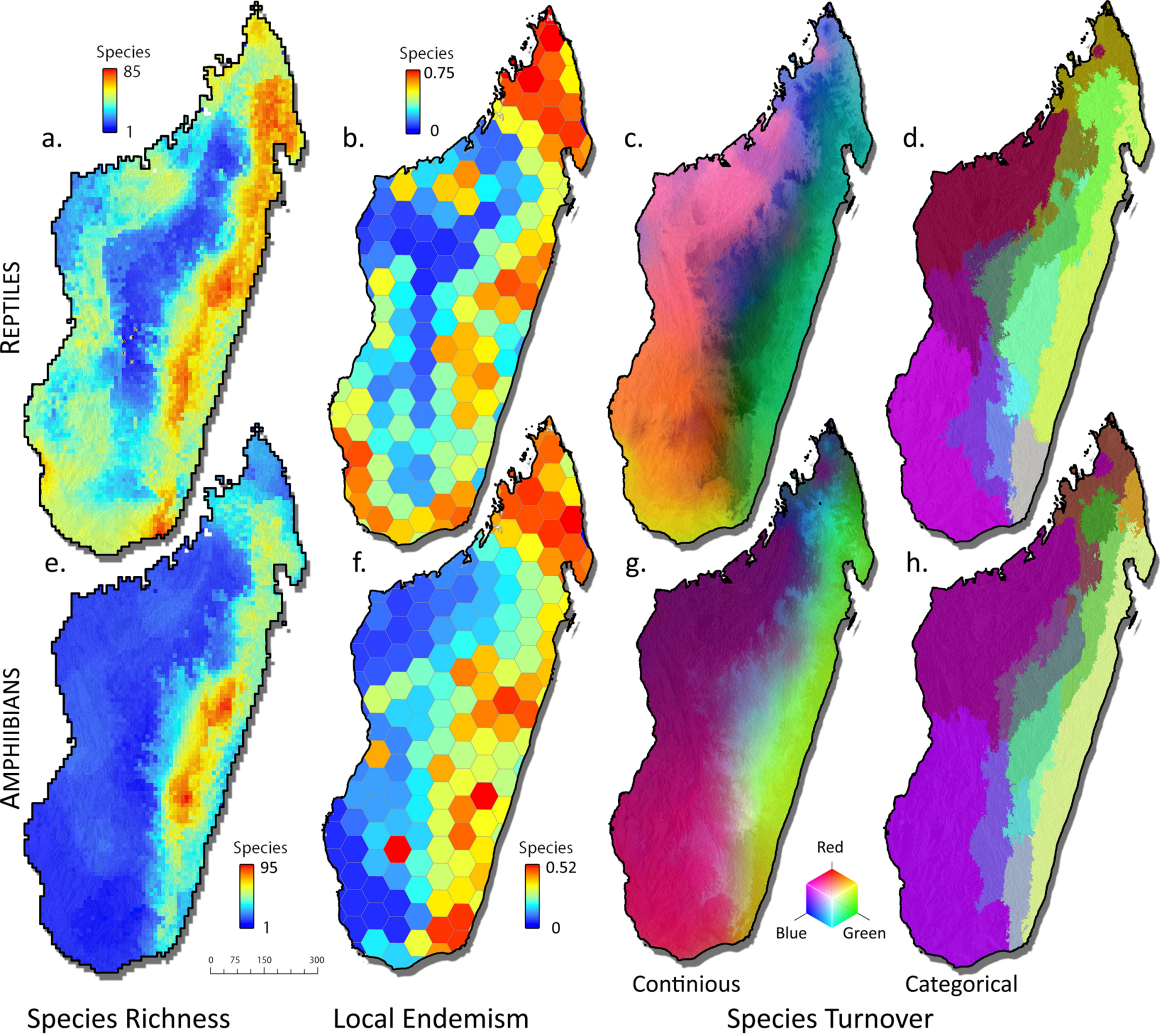
Biodiversity measures for reptiles and amphibians. Species richness (SR), endemicity (corrected weighted endemism, CWE), and turnover as measured by general dissimilarity models (GDM), based on the distribution of 325 species of amphibians and 420 species of reptiles from Madagascar. Species richness scales range from low (blue) to high (red) number of species per hexagon; Local endemism values range from low (blue) to high (red).

The humid rainforest biome of eastern Madagascar holds the highest concentration of species of both amphibians and reptiles, but differences are visible between the two groups (Fig 2). In reptiles, species richness (SR) is regularly distributed along the eastern rainforest band whereas the amphibian SR is concentrated in an area of the Northern Central East and Southern Central East regions. Whether the geographic gap between the two richness centers in the Northern and Southern Central East (corresponding to the two well-sampled regions around Ranomafana and Mantadia-Analamazaotra National Parks) reflects a real pattern or incomplete sampling remains uncertain with present data, although the modelling approach applied herein does account for sampling bias [25].

**Fig 3:**
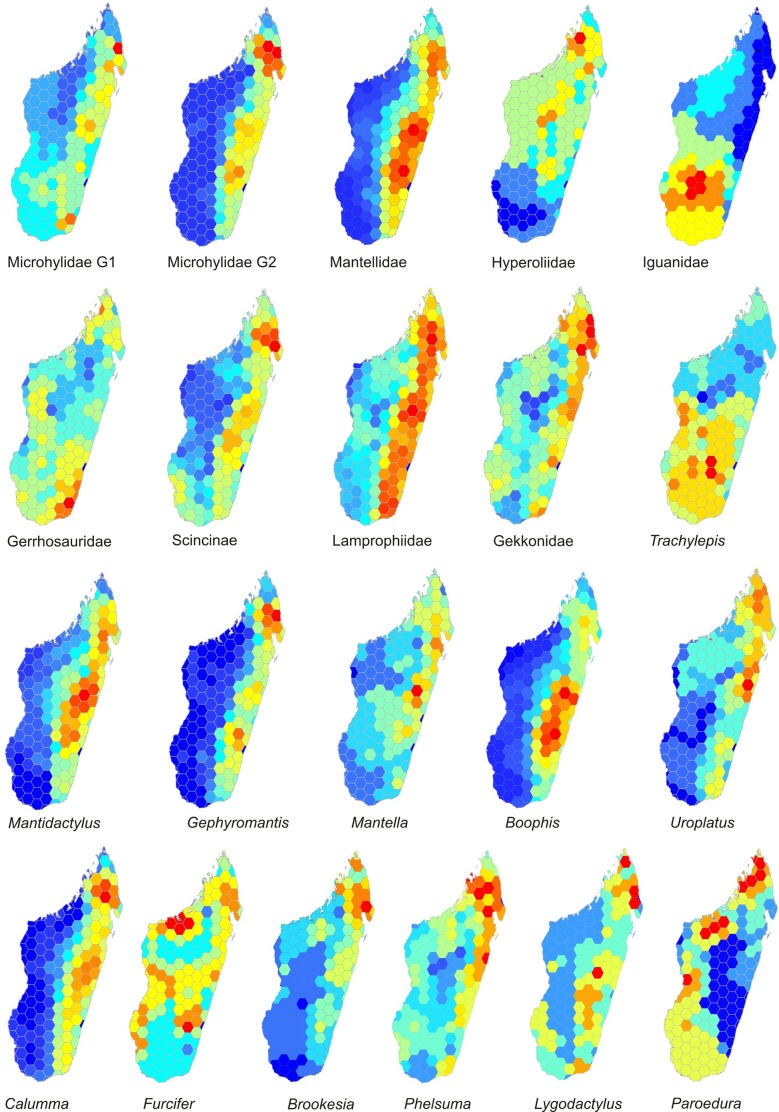
Species richness. Species richness (SR) calculated separately for different clades and subclades of Malagasy amphibians and reptiles. Microhylidae G1 includes scaphiophrynines whereas G2 includes cophylines.

A closer look at independent clades of amphibians (Fig 3) suggests that the high-central SR is mainly caused by the family Mantellidae whereas the microhylid subfamily Cophylinae has a more even pattern with high SR also in rainforests of the North East. Within the Mantellidae, a central concentration of SR is found in two independent subclades (especially in *Boophis* and to a lesser degree in *Mantidactylus*) but not in a third subclade (*Gephyromantis*).

Overall, reptile SR in Madagascar is highest in the humid biome and rather regularly distributed along its entire latitudinal extension (Fig 2). Comparatively, high SR values are also found along the west coast in the dry biome, and especially in the subarid biome in the South West. Species richness is lowest on the high plateau in the Central region, and in a poorly surveyed area southwest of Mahajanga in the West. Differences among reptile clades are stronger than among amphibian clades, with some clades and subclades lacking high SR in the humid biome. Most deviant are iguanas (Fig 3), which have no rainforest representative. This is also reflected in *Trachylepis* skinks and *Paroedura* geckos, each one with only two species colonizing the humid rainforest biome. In these three groups (iguanas, *Trachylepis*, *Paroedura*), SR peaks in the dry and especially subarid biomes of the South West and West, and additionally in northern Madagascar for *Paroedura* (Fig 3). Also chameleons of the genus *Furcifer* have the highest richness in the dry and subarid biomes, with only few species colonizing rainforest. The dwarf geckos of the genus *Lygodactylus* presented high SR on some central mountain massifs (Ankaratra, Ibity, Itremo, Andringitra), which are, as well, partly important centers of SR in *Trachylepis*, *Furcifer*, iguanids and gerrhosaurids, but with peaks not fully coinciding among these groups. Several reptile groups have SR centers located in a small northern portion of Madagascar (i.e., as defined here, the area north of a diagonal spanning from 15.5°S on the east coast to ca. 15.0°S on the west coast) but the precise limits of these areas of high SR do not always coincide spatially. In *Brookesia* ground chameleons, *Uroplatus* geckos, and skinks, similar to some amphibians (*Gephyromantis*, *Mantidactylus*, cophylines), the peak is in the rainforests of the North East. However, in *Paroedura* geckos and less distinctly in gerrhosaurids, SR peaks on the western coast of northern Madagascar (i.e., the Sambirano region).

### Corrected weighted endemism

Local endemism values, here measured and illustrated as Corrected Weighted Endemism (CWE), only partly coincide spatially with SR (Fig 2). Both in amphibians and reptiles, the largest extension of high-CWE cells is in the North East Madagascar. In amphibians and to a lesser degree in reptiles, CWE peaks are also observed in the Southern and Northern Central East, and in the South East. Slight differences compared to the 679 species dataset analyzed by Brown *et al*. [25] are recognizable in the CWE of reptiles, especially in the South East. Amphibians further have a single high-CWE cell coinciding with the Isalo Massif, and reptiles have an area of high CWE in the subarid South West, with the highest values coinciding with the Onilahy river mouth.

**Fig 4:**
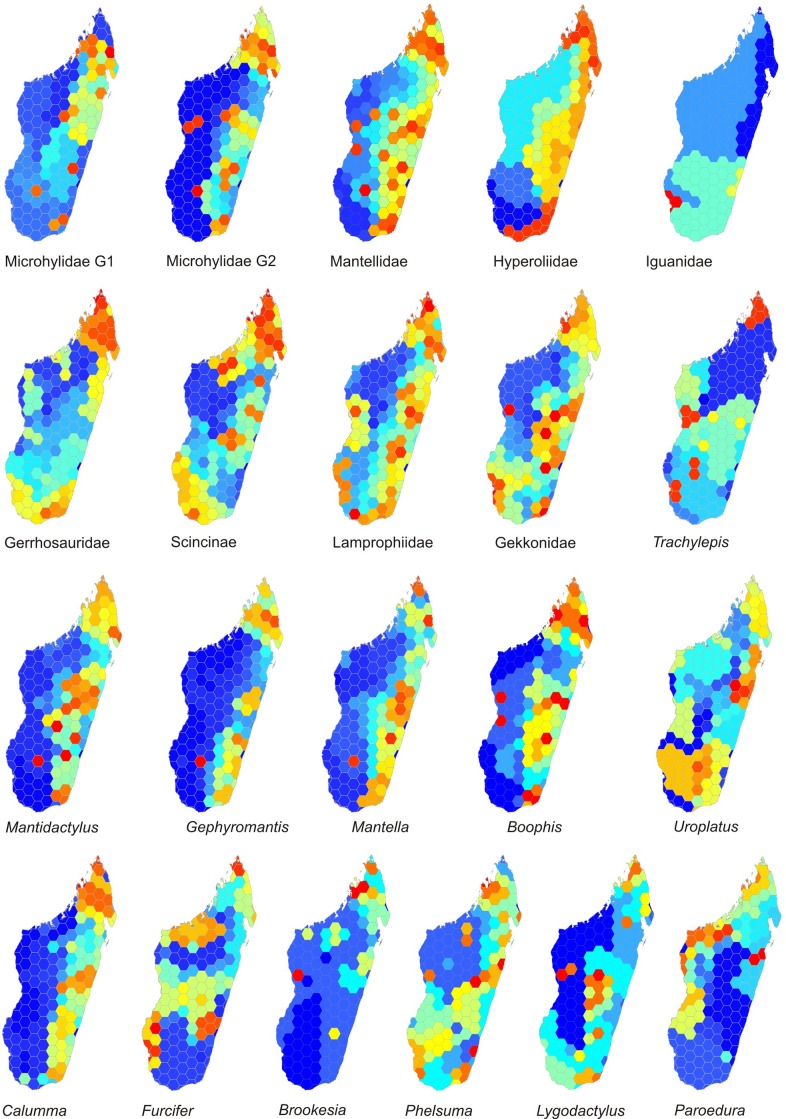
Endemism. Corrected weighted endemism (CWE) calculated separately for different clades and subclades of Malagasy amphibians and reptiles. Microhylidae G1 includes scaphiophrynines whereas G2 includes cophylines.

Comparing the major clades as well as the subclades (Fig 4) shows that high or very high CWE values in some or most grid cells in northern Madagascar are an almost general pattern, except in iguanas. Also leaf tail geckos (*Uroplatus*) do not show a particularly high CWE in the North (but see Discussion).

The coastal areas of the South West have high CWE, especially in iguanas, but also in geckos among the major clades, and in *Trachylepis* skinks, *Furcifer* chameleons, and to a lesser degree, in *Phelsuma* day geckos among the subclades. An area of high CWE cells in the North West is evident in three subclades: in skinks of the subfamily Scincinae, in *Furcifer* chameleons, and in geckos. Similarly, a cell coinciding with the Tsingy de Bemaraha limestone massif in western Madagascar has high CWE in *Boophis* treefrogs, as well as in *Lygodactylus* and *Phelsuma* geckos, and *Brookesia* ground chameleons. This is due to the presence of species at this site which had not been recorded elsewhere at the time our dataset was compiled, although for some of them (e.g., *B*. *tampoka*) new records have in the meantime become available and therefore, the high CWE at Bemaraha at least for *Boophis* will likely not be recovered in future studies based on updated datasets. Geckos, in general, and especially *Lygodactylus* dwarf geckos, as well as skinks, also have an area of high CWE coinciding with the Central Plateau of Madagascar around the Ankaratra-Itremo-Ibity massifs.

### Altitudinal distribution of species richness

We sampled altitudinal SR of amphibians and reptiles as the number of species predicted to occur in altitudinal sections of 100 m according to their modelled distribution. As discussed below, this approach almost certainly overestimates the number of species actually occurring at a certain elevation, but represents the most objective means to assess and compare altitudinal diversity across the entire herpetofauna with current data. We therefore do not report here absolute numbers, but general trends only.

**Fig 5:**
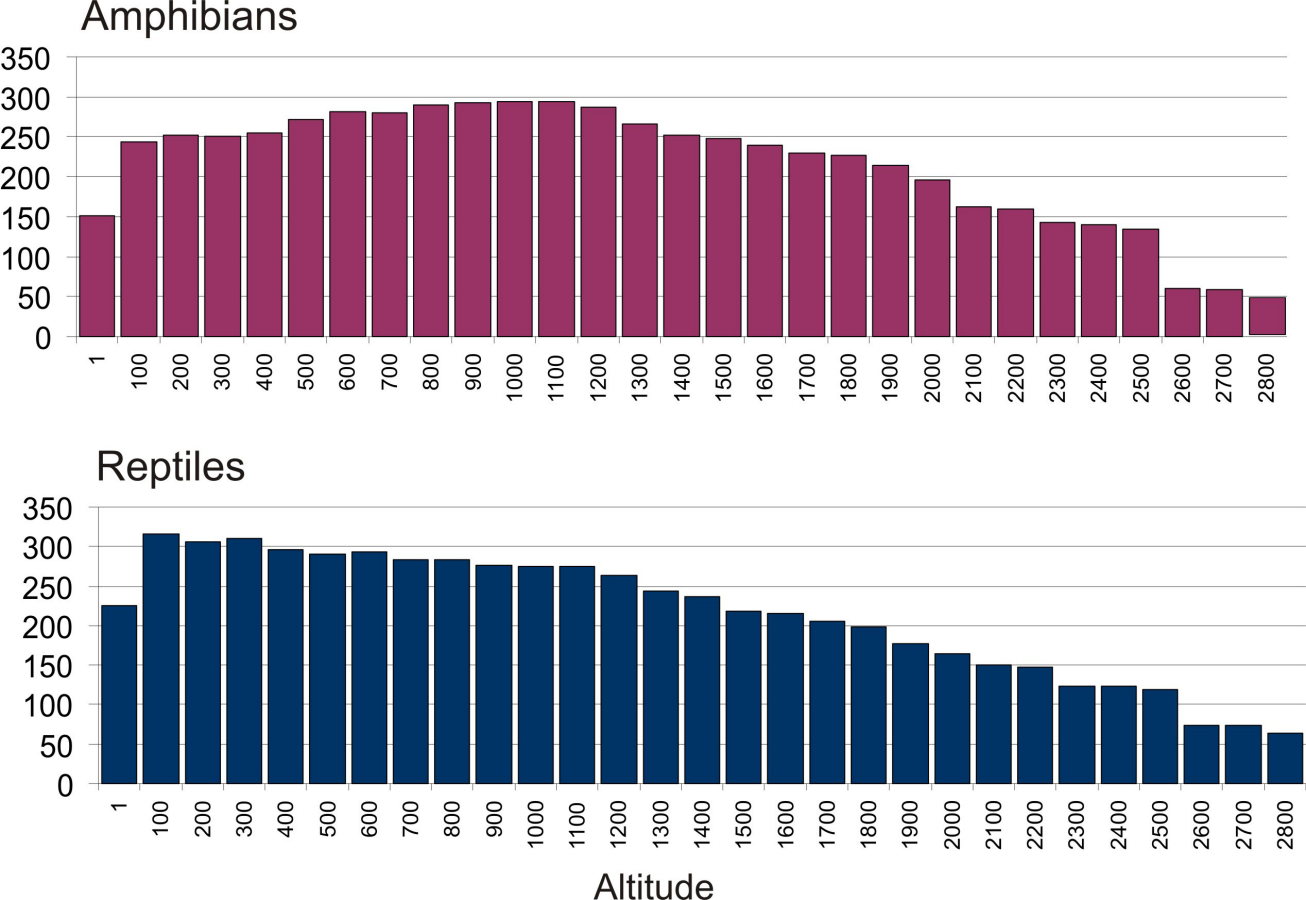
Species richness by elevation. Number of specimens of amphibians and reptiles, predicted by the adjusted SDMs to occur at certain elevations at intervals of 100 m above sea level. Presumably due to over-prediction the inferred elevational ranges probably are larger than the realized ones, giving higher numbers of species than actually occurring in lowlands and high elevations.

According to our analyses, the SR of amphibians continuously increases with increasing elevation, reaching a maximum between 800‒1200 m a.s.l., with the highest value at 1000 m a.s.l. (Fig 5). From 1000 m higher, SR is negatively correlated with elevation—with distinct drops of SR values from 2000 to 2100 m a.s.l. and from 2500 to 2600 m a.s.l. The elevational SR of reptiles shows a different trend, with a maximum SR value in the lowlands (100 m a.s.l.) and a continuous decrease of SR with increasing elevation. Again, two distinct drops of SR values are seen at high elevations, one from 2200 to 2300 m a.s.l. and one from from 2600 to 2700 m a.s.l. The differences seen between amphibians and reptiles are statistically significant, with both minimum and maximum elevation per species, and elevational range, being on average higher in amphibians (t-tests: P = 0.016, P <0.001, P < 0.001, respectively).

### Areas of Endemism based on Generalized Dissimilarity Modelling

Generalized Dissimilarity Modelling, as applied here, reconstructs for a set of sites across the landscape the theoretical communities of species based on the overlap of their distribution ranges, and then calculates pairwise differences between these communities. On this basis, it identifies changes in the communities which reflect high species turnover, and can be interpreted as boundaries of biogeographic regions. The GDMs reconstructed herein for amphibians and reptiles reflect large differences between the distributional patterns seen in the two groups. Given that amphibians are mostly distributed in the humid and subhumid biomes, with few species in dry and subarid biomes, the main GDM boundaries run in a north-south direction. In reptiles, a more complex subdivision especially of the subhumid/montane biomes is reconstructed (Fig 2).

Both in amphibians and reptiles, a trend is visible of more continuous community change in low elevations along the east coast, with no latitudinal boundary in the categorical GDM of amphibians and only one for reptiles (Fig 2). On the contrary, at higher elevations a higher number of latitudinal breaks exist that mainly are located in the subhumid/montane biomes and, thus, more distinct patterns of turnover are observed. Both amphibian and reptile GDMs reconstruct a major area of turnover (corresponding to the limit between dry and subarid biomes) in the area around Morondava in the West. Also, in both amphibians and reptiles, northern Madagascar stands out separately, although its boundaries are estimated more southwards than formally defined and as reflected by richness and endemism patterns. In amphibians, a region roughly corresponding to the Tsaratanana Massif stands out as separate area of endemism.

### Range size, range filling and body size

**Fig 6:**
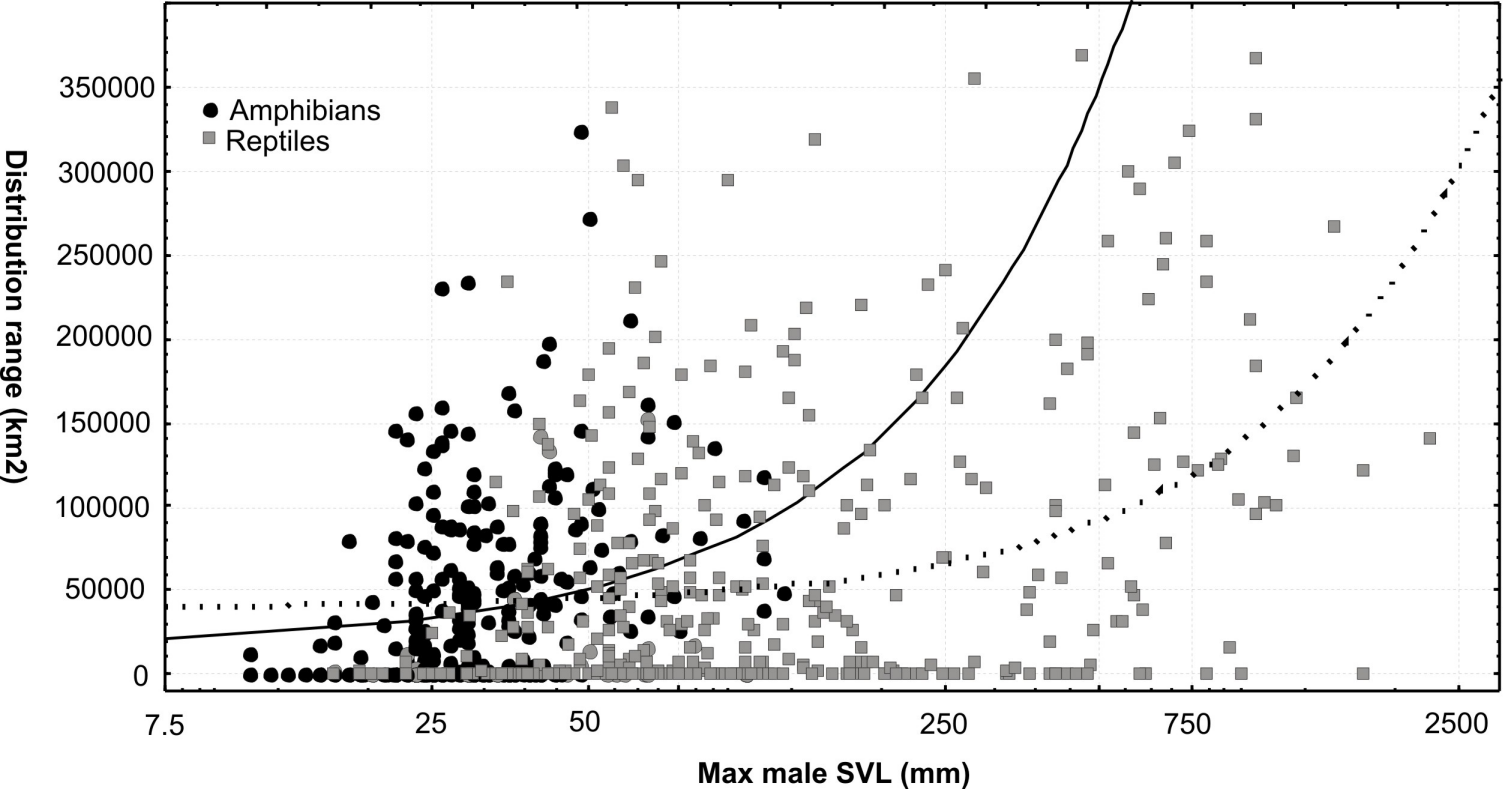
Body size and range sizes correlations. Correlation of body size with range size in amphibians (black circles and solid line) and reptiles (white squares and dashed line). Both correlations are highly significant (see text).

We found a clear and highly significant correlation of range size (spatial extent of the distribution area, in km^2^) with body size (Fig 6), measured as maximum male snout-vent length, in both amphibians (parametric correlation: r = 0.2231, P < 0.001; non parametric Spearman correlation: R = 0.3403, P < 0.001) and reptiles (r = 0.3538, P < 0.001; R = 0.3280, P < 0.001, respectively).

Range sizes of reptiles were larger than those of amphibians (range sizes, mean ± SD: 61113 ± 83100 km^2^ vs. 40326 ± 53719 km^2^; t-test: P = 0.001) as were their body sizes (SVL 202.5 ± 280.5 mm vs. 34.7 ± 17.9 mm; t-test: P < 0.001). The large standard deviations of range size values reflect the presence of a considerable number of species with distribution areas much larger than the average, combined with a high number of microendemic species. The larger ranges of reptiles compared to amphibians are probably caused mainly by their larger body sizes. Comparing the range size values of amphibians and reptiles by ANCOVA with SVL as co-variable revealed a highly significant influence of SVL (P < 0.001), but no significant influence of the taxonomic category (P = 0.612). Within reptiles, we furthermore tested whether the larger range sizes of snakes vs. lizards (mean 98785 ± 105015 km^2^ vs. 48703 ± 70307 km^2^; t-test: P < 0.001) were a true pattern suggestive of different barriers to dispersal, differential dispersal capacities among the two groups, or explainable by body size influences only. In this case, ANCOVA revealed a significant influence both of SVL (P < 0.001) and of taxonomic category (P < 0.001), suggesting that indeed, at similar body sizes, snakes in Madagascar appear to have larger range sizes than lizards.

The correlation between body size and range size is also extended to elevational ranges. In amphibians, altitudinal ranges and maximum elevation were positively correlated with SVL, while minimum elevation was negatively correlated with SVL. This suggests that larger species occur over wider elevational ranges, which probably can be explained with their larger spatial ranges (non-parametric Spearman correlations for minimum and maximum elevation, and elevational range: R = -0.215, 0.231, 0.228; P < 0.001 in all three analyses). Similar results were obtained for reptiles, albeit with slightly weaker correlation coefficients (R = -0.222, P < 0.001; R = 0.182, P < 0.001 and R = 0.142; P < 0.01).

**Fig 7:**
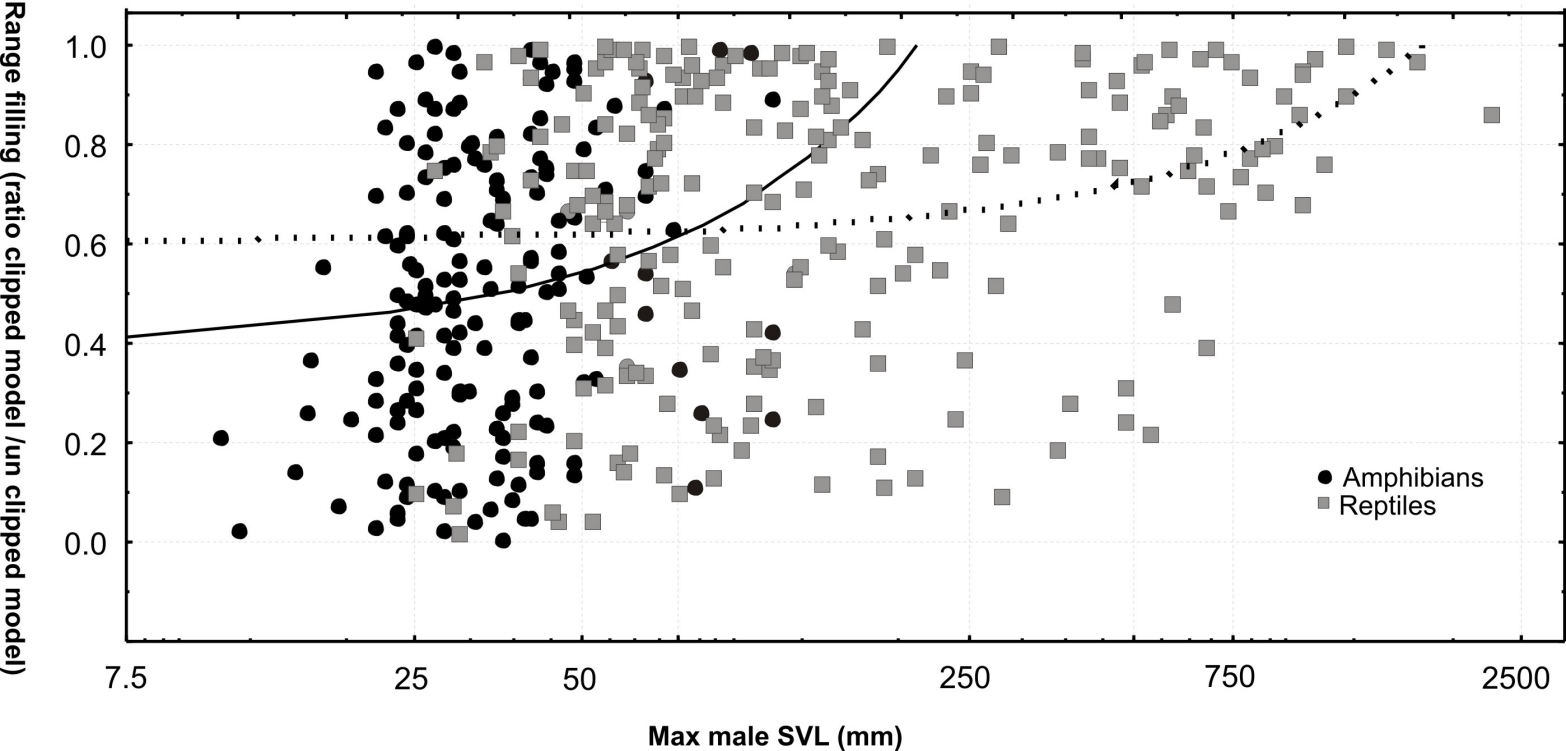
Range filling of reptiles and amphibians. Correlation of range filling (ratio of range sizes of clipped distribution model vs. full distribution model) with SVL, separately for amphibians (black solid circles) and reptiles (squares). Analyses carried out after removing all taxa with 1–2 data points only.

We inferred range filling by calculating the proportion of suitable habitat actually occupied by the species (clipped/unclipped model ratio), after excluding the non-modeled species (with 1–2 data points). Range filling differed significantly between amphibians and reptiles, with amphibians filling on average a smaller proportion of the estimated suitable range (0.472 ± 0.290 vs. 0.613 ± 0.310; t-test, P < 0.001). Both in amphibians and reptiles, range filling was significantly correlated with SVL (parametric correlation; r = 0.216; P = 0.002 and r = 0.290; P < 0.001; non-parametric correlation; R = 0.255; P = 0.001 and R = 0.275; P < 0.001; Fig 7). Controlling for SVL in an ANCOVA, the difference between the taxonomic groups was maintained (i.e., both SVL and taxonomic category were significant predictors: P < 0.001 and P < 0.001).

### Community meta-analysis based on herpetofauna inventories

Most of the comprehensive analyses of Madagascar's biogeography in recent years have used, as original data, full distribution ranges of native species which were either derived from original records (e.g., as minimum convex polygons) or from SDMs. Such analyses estimate the number of species occurring in a certain region or site. The actual compositions of local amphibian and reptile communities are available from numerous surveys and inventories carried out in Madagascar over the past 25 years [38]. Such inventories yield lists of species co-occurring in one small area that can be used to calculate site similarities using parsimony analysis of endemism (PAE) [29, 66]. We compared community composition with data extracted from species lists of 103 sites as originally reported in the 20 selected surveys (see Materials and Methods; S2–S4 Tables).

**Fig 8:**
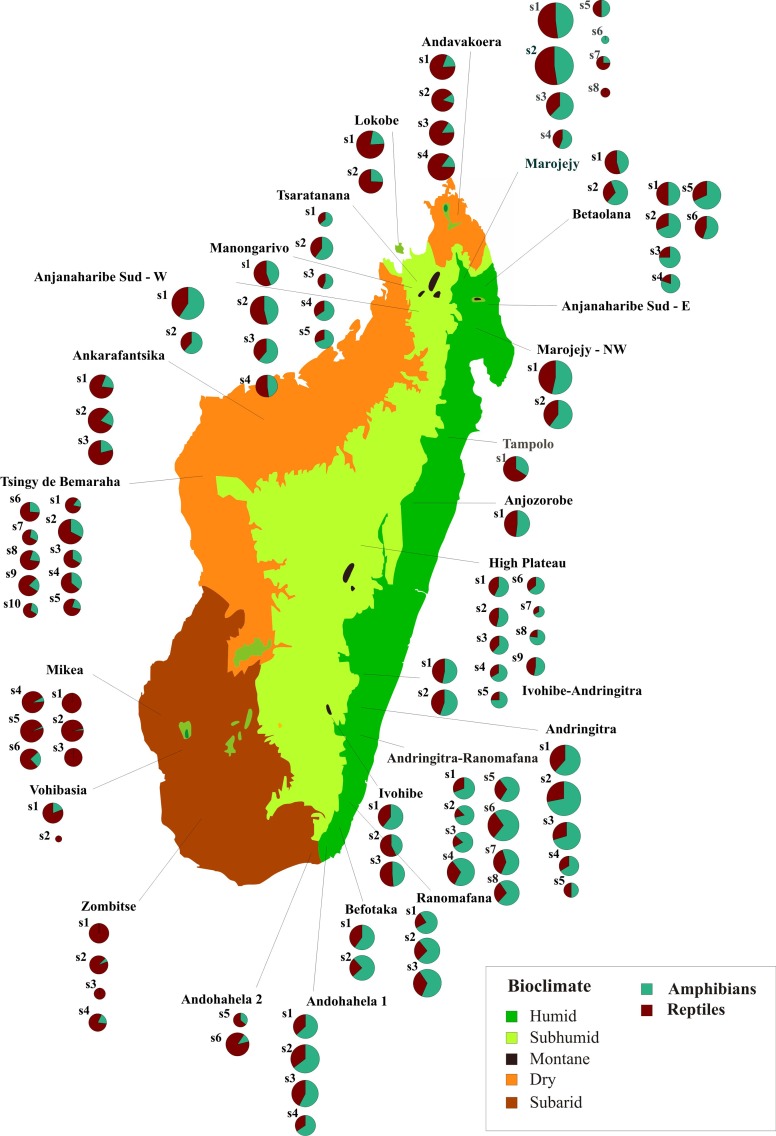
Species numbers and proportion of amphibians and reptiles recorded during herpetological inventories in Madagascar. Each pie chart represents one surveyed site (i.e., the area around one campsite at a specific elevation) and thus a community of co-occurring amphibian and reptile species. Pie area is proportional to the number of species; proportion of amphibians vs. reptiles in the community is indicated by colors. See S1 Table for a list of sites and references.

As expected from amphibian and reptile SR (Fig 1), geographically plotting species numbers and the proportions of amphibians vs. reptiles shows a lower proportion of amphibians in the dry, and especially in the subarid biomes, compared to the humid and subhumid biomes (Fig 8). For simplified comparison (all with t-tests), we summarized data for communities from the dry and subarid biomes (as arid) vs. the humid, subhumid, and montane (as moist) biomes. As expected, the number of reptile species per community is lower in the moist sites (11.7 ± 8.1 vs. 24.0 ± 11.4; P < 0.001), whereas, the number of amphibian species is higher (18.0 ± 9.8 vs. 4.9 ± 3.8; P < 0.001). Consequently, the ratio reptiles/amphibians also differs with high significance among moist and arid sites (0.39 ± 0.15 vs. 0.84 ± 0.11; P < 0.001). However, the total number of species in the herpetofaunal communities does not differ between moist and arid sites (29.8 ± 16.5 vs. 28.9 ± 13.6; P = 0.8041).

Additional patterns apparent from these data are a higher proportion of reptiles in moist sites in northern Madagascar (as defined in Fig 1D). Comparing communities from moist sites in northern Madagascar vs. those from moist sites in the rest of the island, the number of amphibian species per community is on average lower in the northern regions (t-test: 15.5 ± 10.2 vs. 21.9 ± 7.9; P < 0.01), yielding also a weakly significant difference in the proportion among amphibians and reptiles (0.42 ± 0.16 vs. 0.35 ± 0.10; P = 0.051).

**Fig 9:**
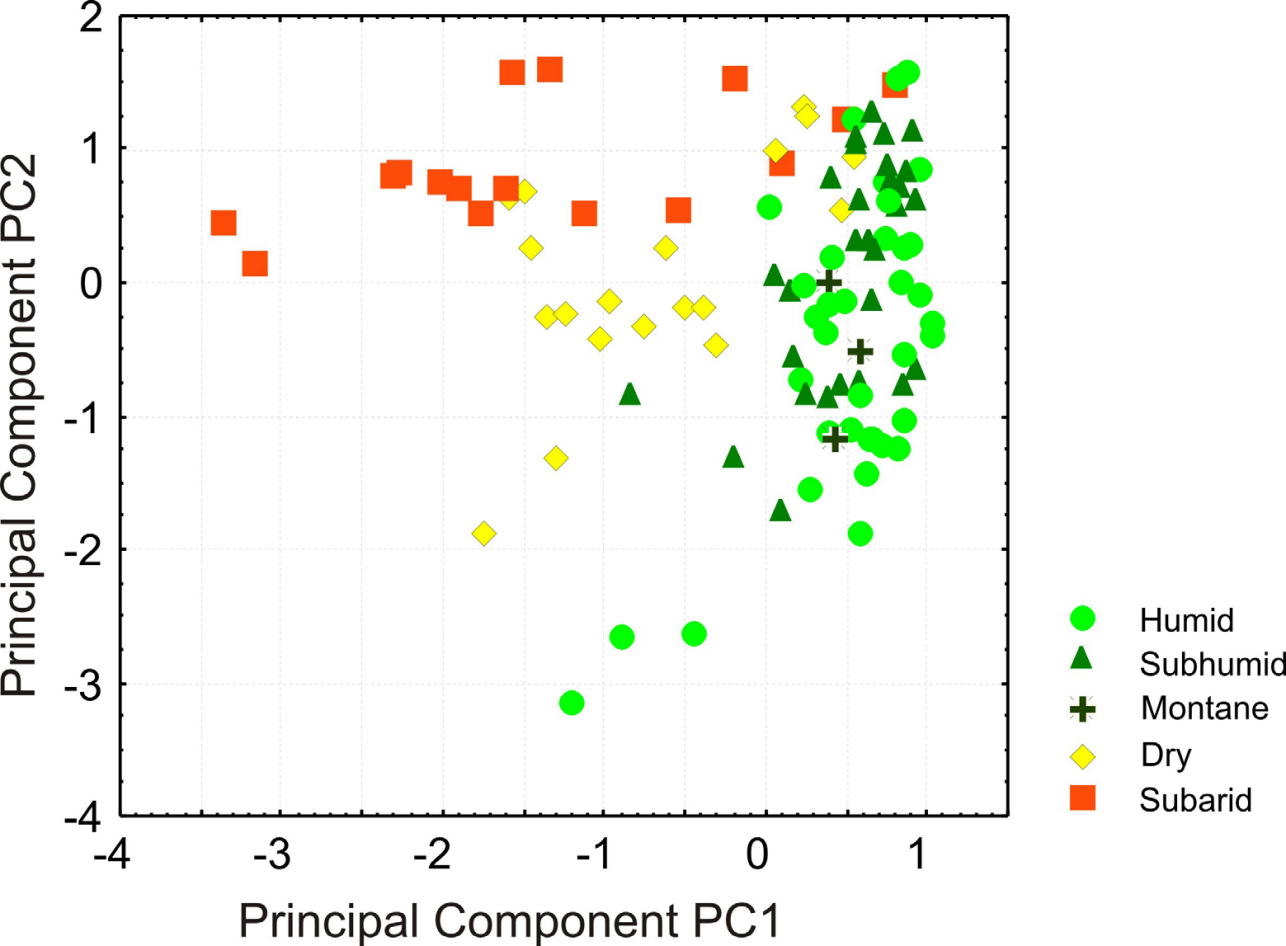
Results of a Principal Component Analysis of surveyed sites in Madagascar. Each dot represents the amphibian and reptile community at one site (see Fig 8 for a map of sites). Colors represent major bioclimatic subdivisions. PCA based on species numbers recorded for each of four major amphibian and eight major reptile groups (Table 1).

For a more detailed analysis of community composition among the different biomes, we tabulated species numbers for 12 major taxonomic groups for each of the communities and performed a Principal Component Analysis (PCA) on these data (Fig 9; Table 1). Combination of the first and second principal components (PC) separates rather well the communities from moist vs. those from arid locations (i.e., humid + subhumid + montane vs. dry + subarid biomes). The first PC separates mainly communities of the humid, subhumid and montane bioclimates from those of the arid and subarid bioclimates, with a major contribution of reptiles (all except chameleons) and non-cophyline microhylids. The second PC separates dry from subarid climates and is mainly influenced by cophyline and mantellid frogs as well as chameleons. This probably reflects the almost complete absence of mantellid and cophyline frogs, and of many chameleons, from the subarid bioclimate.

**Table 1:** Results of a Principal Component Analysis of surveyed sites in Madagascar (Fig 8), based on species numbers recorded for each of 4 major amphibian and 8 major reptile groups. Principal Components with eigenvalues >1 were extracted (PC1-PC3). Component loadings with values >0.5 are in bold.

	PC1	PC2	PC3
Hyperoliid frogs	-0.183640	0.127158	-0.957382
Microhylid frogs (non-cophylines)	**-0.584879**	0.246071	-0.079674
Microhylid frogs (cophylines)	0.321995	**-0.786310**	0.045205
Mantellid frogs	0.320439	**-0.748794**	-0.227840
Tortoises	**-0.666783**	0.259272	0.061954
Chameleons	0.002878	**-0.794360**	0.011449
Iguanas	**-0.738012**	0.384409	0.075236
Gerrhosaurids	**-0.774476**	-0.305005	-0.127244
Skinks	**-0.664216**	-0.461836	0.136841
Geckos	**-0.761727**	-0.308519	0.023604
Lamprophiid snakes	**-0.744714**	-0.312073	-0.058414
Blindsnakes	**-0.822065**	-0.132442	0.122052
Eigenvalue	4.423053	2.618131	1.040295
% Total variance	36.85877	21.81776	8.66912
Cumulative Eigenvalue	4.423053	7.041183	8.081478
Cumulative %	36.85877	58.67653	67.34565

We used the ground-truthed encountered communities in a comparison with theoretical (model-based) communities obtained by an overlap of distribution ranges. For each of the 103 survey sites, we calculated the number of species that theoretically should occur at this site based on the clipped SDMs. Then, we calculated for various taxonomic groups a ratio of encountered species number vs. theoretical species numbers. To avoid a large number of missing data and null divisions, we merged cophyline and non-cophyline microhylids and excluded hyperoliid frogs from the analysis. The obtained ratios differ among taxonomic groups (Kruskal-Wallis-ANOVA; P = 0.018) and means range from 0.18 to 0.33. The lowest values correspond to lamprophiid snakes and the highest values to chelonians, iguanas, and blindsnakes. Amphibians yielded similar values as reptiles, with a lower average in microhylids than in mantellids.

## Discussion

### Scope and limits of this study

This study provides insights into the spatial biodiversity patterns of Malagasy amphibians and reptiles, based on explicitly modeled distributions of a near-complete set of species. We illustrate how herpetofaunal communities are structured according to main bioclimatic regions, and demonstrate an important effect of body size on range size in these animals. Previous studies have analysed spatial species richness of amphibians and reptiles in Madagascar based on unmodeled distribution areas reconstructed by expert opinion[36, 37], used only partial sets of taxa [16, 22, 31, 35, 44, 45], or used the models in a hypothesis-testing framework without comparatively analyzing the patterns among groups in detail [25]. The current study is not to be seen as an exhaustive and final analysis of one or a few biogeographic questions, but rather as a baseline for future work which in part points to interesting phenomena that require in-depth study.

We are aware of several restrictions in our dataset and results based on this. While our dataset was compiled and analyses being carried out, numerous novel distribution records became available which in some cases will lead to future modifications of some minor aspects of our results (e.g., *Boophis* CWE at Bemaraha). Most important, however, are the effects of taxonomic uncertainty, different evolutionary ages of species, and different species criteria applied in different groups of taxa. Therefore, the units of analysis (the species) used in this and most other biogeographical and macroecological analyses are not fully equivalent. Several species accepted as valid, such as the frogs *Mantella viridis*, *M*. *milotympanum*, *M*. *nigricans*, or the lizards *Zonosaurus haraldmeieri* and *Z*. *trilineatus*, might rather be considered as colour variants or subspecies, whereas other species contain deep mitochondrial lineages that might turn out to correspond to distinct species upon taxonomic revision. In order to include the full dataset of species and occurrence records, many of the observation records used have not been precisely dated, posing a second limitation to this study. Given continued climate change and the widespread decrease of natural vegetation over time, considering such temporal information would certainly improve the quality of the resulting models [35].

Furthermore, our method of estimating elevational species richness relies on the clipped species distribution models and therefore assesses the number of species predicted to have suitable habitat at a certain elevation (vs. true measurements). We applied this method rather than directly extracting minimum and maximum elevations from point distributions due to issues associated with the uneven sampling of species across Madagascar that can lead to underestimating the altitudinal ranges of many understudied species (as the roads that provide access to habitats typically occur in regions of lower topographic complexity and lower elevation). Further, because SDMs model the species’ ecological tolerances, not geographic or altitudinal ranges, the modeled altitudinal ranges are not implicitly affected by altitudinal changes associated with latitude (which would also be sensitive spatial sampling biases). However, it is clear that the realized elevational niches of most of Madagascar's amphibians and reptiles are narrower than suggested by their ecological tolerances (likely due to dispersal limitations and historic climate change). Due to this over-prediction, the species numbers for any elevation (Fig 5) are likely exaggerated. However, because such a bias will be equally likely for all species, we consider the general trends, and the observed differences between amphibians and reptiles, to be reliable.

In some groups especially of reptiles, the effect of possibly inaccurate SDMs has been further exacerbated by taxon exclusions due to taxonomic uncertainty, leading to awkward aspects in the respective SR or CWE maps (Figs 3 and 4). For instance, the lack of CWE peaks of *Uroplatus* in northern Madagascar is caused by the exclusion from our study of several of the recently identified, yet, still poorly defined candidate species of this genus [82], many of which are northern endemics. As a second example, the genus *Paroedura* has been subject to intense taxonomic revisions in the past years (e.g., [83]), but much of the recent advances in knowledge on these geckos, especially in northern Madagascar, are not yet reflected in our dataset.

One last restriction regards the comparison of different field surveys from the literature. Although most of these followed similar search methods, they differed in the number of days employed to search a particular site, and in the size of the field team. Regrettably, as a result of these factors, search effort is difficult to quantify. Variation in search effort among sites certainly could have influenced the total species numbers, and to lesser degree, the proportion of different taxonomic groups that were used for our PCA.

Despite these restrictions, we are convinced that the results presented here reflect true biological patterns. In fact several of the major findings, such as the center of amphibian species richness in the Northern and Southern Central East, have remained stable since the pioneering study first reporting on the phenomenon [31]. This constancy in revealing the pattern is remarkable because the previous study[31] only included a fraction of the total number of amphibian species known today (97 vs. 325 amphibian species), and was not based on explicit distribution area modelling.

### Patterns of richness, endemism and turnover

Amphibian SR of some clades peaks in the Central East of Madagascar (others also in the North East), while the South East in all amphibian clades is comparably species-poor. This might be due to a lower integrated survey effort in particular areas such as the Anosy Mountain chain, Kalambatritra or Befotaka-Midongy Reserve, but at least partly probably reflects a biological pattern.

All amphibian clades coincide in having their highest values of SR in rainforest, with differences concerning the latitudinal location of the peaks. In reptiles, the pattern is more disparate among clades, as different clades have their SR peaking in the humid, subhumid, dry or subarid biomes. This reflects that some reptile clades did not or poorly adapt to rainforest [10], but diversified in other in other biomes only where they show high SR (Fig 3), together with the fact that more reptile radiations colonized Madagascar compared to amphibians. Regional endemicity in amphibians and reptiles varies among clades, but a common pattern of the majority of major clades and subclades is a high endemicity in northern Madagascar.

The spatial distribution of SR in amphibians suggests a clear mid-domain effect as previously postulated [16, 31]. Although this effect has been controversially discussed for the Madagascar example [84, 85], and a latitudinal mid-domain effect did not exert an important contribution to a multivariate model of Madagascar's amphibian richness [25], it is unequivocally observed that the central mid-altitudinal rainforests harbors the highest overall richness of amphibians. This is also reflected by the high numbers locally occurring in this area. Around 100 regionally sympatric amphibian species are known from comparatively small areas (less than 1000 km^2^, [25]) around Andasibe and Ranomafana, respectively [40]. The disparity of patterns among different amphibian clades are a strong indication for the absence of a major bias, e.g. in survey intensity, causing the overall central concentration of SR, which we therefore see as a true biological pattern characterizing some amphibian clades. In part, the high species richness in this area might be caused by local endemics as suggested by the high number of turnover boundaries inferred by GDM in the subhumid biome (Fig 2).

Several areas of high species turnover identified by the GDM maps (Fig 2D and 2H) agree remarkably well with the bioclimatic zonation of Schatz [47] as represented in Fig 1B. This applies in particular for the boundaries between the humid/subhumid vs. dry/subarid biomes, and even more of the dry vs. subarid biomes. This coincidence had already been remarked by Brown et al. [25] for their analysis of the full (amphibian+reptile) dataset. It should however be taken into account (see [25]) that bioclimatic data have influenced the GDM results at two analytical steps: (i) in the calculation of SDMs, and (ii) in the interpolation of community distribution in the GDM analysis. Hence, the GDM boundaries are not fully independent from a zonation based on bioclimatic data alone. Once the distribution of Madagascar's amphibians and reptiles has been more completely mapped, it will be a fruitful perspective to analyze how closely the species turnover of these communities really matches the exact boundaries of the bioclimatic zones.

The comparatively high numbers of species observed at high elevations >2000 m seems counterintuitive at first, and certainly is, in part, caused by SDM model over-prediction, considering that only few montane specialists actually are found at such altitudes in the central massifs (Andringitra and Ankaratra; [66, 86]). However, at higher latitudes, and especially in the Tsaratanana Massif in northern Madagascar, rainforest extends into higher elevation and many more amphibians and reptiles can be found >2000 m a.s.l. Hence, when interpreting the graphics in Fig 5, it is important to keep in mind that these are calculated over the entire latitudinal and longitudinal range and considering all biomes of the island. This also provides a straightforward explanation for the higher SR of reptiles at lower elevations, and the absence of this pattern in amphibians. The arid and subarid biomes of Madagascar, with partly high reptile SR, but consistently low amphibian SR, are mainly made up by low elevations including almost the entire western coastline. Many reptiles occurring in these biomes contribute to the high reptile SR seen at low elevations (Fig 5).

The distinct reductions of amphibian SR at the elevational steps between 2000–2100, and again from 2500–2600 m, correspond to the rainforest tree lines in the central massifs (at ca. 2000–2100 m) and in the Tsaratanana mountain (at ca. 2600 m), but also reflect simply the fact that very little surface area for occupancy is available above these altitudes.

### Body size influences on biogeographic pattern

The correlation between body size and range size is a well-established macroecological pattern [87, 88]. It has been previously found in Malagasy anurans [20], where small body sizes favor genetic diversification processes of anurans [20, 46]. Still, despite the availability of large numbers of range maps, range-body size relationships remain understudied in amphibians and reptiles. Although intuitively obvious from the existence of many microendemic species with tiny body sizes, e.g., in *Brookesia* or *Stumpffia* [89, 90], we here provide the first comprehensive confirmation of this correlation in Malagasy amphibians and reptiles.

**Fig 10:**
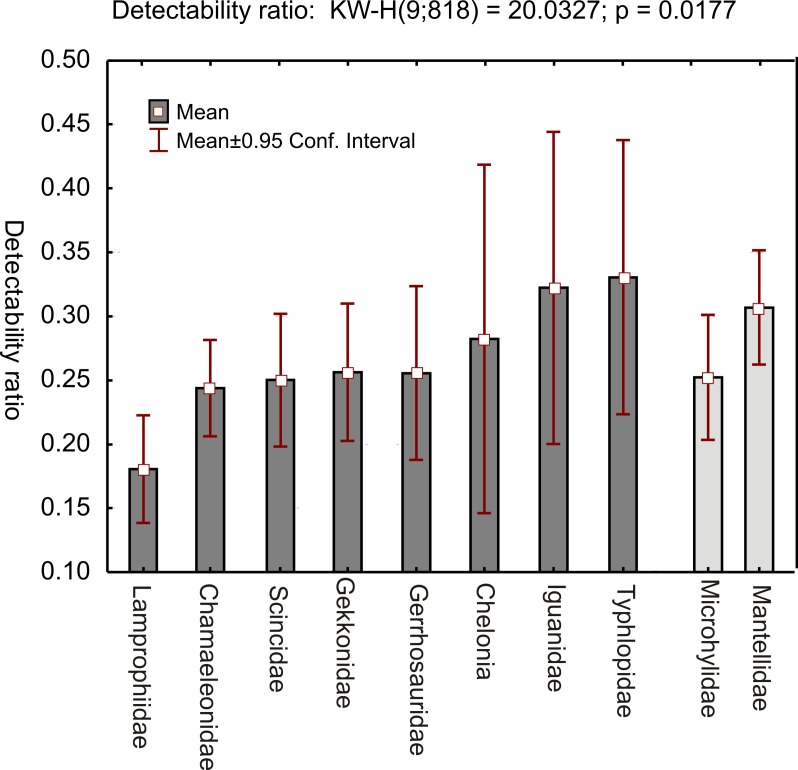
Graph showing relative observation probability of main categories of amphibian and reptile species in survey sites across Madagascar. Bars show the percentage of species of each category found per site, relative to the respective number of species theoretically occurring at these sites based on overlap of clipped SDMs. Low values indicate groups that are either difficult to detect during surveys, or are ecological specialists occurring patchily across their range.

Range sizes of snakes have previously been observed to be larger than those of lizards [91–93] (Fig 10). We here confirm this pattern for the full assemblage of species occurring in Madagascar, and provide evidence that, apparently, it is not caused only by larger body sizes of snakes. Analyzing this phenomenon in more detail and testing its possible causes is a promising perspective for future studies. Although our data seem to indicate that range size differences between amphibians and reptiles might be caused mainly by the smaller body size of amphibians, more detailed future analysis of this pattern is warranted and should for instance seek for differences within biomes. However, the differences in range filling between the two groups are apparently not caused solely by body size differences. The lower proportion of suitable distribution area occupied by amphibians probably reflects an overall lower vagility and dispersal capability, in turn probably caused by a higher sensitivity to microecological factors [94]. The Madagascar example, with a maximum of five clades of amphibians, but more than 15 clades of reptiles reaching the island after its geographic isolation [10], confirms that overall dispersal capacity is on average smaller in amphibians. Caution should be applied, however, when generalizing this difference because some amphibians, both in temperate regions (examples in [95, 96]) and in Madagascar [97], are known to have expanded their distribution areas rapidly.

### Community composition

We found strong evidence that the herpetofaunal communities encountered in Madagascar on the basis of survey work have a taxonomic composition structured predominantly along a moisture gradient across the island. Communities from humid, subhumid and montane biomes differ along the main PC from those in the dry and subarid biomes. These two latter categories partly separated along the second PC axis. This finding further validates the use of bioclimatic data to interpolate theoretical community composition in the GDM analysis. Species richness of both amphibians and reptiles peaks in the humid biome, but contrary to the expectations, the actual herpetofaunal communities are not significantly more species rich in this biome or in the ecotone connecting humid, subhumid and montane biomes, when compared to the dry and subarid biomes. No straightforward explanation for this pattern exists. However, rainforest species might be more specialized to particular microhabitats: while occurring within a general rainforest area, they might not be present at particular sites within this area if their required microhabitat is missing. Alternatively, species might be more difficult to observe in the wild at sites in moist vs. dry biomes.

Differences between taxonomic categories in the proportion of theoretical community size vs. size of communities in the field (Fig 10) can be explained by two main factors. First, it is possible that some taxa are simply more difficult to detect than others, despite being present in similar densities at a site. Second, species belonging to some higher taxa simply might be on average rarer, occurring in lower densities or more specialized to particular microhabitats. The surveys included in our analyses were all carried out by experienced teams of researchers employing a variety of search techniques, including diurnal and nocturnal opportunistic searches and in almost all cases, pitfall trapping. Still, some taxa require a long and painstaking individualized search effort, such as small-sized leaf litter frogs, common in the Cophylinae, which often require hours searching for a single calling male specimen. The lower proportion of encountered microhylids (Fig 10) likely is the result of lower survey detectability, whereas the low number of snake observations either relates to low detectability or to a possibly lower density of these predators. Surprisingly, the detection probability of blindsnakes is comparatively high, probably reflecting that these secretive animals are readily collected by pitfall trapping, or that the true distribution ranges of these animals are underestimated by our SDM approach.

## Conclusion and Outlook

By revealing a series of biogeographic patterns in Madagascar's herpetofauna this study points to promising fields for future research. Using generalized dissimilarity modeling we found a remarkable coincidence of turnover patterns of amphibians and reptiles with bioclimatic regions. This pattern warrants further exploration using ground-truthed data of community composition across the boundary of bioclimatic zones. Northern Madagascar stands out as a center of SR and CWE for numerous amphibian and reptiles clades suggesting that surveys in many of the poorly explored northern massifs might yield novel discoveries of species unknown to science. Investigating contact and hybrid zones in northern Madagascar will yield insights into the role of in-situ speciation generating this astonishing regional diversity, possibly triggered by both vicariance and adaptive divergence across ecotones or elevational bands. A closer look at range size versus body size relationships will identify those taxa deviating from the general correlation, and point to intrinsic and extrinsic factors that might make these taxa particularly weak or strong in dispersal capacity. Eventually, further substantial refinement of these biogeographic studies will greatly benefit from continued survey and collection work in Madagascar, and from taxonomic revisions improving our knowledge of the baseline distributional data.

## Supporting Information

S1 Information Figure copyright information. (DOC)

S1 Table List of species, body size (maximum male snout-vent length), number of distribution records, size of original and trimmed SDM, and range filling of amphibian and reptile species used for analysis. (DOC)

S2 Table Summary of survey data used in the meta-analysis and references for the original data. (DOC)

S3 Table Metadata and geographical location of herpetofaunal communities used for analysis. Data extracted from surveys listed in S1 Table.
(DOC)

S4 Table Species numbers of major amphibian and reptile groups used for comparison of herpetofaunal communities. Data extracted from surveys listed in S1 Table.
(DOC)
