# Supplementary material for: Spatial Biodiversity Patterns of Madagascar's Amphibians and Reptiles
Source: PLoS One. 2016 Jan 6;11(1):e0144076. doi: 10.1371/journal.pone.0144076 (PMC4703303; doi:10.1371/journal.pone.0144076)
Supplement: S1 Information — (DOC) [file pone.0144076.s001.doc]

**Spatial biodiversity patterns of Madagascar's amphibians and reptiles**

Jason Brown, Neftali Sillero, Frank Glaw, Parfait Bora, David R. Vieites, Miguel Vences

**Supplementary Materials**

**S5 Supporting Information**

PLOS ONE has permission to publish Figures 1, 2, 3, 4 and 8 under the specific Creative Commons Attribution License (CCAL), CC BY 3.0 in the manuscript entitled "Spatial biodiversity patterns of Madagascar's amphibians and reptiles".
