# Supplementary material for: Spatial Biodiversity Patterns of Madagascar's Amphibians and Reptiles
Source: PLoS One. 2016 Jan 6;11(1):e0144076. doi: 10.1371/journal.pone.0144076 (PMC4703303; doi:10.1371/journal.pone.0144076)
Supplement: S1 Table — (DOC) [file pone.0144076.s002.doc]

**Spatial biodiversity patterns of Madagascar's amphibians and reptiles**

Jason Brown, Neftali Sillero, Frank Glaw, Parfait Bora, David R. Vieites, Miguel Vences

**Supplementary Materials**

**S1 Table.** List of species, body size (maximum male snout-vent length), number of distribution records, size of original and trimmed SDM, and range filling of amphibian and reptile species used for analysis.

| **Class** | **Family** | **Species** | **Max male SVL(mm)** | **# unique pts** | **Area in BMCP SDMs (km2)** | **Area in Raw SDMs (KM2)** | **Range filling** |
| --- | --- | --- | --- | --- | --- | --- | --- |
| Amphibia | Dicroglossidae | Hoplobatrachus tigerinus | 120 | 7 | 48762 | 89855 | 0.54267 |
| Amphibia | Hyperoliidae | Heterixalus alboguttatus | 25 | 8 | 50026 | 160649 | 0.31140 |
| Amphibia | Hyperoliidae | Heterixalus andrakata | 29 | 3 | 30998 | 147526 | 0.21012 |
| Amphibia | Hyperoliidae | Heterixalus betsileo | 29 | 29 | 100908 | 115023 | 0.87729 |
| Amphibia | Hyperoliidae | Heterixalus boettgeri | 25 | 9 | 12163 | 44661 | 0.27234 |
| Amphibia | Hyperoliidae | Heterixalus carbonei | 26 | 5 | 230290 | 485692 | 0.47415 |
| Amphibia | Hyperoliidae | Heterixalus luteostriatus | 29 | 14 | 233236 | 306729 | 0.76040 |
| Amphibia | Hyperoliidae | Heterixalus madagascariensis | 24 | 12 | 18247 | 22619 | 0.80673 |
| Amphibia | Hyperoliidae | Heterixalus punctatus | 24 | 5 | 76802 | 191679 | 0.40068 |
| Amphibia | Hyperoliidae | Heterixalus rutenbergi | 27 | 9 | 62056 | 128228 | 0.48395 |
| Amphibia | Hyperoliidae | Heterixalus tricolor | 26 | 5 | 159896 | 307996 | 0.51915 |
| Amphibia | Hyperoliidae | Heterixalus variabilis | 31 | 4 | 5080 | 6328 | 0.80278 |
| Amphibia | Mantellidae | Aglyptodactylus laticeps | 45 | 1 | 314 | 314 | 1.00000 |
| Amphibia | Mantellidae | Aglyptodactylus madagascariensis | 41 | 7 | 187460 | 266370 | 0.70376 |
| Amphibia | Mantellidae | Aglyptodactylus securifer | 35 | 10 | 167603 | 259098 | 0.64687 |
| Amphibia | Mantellidae | Aglyptodactylus sp. 2 | NA | 2 | 422 | 422 | 1.00000 |
| Amphibia | Mantellidae | Aglyptodactylus sp. 3 | 35 | 3 | 30033 | 47154 | 0.63691 |
| Amphibia | Mantellidae | Blommersia angolafa | 21 | 1 | 314 | 314 | 1.00000 |
| Amphibia | Mantellidae | Blommersia blommersae | 21 | 21 | 146079 | 153259 | 0.95315 |
| Amphibia | Mantellidae | Blommersia dejongi | 20.8 | 2 | 342 | 342 | 1.00000 |
| Amphibia | Mantellidae | Blommersia domerguei | 17 | 8 | 80405 | 145036 | 0.55438 |
| Amphibia | Mantellidae | Blommersia galani | 24.2 | 4 | 10200 | 18085 | 0.56401 |
| Amphibia | Mantellidae | Blommersia grandisonae | 23 | 14 | 155793 | 177812 | 0.87617 |
| Amphibia | Mantellidae | Blommersia kely | 16 | 3 | 31267 | 59492 | 0.52556 |
| Amphibia | Mantellidae | Blommersia sarotra | 15.9 | 5 | 19698 | 75430 | 0.26114 |
| Amphibia | Mantellidae | Blommersia sp. 11 | 20.1 | 2 | 323 | 323 | 1.00000 |
| Amphibia | Mantellidae | Blommersia sp. 5 | 26 | 8 | 137521 | 273901 | 0.50208 |
| Amphibia | Mantellidae | Blommersia sp. 9 | 16 | 1 | 314 | 314 | 1.00000 |
| Amphibia | Mantellidae | Blommersia wittei | 26 | 15 | 57246 | 77644 | 0.73730 |
| Amphibia | Mantellidae | Boehmantis microtympanum | 80 | 9 | 17176 | 155628 | 0.11036 |
| Amphibia | Mantellidae | Boophis albilabris | 73 | 18 | 47554 | 75390 | 0.63077 |
| Amphibia | Mantellidae | Boophis albipunctatus | 33 | 4 | 60409 | 108675 | 0.55587 |
| Amphibia | Mantellidae | Boophis andohahela | 30 | 8 | 43502 | 145549 | 0.29888 |
| Amphibia | Mantellidae | Boophis andrangoloaka | 29 | 1 | 314 | 314 | 1.00000 |
| Amphibia | Mantellidae | Boophis andreonei | 33 | 7 | 88058 | 222704 | 0.39540 |
| Amphibia | Mantellidae | Boophis anjanaharibeensis | 36 | 2 | 628 | 628 | 1.00000 |
| Amphibia | Mantellidae | Boophis ankaratra | 28 | 13 | 57501 | 83149 | 0.69155 |
| Amphibia | Mantellidae | Boophis arcanus | 32 | 1 | 314 | 314 | 1.00000 |
| Amphibia | Mantellidae | Boophis axelmeyeri | 43 | 7 | 42388 | 179829 | 0.23571 |
| Amphibia | Mantellidae | Boophis baetkei | NA | 1 | 314 | 314 | 1.00000 |
| Amphibia | Mantellidae | Boophis blommersae | 27 | 5 | 6752 | 32469 | 0.20796 |
| Amphibia | Mantellidae | Boophis boehmei | 29 | 5 | 39667 | 176531 | 0.22471 |
| Amphibia | Mantellidae | Boophis bottae | 24 | 5 | 46799 | 66070 | 0.70833 |
| Amphibia | Mantellidae | Boophis brachychir | 50 | 3 | 14984 | 150974 | 0.09925 |
| Amphibia | Mantellidae | Boophis burgeri | 38 | 2 | 423 | 423 | 1.00000 |
| Amphibia | Mantellidae | Boophis calcaratus | 29.5 | 2 | 628 | 628 | 1.00000 |
| Amphibia | Mantellidae | Boophis doulioti | 42 | 13 | 197034 | 254689 | 0.77363 |
| Amphibia | Mantellidae | Boophis elenae | 40 | 5 | 46099 | 80398 | 0.57338 |
| Amphibia | Mantellidae | Boophis englaenderi | 35 | 5 | 38470 | 164742 | 0.23352 |
| Amphibia | Mantellidae | Boophis entingae | 50 | 4 | 63829 | 196523 | 0.32479 |
| Amphibia | Mantellidae | Boophis erythrodactylus | 25 | 4 | 133536 | 384108 | 0.34765 |
| Amphibia | Mantellidae | Boophis feonnyala | 25 | 1 | 314 | 314 | 1.00000 |
| Amphibia | Mantellidae | Boophis goudoti | 70 | 30 | 83445 | 95146 | 0.87702 |
| Amphibia | Mantellidae | Boophis guibei | 40 | 5 | 59729 | 104804 | 0.56992 |
| Amphibia | Mantellidae | Boophis haematopus | 28 | 5 | 2138 | 94337 | 0.02266 |
| Amphibia | Mantellidae | Boophis haingana | 28 | 2 | 628 | 628 | 1.00000 |
| Amphibia | Mantellidae | Boophis idae | 36 | 7 | 157349 | 227323 | 0.69218 |
| Amphibia | Mantellidae | Boophis jaegeri | 31 | 4 | 5247 | 16949 | 0.30959 |
| Amphibia | Mantellidae | Boophis laurenti | 33 | 3 | 1113 | 40775 | 0.02729 |
| Amphibia | Mantellidae | Boophis liami | 21 | 2 | 340 | 340 | 1.00000 |
| Amphibia | Mantellidae | Boophis lichenoides | 43 | 6 | 122352 | 161133 | 0.75932 |
| Amphibia | Mantellidae | Boophis lilianae | 18.3 | 1 | 314 | 314 | 1.00000 |
| Amphibia | Mantellidae | Boophis luciae | 31.3 | 8 | 83803 | 103876 | 0.80676 |
| Amphibia | Mantellidae | Boophis luteus | 40 | 21 | 90721 | 123057 | 0.73723 |
| Amphibia | Mantellidae | Boophis madagascariensis | 65 | 41 | 141941 | 152197 | 0.93261 |
| Amphibia | Mantellidae | Boophis majori | 24 | 6 | 26345 | 53683 | 0.49075 |
| Amphibia | Mantellidae | Boophis mandraka | 26 | 1 | 314 | 314 | 1.00000 |
| Amphibia | Mantellidae | Boophis marojezensis | 27 | 17 | 88145 | 106955 | 0.82413 |
| Amphibia | Mantellidae | Boophis miadana | 28 | 1 | 314 | 314 | 1.00000 |
| Amphibia | Mantellidae | Boophis microtympanum | 30 | 12 | 48211 | 54345 | 0.88712 |
| Amphibia | Mantellidae | Boophis miniatus | 32 | 5 | 1703 | 36352 | 0.04685 |
| Amphibia | Mantellidae | Boophis narinsi | 24 |  | 314 | 314 | 1.00000 |
| Amphibia | Mantellidae | Boophis obscurus | 81.9 | 4 | 81652 | 310970 | 0.26257 |
| Amphibia | Mantellidae | Boophis occidentalis | 52.4 | 4 | 98552 | 297332 | 0.33145 |
| Amphibia | Mantellidae | Boophis opisthodon | 57 | 10 | 60677 | 68806 | 0.88185 |
| Amphibia | Mantellidae | Boophis pauliani | 23 | 1 | 314 | 314 | 1.00000 |
| Amphibia | Mantellidae | Boophis periegetes | 75 | 5 | 26809 | 76381 | 0.35099 |
| Amphibia | Mantellidae | Boophis picturatus | 33 | 6 | 64478 | 84533 | 0.76276 |
| Amphibia | Mantellidae | Boophis popi | 31 | 1 | 314 | 314 | 1.00000 |
| Amphibia | Mantellidae | Boophis praedictus | 73 | 3 | 151874 | 279099 | 0.54416 |
| Amphibia | Mantellidae | Boophis pyrrhus | 32 | 11 | 102967 | 132362 | 0.77792 |
| Amphibia | Mantellidae | Boophis quasiboehmei | 35 | 2 | 476 | 476 | 1.00000 |
| Amphibia | Mantellidae | Boophis rappiodes | 25 | 4 | 109427 | 225672 | 0.48489 |
| Amphibia | Mantellidae | Boophis reticulatus | 35 | 11 | 77871 | 108917 | 0.71496 |
| Amphibia | Mantellidae | Boophis rhodoscelis | 28.1 | 11 | 47780 | 76093 | 0.62792 |
| Amphibia | Mantellidae | Boophis roseipalmatus | 65 | 4 | 35876 | 65980 | 0.54374 |
| Amphibia | Mantellidae | Boophis rufioculis | 35 | 2 | 358 | 358 | 1.00000 |
| Amphibia | Mantellidae | Boophis sambirano | 24 | 3 | 9081 | 156201 | 0.05814 |
| Amphibia | Mantellidae | Boophis sandrae | 48.2 | 6 | 46541 | 70920 | 0.65625 |
| Amphibia | Mantellidae | Boophis schuboeae | 26 | 2 | 372 | 372 | 1.00000 |
| Amphibia | Mantellidae | Boophis septentrionalis | 37 | 9 | 54718 | 193007 | 0.28350 |
| Amphibia | Mantellidae | Boophis sibilans | 30 | 6 | 84976 | 277138 | 0.30662 |
| Amphibia | Mantellidae | Boophis solomaso | 22 | 1 | 314 | 314 | 1.00000 |
| Amphibia | Mantellidae | Boophis sp aff pauliani Ranomafana | 23 | 1 | 314 | 314 | 1.00000 |
| Amphibia | Mantellidae | Boophis sp aff pauliani Tolagn | 23 | 1 | 314 | 314 | 1.00000 |
| Amphibia | Mantellidae | Boophis sp. 27 | 24 | 1 | 314 | 314 | 1.00000 |
| Amphibia | Mantellidae | Boophis sp. 28 | 23 | 1 | 314 | 314 | 1.00000 |
| Amphibia | Mantellidae | Boophis sp. 33 | 30 | 1 | 314 | 314 | 1.00000 |
| Amphibia | Mantellidae | Boophis spinophis | 56.5 | 1 | 314 | 314 | 1.00000 |
| Amphibia | Mantellidae | Boophis tampoka | 35 | 2 | 475 | 475 | 1.00000 |
| Amphibia | Mantellidae | Boophis tasymena | 23 | 9 | 101835 | 170309 | 0.59794 |
| Amphibia | Mantellidae | Boophis tephraeomystax | 42 | 25 | 133610 | 137818 | 0.96947 |
| Amphibia | Mantellidae | Boophis tsilomaro | 64 | 1 | 254 | 254 | 1.00000 |
| Amphibia | Mantellidae | Boophis ulftunni | 24.2 | 3 | 5220 | 132424 | 0.03942 |
| Amphibia | Mantellidae | Boophis viridis | 30 | 8 | 120226 | 227009 | 0.52961 |
| Amphibia | Mantellidae | Boophis vittatus | 25 | 4 | 12109 | 65792 | 0.18405 |
| Amphibia | Mantellidae | Boophis williamsi | 37 | 1 | 905 | 905 | 1.00000 |
| Amphibia | Mantellidae | Boophis xerophilus | 39 | 2 | 628 | 628 | 1.00000 |
| Amphibia | Mantellidae | Gephyromantis ambohitra | 37 | 4 | 3464 | 11660 | 0.29711 |
| Amphibia | Mantellidae | Gephyromantis asper | 30 | 10 | 109313 | 192931 | 0.56659 |
| Amphibia | Mantellidae | Gephyromantis atsingy | 43 | 2 | 606 | 606 | 1.00000 |
| Amphibia | Mantellidae | Gephyromantis azzurrae | 43 | 1 | 314 | 314 | 1.00000 |
| Amphibia | Mantellidae | Gephyromantis blanci | 23 | 11 | 21150 | 42162 | 0.50164 |
| Amphibia | Mantellidae | Gephyromantis boulengeri | 30 | 7 | 101569 | 189964 | 0.53467 |
| Amphibia | Mantellidae | Gephyromantis cornutus | 40 | 3 | 1323 | 25354 | 0.05220 |
| Amphibia | Mantellidae | Gephyromantis corvus | 38 | 1 | 314 | 314 | 1.00000 |
| Amphibia | Mantellidae | Gephyromantis decaryi | 29 | 2 | 412 | 412 | 1.00000 |
| Amphibia | Mantellidae | Gephyromantis eiselti | 22 | 5 | 8839 | 69433 | 0.12731 |
| Amphibia | Mantellidae | Gephyromantis enki | 21 | 6 | 16325 | 49080 | 0.33261 |
| Amphibia | Mantellidae | Gephyromantis granulatus | 45 | 14 | 55275 | 107199 | 0.51563 |
| Amphibia | Mantellidae | Gephyromantis hintelmannae | 26 | 1 | 314 | 314 | 1.00000 |
| Amphibia | Mantellidae | Gephyromantis horridus | 28 | 5 | 33272 | 154465 | 0.21540 |
| Amphibia | Mantellidae | Gephyromantis klemmeri | 21 | 6 | 1396 | 45017 | 0.03101 |
| Amphibia | Mantellidae | Gephyromantis leucocephalus | 29 | 11 | 20105 | 104280 | 0.19280 |
| Amphibia | Mantellidae | Gephyromantis leucomaculatus | 41 | 9 | 41500 | 134242 | 0.30914 |
| Amphibia | Mantellidae | Gephyromantis luteus | 43 | 15 | 106183 | 114738 | 0.92544 |
| Amphibia | Mantellidae | Gephyromantis malagasius | 23 | 6 | 27707 | 65864 | 0.42067 |
| Amphibia | Mantellidae | Gephyromantis moseri | 40 | 5 | 84067 | 224841 | 0.37390 |
| Amphibia | Mantellidae | Gephyromantis plicifer | 48 | 4 | 33328 | 204963 | 0.16260 |
| Amphibia | Mantellidae | Gephyromantis pseudoasper | 34 | 13 | 51561 | 100144 | 0.51487 |
| Amphibia | Mantellidae | Gephyromantis ranjomavo | 23.5 | 1 | 314 | 314 | 1.00000 |
| Amphibia | Mantellidae | Gephyromantis redimitus | 53 | 21 | 75064 | 89468 | 0.83901 |
| Amphibia | Mantellidae | Gephyromantis rivicola | 24 | 5 | 13990 | 22530 | 0.62096 |
| Amphibia | Mantellidae | Gephyromantis runewsweeki | 24 | 1 | 314 | 314 | 1.00000 |
| Amphibia | Mantellidae | Gephyromantis salegy | 48 | 2 | 628 | 628 | 1.00000 |
| Amphibia | Mantellidae | Gephyromantis schilfi | 29 | 2 | 473 | 473 | 1.00000 |
| Amphibia | Mantellidae | Gephyromantis sculpturatus | 43 | 7 | 120562 | 162561 | 0.74164 |
| Amphibia | Mantellidae | Gephyromantis silvanus | 31 | 2 | 486 | 486 | 1.00000 |
| Amphibia | Mantellidae | Gephyromantis sp aff ambohitra | 36 | 1 | 314 | 314 | 1.00000 |
| Amphibia | Mantellidae | Gephyromantis sp aff blanci Ro | 23 | 1 | 178 | 178 | 1.00000 |
| Amphibia | Mantellidae | Gephyromantis sp. 13 | 23 | 4 | 27920 | 77245 | 0.36144 |
| Amphibia | Mantellidae | Gephyromantis sp. 17 | 41 | 4 | 5941 | 37025 | 0.16047 |
| Amphibia | Mantellidae | Gephyromantis sp. 5 | 23 | 1 | 314 | 314 | 1.00000 |
| Amphibia | Mantellidae | Gephyromantis spinifer | 35 | 8 | 39167 | 168477 | 0.23248 |
| Amphibia | Mantellidae | Gephyromantis striatus | 24 | 7 | 25175 | 86617 | 0.29064 |
| Amphibia | Mantellidae | Gephyromantis tandroka | 39 | 2 | 473 | 473 | 1.00000 |
| Amphibia | Mantellidae | Gephyromantis thelenae | 23 | 3 | 2401 | 40226 | 0.05968 |
| Amphibia | Mantellidae | Gephyromantis tschenki | 36 | 5 | 25691 | 146054 | 0.17590 |
| Amphibia | Mantellidae | Gephyromantis ventrimaculatus | 25 | 4 | 73326 | 133404 | 0.54966 |
| Amphibia | Mantellidae | Gephyromantis verrucosus | 23 | 3 | 51139 | 140580 | 0.36377 |
| Amphibia | Mantellidae | Gephyromantis webbi | 25 | 3 | 9718 | 20081 | 0.48392 |
| Amphibia | Mantellidae | Gephyromantis zavona | 41 | 5 | 6426 | 44355 | 0.14488 |
| Amphibia | Mantellidae | Guibemantis albolineatus | 24 | 2 | 628 | 628 | 1.00000 |
| Amphibia | Mantellidae | Guibemantis bicalcaratus | 25 | 1 | 314 | 314 | 1.00000 |
| Amphibia | Mantellidae | Guibemantis cf pulcher St Luce | 25 | 1 | 153 | 153 | 1.00000 |
| Amphibia | Mantellidae | Guibemantis depressiceps | 45 | 8 | 120109 | 221860 | 0.54137 |
| Amphibia | Mantellidae | Guibemantis flavobrunneus | 33 | 2 | 490 | 490 | 1.00000 |
| Amphibia | Mantellidae | Guibemantis kathrinae | 59 | 2 | 628 | 628 | 1.00000 |
| Amphibia | Mantellidae | Guibemantis liber | 29 | 42 | 144689 | 146576 | 0.98713 |
| Amphibia | Mantellidae | Guibemantis pulcher | 25 | 24 | 95528 | 98824 | 0.96665 |
| Amphibia | Mantellidae | Guibemantis punctatus | 25 | 1 | 314 | 314 | 1.00000 |
| Amphibia | Mantellidae | Guibemantis sp. 20 | NA | 1 | 314 | 314 | 1.00000 |
| Amphibia | Mantellidae | Guibemantis sp. 3 | 24 | 1 | 314 | 314 | 1.00000 |
| Amphibia | Mantellidae | Guibemantis tasifotsy | 29 | 3 | 43241 | 246459 | 0.17545 |
| Amphibia | Mantellidae | Guibemantis timidus | 55 | 5 | 35950 | 50414 | 0.71309 |
| Amphibia | Mantellidae | Guibemantis tornieri | 51 | 7 | 110438 | 206322 | 0.53527 |
| Amphibia | Mantellidae | Guibemantis wattersoni | 25 | 1 | 188 | 188 | 1.00000 |
| Amphibia | Mantellidae | Laliostoma labrosum | 48 | 37 | 323441 | 333889 | 0.96871 |
| Amphibia | Mantellidae | Mantella aurantiaca | 24 | 4 | 2104 | 18000 | 0.11686 |
| Amphibia | Mantellidae | Mantella baroni | 30 | 35 | 44837 | 47291 | 0.94812 |
| Amphibia | Mantellidae | Mantella bernhardi | 19 | 9 | 43886 | 176269 | 0.24897 |
| Amphibia | Mantellidae | Mantella betsileo | 21 | 4 | 81268 | 281423 | 0.28877 |
| Amphibia | Mantellidae | Mantella cowani | 29 | 10 | 48004 | 101798 | 0.47156 |
| Amphibia | Mantellidae | Mantella crocea | 24 | 9 | 123142 | 197242 | 0.62432 |
| Amphibia | Mantellidae | Mantella ebenaui | 21 | 25 | 67273 | 96277 | 0.69874 |
| Amphibia | Mantellidae | Mantella expectata | 26 | 2 | 349 | 349 | 1.00000 |
| Amphibia | Mantellidae | Mantella haraldmeieri | 27 | 6 | 18377 | 171290 | 0.10729 |
| Amphibia | Mantellidae | Mantella laevigata | 29 | 9 | 52456 | 133112 | 0.39407 |
| Amphibia | Mantellidae | Mantella madagascariensis | 22 | 2 | 381 | 381 | 1.00000 |
| Amphibia | Mantellidae | Mantella manery | 29 | 1 | 314 | 314 | 1.00000 |
| Amphibia | Mantellidae | Mantella milotympanum | 23 | 3 | 1512 | 15212 | 0.09941 |
| Amphibia | Mantellidae | Mantella nigricans | 28 | 11 | 52020 | 124091 | 0.41921 |
| Amphibia | Mantellidae | Mantella pulchra | 23 | 8 | 58311 | 241431 | 0.24152 |
| Amphibia | Mantellidae | Mantella sp aff madagascariensis Moramanga | 22 | 3 | 11821 | 105955 | 0.11157 |
| Amphibia | Mantellidae | Mantella sp aff viridis Ankara | 25 | 2 | 359 | 359 | 1.00000 |
| Amphibia | Mantellidae | Mantella sp. 1 | 23 | 4 | 15540 | 306341 | 0.05073 |
| Amphibia | Mantellidae | Mantella viridis | 25 | 5 | 563 | 1342 | 0.41977 |
| Amphibia | Mantellidae | Mantidactylus aerumnalis | 27 | 11 | 87239 | 99371 | 0.87790 |
| Amphibia | Mantellidae | Mantidactylus albofrenatus | 23 | 3 | 17840 | 204435 | 0.08726 |
| Amphibia | Mantellidae | Mantidactylus alutus | 26 | 14 | 88525 | 112215 | 0.78888 |
| Amphibia | Mantellidae | Mantidactylus ambreensis | 38 | 9 | 61742 | 138170 | 0.44686 |
| Amphibia | Mantellidae | Mantidactylus argenteus | 27 | 12 | 145063 | 193601 | 0.74929 |
| Amphibia | Mantellidae | Mantidactylus bellyi | 41 | 6 | 36414 | 148779 | 0.24475 |
| Amphibia | Mantellidae | Mantidactylus betsileanus | 28 | 20 | 87372 | 114987 | 0.75985 |
| Amphibia | Mantellidae | Mantidactylus biporus | 27 | 2 | 628 | 628 | 1.00000 |
| Amphibia | Mantellidae | Mantidactylus bourgati | 40 | 3 | 731 | 18463 | 0.03960 |
| Amphibia | Mantellidae | Mantidactylus brevipalmatus | 35 | 14 | 51759 | 70746 | 0.73162 |
| Amphibia | Mantellidae | Mantidactylus charlotteae | 26 | 13 | 39369 | 81207 | 0.48481 |
| Amphibia | Mantellidae | Mantidactylus cowani | 40 | 3 | 80732 | 228737 | 0.35295 |
| Amphibia | Mantellidae | Mantidactylus curtus | 38.4 | 4 | 22195 | 42899 | 0.51737 |
| Amphibia | Mantellidae | Mantidactylus guttulatus East | 110 | 42 | 117762 | 131630 | 0.89465 |
| Amphibia | Mantellidae | Mantidactylus lugubris | 35 | 14 | 33755 | 41107 | 0.82116 |
| Amphibia | Mantellidae | Mantidactylus madecassus | 30 | 1 | 314 | 314 | 1.00000 |
| Amphibia | Mantellidae | Mantidactylus majori | 47 | 14 | 87180 | 130071 | 0.67025 |
| Amphibia | Mantellidae | Mantidactylus melanopleura | 40 | 41 | 90226 | 90642 | 0.99541 |
| Amphibia | Mantellidae | Mantidactylus multiplicatus | 24.3 | 1 | 314 | 314 | 1.00000 |
| Amphibia | Mantellidae | Mantidactylus noralottae | NA | 1 | 314 | 314 | 1.00000 |
| Amphibia | Mantellidae | Mantidactylus opiparis | 26 | 26 | 138100 | 154822 | 0.89199 |
| Amphibia | Mantellidae | Mantidactylus pauliani | 32 | 2 | 335 | 335 | 1.00000 |
| Amphibia | Mantellidae | Mantidactylus sp aff guttulatus North | 110 | 7 | 38963 | 154248 | 0.25260 |
| Amphibia | Mantellidae | Mantidactylus sp aff zipperi A | 23 | 2 | 314 | 314 | 1.00000 |
| Amphibia | Mantellidae | Mantidactylus sp. 14 | 36 | 6 | 58779 | 223450 | 0.26305 |
| Amphibia | Mantellidae | Mantidactylus sp. 18 | 40 | 1 | 314 | 314 | 1.00000 |
| Amphibia | Mantellidae | Mantidactylus sp. 19 | 40 | 6 | 1190 | 24830 | 0.04792 |
| Amphibia | Mantellidae | Mantidactylus sp. 20 | 35 | 1 | 314 | 314 | 1.00000 |
| Amphibia | Mantellidae | Mantidactylus sp. 24 | 23 | 1 | 314 | 314 | 1.00000 |
| Amphibia | Mantellidae | Mantidactylus sp. 41 | 40 | 2 | 628 | 628 | 1.00000 |
| Amphibia | Mantellidae | Mantidactylus sp. 57 | 110 | 9 | 68995 | 162432 | 0.42476 |
| Amphibia | Mantellidae | Mantidactylus tricinctus | 20 | 3 | 30377 | 284711 | 0.10670 |
| Amphibia | Mantellidae | Mantidactylus ulcerosus | 34 | 12 | 78584 | 121274 | 0.64799 |
| Amphibia | Mantellidae | Mantidactylus zipperi | 23 | 6 | 36064 | 133770 | 0.26960 |
| Amphibia | Mantellidae | Mantidactylus zolitschka | 31 | 1 | 314 | 314 | 1.00000 |
| Amphibia | Mantellidae | Spinomantis aglavei | 48 | 20 | 90137 | 94037 | 0.95853 |
| Amphibia | Mantellidae | Spinomantis bertini | 23 | 11 | 34091 | 76754 | 0.44416 |
| Amphibia | Mantellidae | Spinomantis brunae | 32 | 1 | 314 | 314 | 1.00000 |
| Amphibia | Mantellidae | Spinomantis elegans | 60 | 7 | 26187 | 73139 | 0.35804 |
| Amphibia | Mantellidae | Spinomantis fimbriatus | 39 | 4 | 69642 | 155534 | 0.44776 |
| Amphibia | Mantellidae | Spinomantis guibei | 35 | 5 | 6563 | 49667 | 0.13215 |
| Amphibia | Mantellidae | Spinomantis massi | 37 | 4 | 8378 | 97686 | 0.08576 |
| Amphibia | Mantellidae | Spinomantis microtis | 48 | 5 | 6575 | 48189 | 0.13644 |
| Amphibia | Mantellidae | Spinomantis peraccae | 44 | 16 | 58215 | 61212 | 0.95105 |
| Amphibia | Mantellidae | Spinomantis phantasticus | 38 | 4 | 42687 | 94212 | 0.45310 |
| Amphibia | Mantellidae | Spinomantis sp aff peraccae Ts | 44 | 2 | 314 | 314 | 1.00000 |
| Amphibia | Mantellidae | Spinomantis sp. 6 | 23 | 1 | 314 | 314 | 1.00000 |
| Amphibia | Mantellidae | Spinomantis sp. 7 | 23 | 1 | 314 | 314 | 1.00000 |
| Amphibia | Mantellidae | Spinomantis sp. 8 | 25 | 1 | 314 | 314 | 1.00000 |
| Amphibia | Mantellidae | Tsingymantis antitra | 54 | 2 | 406 | 406 | 1.00000 |
| Amphibia | Mantellidae | Wakea madinika | 13 | 1 | 314 | 314 | 1.00000 |
| Amphibia | Microhylidae: Cophylinae | Anodonthyla boulengeri | 22 | 16 | 80020 | 95203 | 0.84051 |
| Amphibia | Microhylidae: Cophylinae | Anodonthyla emilei | 29 | 1 | 314 | 314 | 1.00000 |
| Amphibia | Microhylidae: Cophylinae | Anodonthyla hutchisoni | 23 | 3 | 3148 | 21388 | 0.14719 |
| Amphibia | Microhylidae: Cophylinae | Anodonthyla jeanbai | 20 | 1 | 314 | 314 | 1.00000 |
| Amphibia | Microhylidae: Cophylinae | Anodonthyla montana | 34 | 4 | 748 | 11257 | 0.06648 |
| Amphibia | Microhylidae: Cophylinae | Anodonthyla moramora | 17 | 1 | 314 | 314 | 1.00000 |
| Amphibia | Microhylidae: Cophylinae | Anodonthyla nigrigularis | 24 | 6 | 4852 | 52489 | 0.09244 |
| Amphibia | Microhylidae: Cophylinae | Anodonthyla rouxae | 34 | 2 | 628 | 628 | 1.00000 |
| Amphibia | Microhylidae: Cophylinae | Anodonthyla vallani | 24 | 1 | 314 | 314 | 1.00000 |
| Amphibia | Microhylidae: Cophylinae | Cophyla berara | 26 | 1 | 254 | 254 | 1.00000 |
| Amphibia | Microhylidae: Cophylinae | Cophyla occultans | 21 | 4 | 57781 | 260361 | 0.22193 |
| Amphibia | Microhylidae: Cophylinae | Cophyla phyllodactyla | 29 | 8 | 24044 | 39396 | 0.61032 |
| Amphibia | Microhylidae: Cophylinae | Madecassophryne truebae | 23 | 1 | 314 | 314 | 1.00000 |
| Amphibia | Microhylidae: Cophylinae | Platypelis alticola | 38 | 4 | 1336 | 11066 | 0.12073 |
| Amphibia | Microhylidae: Cophylinae | Platypelis barbouri | 23 | 5 | 803 | 12350 | 0.06499 |
| Amphibia | Microhylidae: Cophylinae | Platypelis grandis | 88 | 43 | 136018 | 136724 | 0.99483 |
| Amphibia | Microhylidae: Cophylinae | Platypelis milloti | 30 | 4 | 11440 | 104005 | 0.10999 |
| Amphibia | Microhylidae: Cophylinae | Platypelis pollicaris | 28 | 4 | 48169 | 90171 | 0.53419 |
| Amphibia | Microhylidae: Cophylinae | Platypelis sp aff mavomavo Mar | 19 | 1 | 314 | 314 | 1.00000 |
| Amphibia | Microhylidae: Cophylinae | Platypelis tetra | 18 | 4 | 11510 | 157850 | 0.07292 |
| Amphibia | Microhylidae: Cophylinae | Platypelis tsaratananaensis | 28 | 8 | 28911 | 84050 | 0.34397 |
| Amphibia | Microhylidae: Cophylinae | Platypelis tuberifera | 40 | 31 | 76398 | 92732 | 0.82386 |
| Amphibia | Microhylidae: Cophylinae | Plethodontohyla bipunctata | 32 | 12 | 31498 | 71043 | 0.44337 |
| Amphibia | Microhylidae: Cophylinae | Plethodontohyla brevipes | 36 | 2 | 394 | 394 | 1.00000 |
| Amphibia | Microhylidae: Cophylinae | Plethodontohyla fonetana | 65 | 1 | 314 | 314 | 1.00000 |
| Amphibia | Microhylidae: Cophylinae | Plethodontohyla guentheri | 33 | 1 | 314 | 314 | 1.00000 |
| Amphibia | Microhylidae: Cophylinae | Plethodontohyla inguinalis | 100 | 26 | 92104 | 93125 | 0.98904 |
| Amphibia | Microhylidae: Cophylinae | Plethodontohyla mihanika | 30 | 10 | 78457 | 184192 | 0.42595 |
| Amphibia | Microhylidae: Cophylinae | Plethodontohyla notosticta | 42 | 18 | 113397 | 132204 | 0.85774 |
| Amphibia | Microhylidae: Cophylinae | Plethodontohyla ocellata | 65 | 12 | 161579 | 231529 | 0.69788 |
| Amphibia | Microhylidae: Cophylinae | Plethodontohyla sp. 1 | 36 | 4 | 1569 | 363069 | 0.00432 |
| Amphibia | Microhylidae: Cophylinae | Plethodontohyla tuberata | 45 | 5 | 56430 | 86654 | 0.65121 |
| Amphibia | Microhylidae: Cophylinae | Rhombophryne alluaudi | 60 | 7 | 211322 | 315723 | 0.66933 |
| Amphibia | Microhylidae: Cophylinae | Rhombophryne coronata | 23 | 2 | 628 | 628 | 1.00000 |
| Amphibia | Microhylidae: Cophylinae | Rhombophryne coudreaui | 28 | 5 | 21150 | 218671 | 0.09672 |
| Amphibia | Microhylidae: Cophylinae | Rhombophryne guentherpetersi | 35 | 2 | 624 | 624 | 1.00000 |
| Amphibia | Microhylidae: Cophylinae | Rhombophryne laevipes | 45 | 4 | 20215 | 34242 | 0.59034 |
| Amphibia | Microhylidae: Cophylinae | Rhombophryne mangabensis | 23 | 1 | 71 | 71 | 1.00000 |
| Amphibia | Microhylidae: Cophylinae | Rhombophryne minuta | 17 | 2 | 465 | 465 | 1.00000 |
| Amphibia | Microhylidae: Cophylinae | Rhombophryne serratopalpebrosa | 29 | 8 | 5369 | 10799 | 0.49718 |
| Amphibia | Microhylidae: Cophylinae | Rhombophryne testudo | 39 | 5 | 6306 | 130166 | 0.04845 |
| Amphibia | Microhylidae: Cophylinae | Stumpffia gimmeli | 15 | 4 | 18315 | 127906 | 0.14319 |
| Amphibia | Microhylidae: Cophylinae | Stumpffia grandis | 22 | 2 | 473 | 473 | 1.00000 |
| Amphibia | Microhylidae: Cophylinae | Stumpffia helenae | 14 | 1 | 314 | 314 | 1.00000 |
| Amphibia | Microhylidae: Cophylinae | Stumpffia psologlossa | 16 | 5 | 1893 | 5143 | 0.36807 |
| Amphibia | Microhylidae: Cophylinae | Stumpffia pygmaea | 12 | 5 | 326 | 12841 | 0.02539 |
| Amphibia | Microhylidae: Cophylinae | Stumpffia roseifemoralis | 20 | 1 | 314 | 314 | 1.00000 |
| Amphibia | Microhylidae: Cophylinae | Stumpffia sp aff gimmeli Andap | 15 | 1 | 314 | 314 | 1.00000 |
| Amphibia | Microhylidae: Cophylinae | Stumpffia sp aff helenae Bemaraha | 16 | 3 | 1397 | 25747 | 0.05426 |
| Amphibia | Microhylidae: Cophylinae | Stumpffia sp aff tridactyla Andasibe | 11 | 4 | 12914 | 60866 | 0.21217 |
| Amphibia | Microhylidae: Cophylinae | Stumpffia sp erythrogaster | 21 | 1 | 314 | 314 | 1.00000 |
| Amphibia | Microhylidae: Cophylinae | Stumpffia sp fast call Marojej | 19 | 1 | 314 | 314 | 1.00000 |
| Amphibia | Microhylidae: Cophylinae | Stumpffia sp red Ranomafana | 18 | 1 | 314 | 314 | 1.00000 |
| Amphibia | Microhylidae: Cophylinae | Stumpffia sp slow call Marojej | 18 | 2 | 314 | 314 | 1.00000 |
| Amphibia | Microhylidae: Cophylinae | Stumpffia sp Zieper Marojejy | 18 | 1 | 314 | 314 | 1.00000 |
| Amphibia | Microhylidae: Cophylinae | Stumpffia tetradactyla | 15 | 1 | 71 | 71 | 1.00000 |
| Amphibia | Microhylidae: Cophylinae | Stumpffia tridactyla | 11 | 2 | 473 | 473 | 1.00000 |
| Amphibia | Microhylidae: Dyscophinae | Dyscophus antongili | 65 | 4 | 15275 | 20282 | 0.75316 |
| Amphibia | Microhylidae: Dyscophinae | Dyscophus guineti | 65 | 7 | 152548 | 327482 | 0.46582 |
| Amphibia | Microhylidae: Dyscophinae | Dyscophus insularis | 50 | 10 | 272097 | 343845 | 0.79134 |
| Amphibia | Microhylidae: Scaphiophrynidae | Paradoxophyla palmata | 22 | 6 | 140431 | 226393 | 0.62030 |
| Amphibia | Microhylidae: Scaphiophrynidae | Paradoxophyla tiarano | 18 | 1 | 314 | 314 | 1.00000 |
| Amphibia | Microhylidae: Scaphiophrynidae | Scaphiophryne boribory | 60 | 3 | 79731 | 257825 | 0.30924 |
| Amphibia | Microhylidae: Scaphiophrynidae | Scaphiophryne brevis | 40 | 13 | 142398 | 152237 | 0.93537 |
| Amphibia | Microhylidae: Scaphiophrynidae | Scaphiophryne calcarata | 27 | 3 | 1617 | 134493 | 0.01202 |
| Amphibia | Microhylidae: Scaphiophrynidae | Scaphiophryne gottlebei | 30 | 1 | 314 | 314 | 1.00000 |
| Amphibia | Microhylidae: Scaphiophrynidae | Scaphiophryne madagascariensis | 56 | 7 | 48240 | 84783 | 0.56898 |
| Amphibia | Microhylidae: Scaphiophrynidae | Scaphiophryne marmorata | 36 | 3 | 44692 | 370276 | 0.12070 |
| Amphibia | Microhylidae: Scaphiophrynidae | Scaphiophryne matsoko | 36 | 4 | 44882 | 209164 | 0.21458 |
| Amphibia | Microhylidae: Scaphiophrynidae | Scaphiophryne menabensis | 43 | 4 | 123951 | 243849 | 0.50831 |
| Amphibia | Microhylidae: Scaphiophrynidae | Scaphiophryne sp aff calcarata Kirindy | 27 | 4 | 2241 | 2241 | 1.00000 |
| Amphibia | Microhylidae: Scaphiophrynidae | Scaphiophryne spinosa | 48 | 13 | 145322 | 155708 | 0.93330 |
| Amphibia | Ptychadenidae | Ptychadena mascareniensis | 40 | 97 | 105798 | 112598 | 0.93961 |
| Squamata | Boidae | Acrantophis dumerili | 1200 | 28 | 164447 | 182785 | 0.89967 |
| Squamata | Boidae | Acrantophis madagascariensis | 2200 | 24 | 139903 | 162132 | 0.86290 |
| Squamata | Boidae | Sanzinia madagascariensis | 1620 | 39 | 122271 | 126305 | 0.96806 |
| Squamata | Boidae | Sanzinia madagascariensis volontany | 1420 | 24 | 266736 | 267640 | 0.99662 |
| Squamata | Chamaeleonidae | Brookesia ambreensis | 52 | 1 | 314 | 314 | 1.00000 |
| Squamata | Chamaeleonidae | Brookesia antakarana | 52 | 1 | 314 | 314 | 1.00000 |
| Squamata | Chamaeleonidae | Brookesia bekolosy | 34 | 1 | 328 | 328 | 1.00000 |
| Squamata | Chamaeleonidae | Brookesia betschi | 34 | 10 | 22209 | 28238 | 0.78652 |
| Squamata | Chamaeleonidae | Brookesia bonsi | 50 | 1 | 314 | 314 | 1.00000 |
| Squamata | Chamaeleonidae | Brookesia brygooi | 48 | 17 | 57233 | 127864 | 0.44761 |
| Squamata | Chamaeleonidae | Brookesia confidens | 20 | 1 | 314 | 314 | 1.00000 |
| Squamata | Chamaeleonidae | Brookesia decaryi | 55 | 3 | 7674 | 14734 | 0.52084 |
| Squamata | Chamaeleonidae | Brookesia dentata | 22 | 2 | 628 | 628 | 1.00000 |
| Squamata | Chamaeleonidae | Brookesia desperata | 27 | 1 | 314 | 314 | 1.00000 |
| Squamata | Chamaeleonidae | Brookesia ebenaui | 60 | 6 | 8756 | 25681 | 0.34095 |
| Squamata | Chamaeleonidae | Brookesia exarmata | 18.4 | 3 | 1263 | 14588 | 0.08655 |
| Squamata | Chamaeleonidae | Brookesia griveaudi | 55 | 11 | 27249 | 84852 | 0.32114 |
| Squamata | Chamaeleonidae | Brookesia karchei | 30 | 4 | 1398 | 74702 | 0.01871 |
| Squamata | Chamaeleonidae | Brookesia lambertoni | 40 | 1 | 314 | 314 | 1.00000 |
| Squamata | Chamaeleonidae | Brookesia lineata | 45 | 4 | 2769 | 62259 | 0.04448 |
| Squamata | Chamaeleonidae | Brookesia lolontany | 32 | 1 | 314 | 314 | 1.00000 |
| Squamata | Chamaeleonidae | Brookesia micra | 16 | 1 | 94 | 94 | 1.00000 |
| Squamata | Chamaeleonidae | Brookesia minima | 18 | 3 | 2305 | 124851 | 0.01846 |
| Squamata | Chamaeleonidae | Brookesia nasus nasus | 49 | 20 | 31889 | 46707 | 0.68275 |
| Squamata | Chamaeleonidae | Brookesia perarmata | 47 | 2 | 606 | 606 | 1.00000 |
| Squamata | Chamaeleonidae | Brookesia peyrierasi | 40 | 3 | 5041 | 23054 | 0.21866 |
| Squamata | Chamaeleonidae | Brookesia ramanantsoai | 22 | 3 | 7223 | 68269 | 0.10580 |
| Squamata | Chamaeleonidae | Brookesia sp aff minima Betamp | 18 | 1 | 933 | 933 | 1.00000 |
| Squamata | Chamaeleonidae | Brookesia stumpffi | 55 | 28 | 44837 | 65408 | 0.68550 |
| Squamata | Chamaeleonidae | Brookesia superciliaris | 53 | 38 | 113097 | 117959 | 0.95878 |
| Squamata | Chamaeleonidae | Brookesia therezieni | 52 | 8 | 88892 | 207801 | 0.42777 |
| Squamata | Chamaeleonidae | Brookesia thieli | 40 | 12 | 148521 | 202233 | 0.73441 |
| Squamata | Chamaeleonidae | Brookesia tristis | 18 | 1 | 628 | 628 | 1.00000 |
| Squamata | Chamaeleonidae | Brookesia tuberculata | 19 | 1 | 314 | 314 | 1.00000 |
| Squamata | Chamaeleonidae | Brookesia vadoni | 29.5 | 8 | 35103 | 193879 | 0.18106 |
| Squamata | Chamaeleonidae | Brookesia valerieae | 53 | 2 | 576 | 576 | 1.00000 |
| Squamata | Chamaeleonidae | Calumma (globifer) ambreense | 170 | 4 | 7351 | 42440 | 0.17320 |
| Squamata | Chamaeleonidae | Calumma amber | 112 | 3 | 1021 | 2553 | 0.39986 |
| Squamata | Chamaeleonidae | Calumma andringitraense | 58 | 4 | 5707 | 34962 | 0.16323 |
| Squamata | Chamaeleonidae | Calumma boettgeri | 55 | 1 | 296 | 296 | 1.00000 |
| Squamata | Chamaeleonidae | Calumma brevicorne | 170 | 5 | 220741 | 297360 | 0.74234 |
| Squamata | Chamaeleonidae | Calumma capuroni | 90 | 2 | 628 | 628 | 1.00000 |
| Squamata | Chamaeleonidae | Calumma crypticum | 115 | 20 | 113606 | 114832 | 0.98932 |
| Squamata | Chamaeleonidae | Calumma cucullatum | 190 | 10 | 101629 | 186399 | 0.54522 |
| Squamata | Chamaeleonidae | Calumma fallax | 50 | 4 | 104530 | 336535 | 0.31061 |
| Squamata | Chamaeleonidae | Calumma furcifer | 70 | 6 | 48658 | 358258 | 0.13582 |
| Squamata | Chamaeleonidae | Calumma gallus | 60 | 3 | 3173 | 152662 | 0.02078 |
| Squamata | Chamaeleonidae | Calumma gastrotaenia | 68 | 21 | 98157 | 123671 | 0.79370 |
| Squamata | Chamaeleonidae | Calumma glawi | 65 | 7 | 8185 | 24287 | 0.33702 |
| Squamata | Chamaeleonidae | Calumma globifer | 170 | 4 | 96103 | 266654 | 0.36040 |
| Squamata | Chamaeleonidae | Calumma guibei | 55 | 2 | 370 | 370 | 1.00000 |
| Squamata | Chamaeleonidae | Calumma guillaumeti | 52 | 10 | 21810 | 31259 | 0.69770 |
| Squamata | Chamaeleonidae | Calumma hafahafa | 110 | 3 | 6619 | 14840 | 0.44599 |
| Squamata | Chamaeleonidae | Calumma hilleniusi | 70 | 1 | 314 | 314 | 1.00000 |
| Squamata | Chamaeleonidae | Calumma jejy | 96 | 1 | 314 | 314 | 1.00000 |
| Squamata | Chamaeleonidae | Calumma linotum | 55 | 5 | 13557 | 20491 | 0.66161 |
| Squamata | Chamaeleonidae | Calumma malthe | 135 | 17 | 109260 | 114954 | 0.95047 |
| Squamata | Chamaeleonidae | Calumma marojezense | 70 | 2 | 784 | 784 | 1.00000 |
| Squamata | Chamaeleonidae | Calumma nasutum | 48 | 2 | 628 | 628 | 1.00000 |
| Squamata | Chamaeleonidae | Calumma oshaughnessyi | 165 | 23 | 25782 | 35375 | 0.72881 |
| Squamata | Chamaeleonidae | Calumma parsonii | 295 | 22 | 61538 | 95772 | 0.64255 |
| Squamata | Chamaeleonidae | Calumma peltierorum | 108 | 4 | 7252 | 20624 | 0.35161 |
| Squamata | Chamaeleonidae | Calumma peyrierasi | 48 | 4 | 10010 | 48532 | 0.20626 |
| Squamata | Chamaeleonidae | Calumma sp aff gallus South | 60 | 3 | 40142 | 98809 | 0.40626 |
| Squamata | Chamaeleonidae | Calumma sp aff nasutum Andohah | 48 | 1 | 314 | 314 | 1.00000 |
| Squamata | Chamaeleonidae | Calumma sp aff nasutum Anjozorobe-Andohahela | 48 | 5 | 163736 | 218314 | 0.75000 |
| Squamata | Chamaeleonidae | Calumma sp aff nasutum Sahafin | 50 | 1 | 314 | 314 | 1.00000 |
| Squamata | Chamaeleonidae | Calumma sp aff nasutum Vohidrazana-Makira | 48 | 4 | 74577 | 186007 | 0.40094 |
| Squamata | Chamaeleonidae | Calumma tarzan | 72 | 1 | 314 | 314 | 1.00000 |
| Squamata | Chamaeleonidae | Calumma tsaratananense | 44 | 4 | 1891 | 31043 | 0.06092 |
| Squamata | Chamaeleonidae | Calumma tsycorne | 115 | 2 | 628 | 628 | 1.00000 |
| Squamata | Chamaeleonidae | Calumma vatosoa | 60 | 1 | 559 | 559 | 1.00000 |
| Squamata | Chamaeleonidae | Calumma vencesi | 73 | 3 | 2903 | 74583 | 0.03892 |
| Squamata | Chamaeleonidae | Furcifer angeli | 160 | 11 | 31505 | 72519 | 0.43443 |
| Squamata | Chamaeleonidae | Furcifer antimena | 170 | 4 | 15404 | 29514 | 0.52191 |
| Squamata | Chamaeleonidae | Furcifer balteatus | 175 | 4 | 7315 | 64822 | 0.11285 |
| Squamata | Chamaeleonidae | Furcifer bifidus | 200 | 28 | 47664 | 82121 | 0.58041 |
| Squamata | Chamaeleonidae | Furcifer campani | 139 | 22 | 31697 | 33947 | 0.93373 |
| Squamata | Chamaeleonidae | Furcifer labordi | 138 | 15 | 44549 | 54859 | 0.81207 |
| Squamata | Chamaeleonidae | Furcifer lateralis | 139 | 130 | 319641 | 327033 | 0.97740 |
| Squamata | Chamaeleonidae | Furcifer minor | 100 | 8 | 50669 | 211287 | 0.23981 |
| Squamata | Chamaeleonidae | Furcifer nicosiai | 145 | 11 | 36107 | 61470 | 0.58739 |
| Squamata | Chamaeleonidae | Furcifer oustaleti | 284 | 102 | 354904 | 355084 | 0.99949 |
| Squamata | Chamaeleonidae | Furcifer pardalis | 250 | 76 | 69872 | 73434 | 0.95150 |
| Squamata | Chamaeleonidae | Furcifer petteri | 90 | 13 | 46541 | 52242 | 0.89088 |
| Squamata | Chamaeleonidae | Furcifer rhinoceratus | 140 | 12 | 19529 | 22190 | 0.88007 |
| Squamata | Chamaeleonidae | Furcifer timoni | 88 | 1 | 314 | 314 | 1.00000 |
| Squamata | Chamaeleonidae | Furcifer tuzetae | 173 | 1 | 314 | 314 | 1.00000 |
| Squamata | Chamaeleonidae | Furcifer verrucosus | 265 | 95 | 126580 | 134001 | 0.94462 |
| Squamata | Chamaeleonidae | Furcifer willsii | 76 | 23 | 178787 | 198052 | 0.90273 |
| Squamata | Gekkonidae | Blaesodactylus antongilensis | 97 | 12 | 51672 | 271315 | 0.19045 |
| Squamata | Gekkonidae | Blaesodactylus boivini | 132 | 5 | 6557 | 23873 | 0.27465 |
| Squamata | Gekkonidae | Blaesodactylus sakalava | 104 | 10 | 207713 | 217089 | 0.95681 |
| Squamata | Gekkonidae | Ebenavia inunguis | 42 | 45 | 136781 | 137161 | 0.99723 |
| Squamata | Gekkonidae | Ebenavia maintimainty | 24 | 2 | 422 | 422 | 1.00000 |
| Squamata | Gekkonidae | Geckolepis anomala | 43 | 2 | 628 | 628 | 1.00000 |
| Squamata | Gekkonidae | Geckolepis maculata | 70 | 32 | 246944 | 251073 | 0.98355 |
| Squamata | Gekkonidae | Geckolepis petiti | 37 | 1 | 314 | 314 | 1.00000 |
| Squamata | Gekkonidae | Geckolepis polylepis | 51 | 4 | 142215 | 188918 | 0.75279 |
| Squamata | Gekkonidae | Geckolepis typica | 63 | 11 | 294545 | 331324 | 0.88899 |
| Squamata | Gekkonidae | Gehyra mutilata | 60 | 10 | 167784 | 246647 | 0.68026 |
| Squamata | Gekkonidae | Hemidactylus frenatus | 55 | 24 | 194688 | 201187 | 0.96770 |
| Squamata | Gekkonidae | Hemidactylus mercatorius | 56 | 39 | 338413 | 340384 | 0.99421 |
| Squamata | Gekkonidae | Hemidactylus platycephalus | 85 | 7 | 27254 | 45172 | 0.60333 |
| Squamata | Gekkonidae | Lygodactylus arnoulti | 37 | 3 | 9742 | 53824 | 0.18100 |
| Squamata | Gekkonidae | Lygodactylus blancae | 35 | 1 | 314 | 314 | 1.00000 |
| Squamata | Gekkonidae | Lygodactylus blanci | 39 | 1 | 314 | 314 | 1.00000 |
| Squamata | Gekkonidae | Lygodactylus decaryi | 27 | 2 | 628 | 628 | 1.00000 |
| Squamata | Gekkonidae | Lygodactylus expectatus | 30 | 2 | 369 | 369 | 1.00000 |
| Squamata | Gekkonidae | Lygodactylus guibei | 38 | 4 | 37544 | 225100 | 0.16679 |
| Squamata | Gekkonidae | Lygodactylus heterurus | 25 | 5 | 24477 | 59219 | 0.41333 |
| Squamata | Gekkonidae | Lygodactylus intermedius | 31 | 3 | 1420 | 93824 | 0.01513 |
| Squamata | Gekkonidae | Lygodactylus klemmeri | 25 | 5 | 1412 | 13638 | 0.10356 |
| Squamata | Gekkonidae | Lygodactylus madagascariensis | 37 | 10 | 42718 | 69147 | 0.61779 |
| Squamata | Gekkonidae | Lygodactylus miops | 33 | 28 | 113916 | 117555 | 0.96904 |
| Squamata | Gekkonidae | Lygodactylus mirabilis | 29 | 2 | 369 | 369 | 1.00000 |
| Squamata | Gekkonidae | Lygodactylus montanus | 38 | 4 | 27631 | 122502 | 0.22555 |
| Squamata | Gekkonidae | Lygodactylus ornatus | 26 | 1 | 314 | 314 | 1.00000 |
| Squamata | Gekkonidae | Lygodactylus pauliani | 36 | 1 | 406 | 406 | 1.00000 |
| Squamata | Gekkonidae | Lygodactylus pictus | 38 | 10 | 62689 | 115085 | 0.54472 |
| Squamata | Gekkonidae | Lygodactylus praecox | NA | 1 | 314 | 314 | 1.00000 |
| Squamata | Gekkonidae | Lygodactylus rarus | 37 | 2 | 628 | 628 | 1.00000 |
| Squamata | Gekkonidae | Lygodactylus roavolana | 34 | 1 | 291 | 291 | 1.00000 |
| Squamata | Gekkonidae | Lygodactylus tolampyae | 35 | 10 | 234238 | 292308 | 0.80134 |
| Squamata | Gekkonidae | Lygodactylus tuberosus | 38 | 17 | 60239 | 61201 | 0.98428 |
| Squamata | Gekkonidae | Lygodactylus verticillatus | 27 | 5 | 36854 | 49021 | 0.75181 |
| Squamata | Gekkonidae | Matoatoa brevipes | 35 | 2 | 830 | 830 | 1.00000 |
| Squamata | Gekkonidae | Matoatoa spannringi | 57 | 1 | 314 | 314 | 1.00000 |
| Squamata | Gekkonidae | Microscalabotes bivittis | 36 | 3 | 97861 | 320349 | 0.30548 |
| Squamata | Gekkonidae | Paragehyra gabriellae | 75 | 8 | 6694 | 66987 | 0.09993 |
| Squamata | Gekkonidae | Paragehyra petiti | 59 | 2 | 497 | 497 | 1.00000 |
| Squamata | Gekkonidae | Paroedura androyensis | 47 | 7 | 95618 | 203703 | 0.46940 |
| Squamata | Gekkonidae | Paroedura bastardi | 80 | 25 | 139297 | 154993 | 0.89873 |
| Squamata | Gekkonidae | Paroedura gracilis | 67 | 21 | 67836 | 87496 | 0.77531 |
| Squamata | Gekkonidae | Paroedura homalorhina | 64 | 3 | 68310 | 245732 | 0.27799 |
| Squamata | Gekkonidae | Paroedura ibityensis | 61 | 2 | 628 | 628 | 1.00000 |
| Squamata | Gekkonidae | Paroedura karstophila | 55 | 3 | 13364 | 52923 | 0.25253 |
| Squamata | Gekkonidae | Paroedura lohatsara | 73 | 2 | 393 | 393 | 1.00000 |
| Squamata | Gekkonidae | Paroedura maingoka | 71 | 2 | 393 | 393 | 1.00000 |
| Squamata | Gekkonidae | Paroedura masobe | 107 | 1 | 314 | 314 | 1.00000 |
| Squamata | Gekkonidae | Paroedura oviceps | 69 | 12 | 66275 | 127704 | 0.51898 |
| Squamata | Gekkonidae | Paroedura picta | 90 | 15 | 115529 | 120056 | 0.96229 |
| Squamata | Gekkonidae | Paroedura sp aff karstophila A | 55 | 1 | 314 | 314 | 1.00000 |
| Squamata | Gekkonidae | Paroedura stumpffi | 70 | 15 | 44171 | 51436 | 0.85875 |
| Squamata | Gekkonidae | Paroedura tanjaka | NA | 9 | 16284 | 45659 | 0.35663 |
| Squamata | Gekkonidae | Paroedura vahiny | 42 | 3 | 28578 | 56175 | 0.50873 |
| Squamata | Gekkonidae | Paroedura vazimba | 49 | 2 | 809 | 809 | 1.00000 |
| Squamata | Gekkonidae | Phelsuma abbotti chekei | 60 | 16 | 78500 | 95390 | 0.82293 |
| Squamata | Gekkonidae | Phelsuma antanosy | 48 | 3 | 2598 | 9353 | 0.27775 |
| Squamata | Gekkonidae | Phelsuma barbouri | 64 | 8 | 14113 | 15310 | 0.92181 |
| Squamata | Gekkonidae | Phelsuma berghofi | 58 | 2 | 575 | 575 | 1.00000 |
| Squamata | Gekkonidae | Phelsuma borai | 42 | 2 | 628 | 628 | 1.00000 |
| Squamata | Gekkonidae | Phelsuma breviceps | 48 | 2 | 681 | 681 | 1.00000 |
| Squamata | Gekkonidae | Phelsuma cepediana | 58 | 2 | 493 | 493 | 1.00000 |
| Squamata | Gekkonidae | Phelsuma dubia | 68 | 10 | 201044 | 238450 | 0.84313 |
| Squamata | Gekkonidae | Phelsuma flavigularis | 66 | 1 | 314 | 314 | 1.00000 |
| Squamata | Gekkonidae | Phelsuma guttata | 52 | 18 | 52327 | 81480 | 0.64221 |
| Squamata | Gekkonidae | Phelsuma hielscheri | 73 | 3 | 51725 | 156055 | 0.33145 |
| Squamata | Gekkonidae | Phelsuma kely | 33 | 1 | 154 | 154 | 1.00000 |
| Squamata | Gekkonidae | Phelsuma klemmeri | 43 | 1 | 289 | 289 | 1.00000 |
| Squamata | Gekkonidae | Phelsuma laticauda | 58 | 20 | 51560 | 88574 | 0.58212 |
| Squamata | Gekkonidae | Phelsuma lineata | 64 | 52 | 184795 | 186249 | 0.99219 |
| Squamata | Gekkonidae | Phelsuma madagascariensis complex | 120 | 56 | 193313 | 363077 | 0.53243 |
| Squamata | Gekkonidae | Phelsuma malamakibo | 61 | 2 | 410 | 410 | 1.00000 |
| Squamata | Gekkonidae | Phelsuma masohoala | 47 | 1 | 131 | 131 | 1.00000 |
| Squamata | Gekkonidae | Phelsuma modesta | 57 | 11 | 77588 | 120492 | 0.64393 |
| Squamata | Gekkonidae | Phelsuma mutabilis | 50 | 15 | 178686 | 197572 | 0.90441 |
| Squamata | Gekkonidae | Phelsuma parva | 36 | 6 | 28862 | 43275 | 0.66695 |
| Squamata | Gekkonidae | Phelsuma pronki | 50 | 1 | 314 | 314 | 1.00000 |
| Squamata | Gekkonidae | Phelsuma pusilla | 42 | 17 | 62889 | 76917 | 0.81763 |
| Squamata | Gekkonidae | Phelsuma quadriocellata | 63 | 36 | 127788 | 133310 | 0.95858 |
| Squamata | Gekkonidae | Phelsuma quadriocellata lepida | 61 | 4 | 66542 | 360040 | 0.18482 |
| Squamata | Gekkonidae | Phelsuma seippi | 55 | 8 | 10777 | 27284 | 0.39499 |
| Squamata | Gekkonidae | Phelsuma serraticauda | 60 | 1 | 349 | 349 | 1.00000 |
| Squamata | Gekkonidae | Phelsuma standingi | 135 | 5 | 43217 | 360040 | 0.12003 |
| Squamata | Gekkonidae | Phelsuma vanheygeni | 35 | 1 | 289 | 289 | 1.00000 |
| Squamata | Gekkonidae | Uroplatus alluaudi | 79 | 3 | 16067 | 67384 | 0.23843 |
| Squamata | Gekkonidae | Uroplatus ebenaui | 66 | 23 | 107233 | 123989 | 0.86486 |
| Squamata | Gekkonidae | Uroplatus fimbriatus | 177 | 41 | 133646 | 133669 | 0.99983 |
| Squamata | Gekkonidae | Uroplatus giganteus | 198 | 4 | 2585 | 19615 | 0.13178 |
| Squamata | Gekkonidae | Uroplatus guentheri | 79 | 14 | 56820 | 78347 | 0.72524 |
| Squamata | Gekkonidae | Uroplatus henkeli | 160 | 11 | 101573 | 124871 | 0.81343 |
| Squamata | Gekkonidae | Uroplatus lineatus | 139 | 17 | 47940 | 79536 | 0.60274 |
| Squamata | Gekkonidae | Uroplatus malahelo | 79 | 11 | 67949 | 144013 | 0.47182 |
| Squamata | Gekkonidae | Uroplatus malama | 71 | 6 | 31429 | 112582 | 0.27917 |
| Squamata | Gekkonidae | Uroplatus phantasticus | 66 | 10 | 92365 | 162772 | 0.56745 |
| Squamata | Gekkonidae | Uroplatus pietschmanni | 81 | 1 | 314 | 314 | 1.00000 |
| Squamata | Gekkonidae | Uroplatus sameiti | 123 | 4 | 9106 | 16355 | 0.55681 |
| Squamata | Gekkonidae | Uroplatus sikorae | 123 | 24 | 165591 | 168909 | 0.98036 |
| Squamata | Gerrhosauridae | Tracheloptychus madagascariensis | 90 | 22 | 58303 | 60675 | 0.96091 |
| Squamata | Gerrhosauridae | Tracheloptychus petersi | 90 | 1 | 512 | 512 | 1.00000 |
| Squamata | Gerrhosauridae | Zonosaurus aeneus | 70 | 25 | 117052 | 145496 | 0.80450 |
| Squamata | Gerrhosauridae | Zonosaurus anelanelany | 86 | 5 | 12241 | 91971 | 0.13310 |
| Squamata | Gerrhosauridae | Zonosaurus bemaraha | 75 | 2 | 320 | 320 | 1.00000 |
| Squamata | Gerrhosauridae | Zonosaurus boettgeri | 120 | 9 | 30541 | 57243 | 0.53354 |
| Squamata | Gerrhosauridae | Zonosaurus brygooi | 76 | 8 | 30377 | 59145 | 0.51360 |
| Squamata | Gerrhosauridae | Zonosaurus haraldmeieri | 140 | 3 | 3080 | 42156 | 0.07306 |
| Squamata | Gerrhosauridae | Zonosaurus karsteni | 133 | 6 | 218203 | 278205 | 0.78432 |
| Squamata | Gerrhosauridae | Zonosaurus laticaudatus | 135 | 24 | 155048 | 172593 | 0.89835 |
| Squamata | Gerrhosauridae | Zonosaurus madagascariensis | 127 | 53 | 187206 | 189930 | 0.98566 |
| Squamata | Gerrhosauridae | Zonosaurus maramaitso | 120 | 1 | 314 | 314 | 1.00000 |
| Squamata | Gerrhosauridae | Zonosaurus maximus | 246 | 9 | 70056 | 189318 | 0.37004 |
| Squamata | Gerrhosauridae | Zonosaurus ornatus | 132 | 23 | 118774 | 144655 | 0.82109 |
| Squamata | Gerrhosauridae | Zonosaurus quadrilineatus | 165 | 1 | 365 | 365 | 1.00000 |
| Squamata | Gerrhosauridae | Zonosaurus rufipes | 88 | 7 | 34264 | 158031 | 0.21682 |
| Squamata | Gerrhosauridae | Zonosaurus subunicolor | 86 | 8 | 32720 | 136159 | 0.24031 |
| Squamata | Gerrhosauridae | Zonosaurus trilineatus | 152 | 11 | 35741 | 39202 | 0.91170 |
| Squamata | Gerrhosauridae | Zonosaurus tsingy | 85 | 3 | 2655 | 18880 | 0.14062 |
| Squamata | Iguanidae | Chalarodon madagascariensis | 87 | 25 | 184090 | 196651 | 0.93613 |
| Squamata | Iguanidae | Oplurus cuvieri | 126 | 31 | 202541 | 283948 | 0.71330 |
| Squamata | Iguanidae | Oplurus cyclurus | NA | 32 | 179956 | 214916 | 0.83733 |
| Squamata | Iguanidae | Oplurus fierinensis | 98 | 1 | 336 | 336 | 1.00000 |
| Squamata | Iguanidae | Oplurus grandidieri | 110 | 9 | 76053 | 206529 | 0.36824 |
| Squamata | Iguanidae | Oplurus quadrimaculatus | 123 | 33 | 123346 | 140468 | 0.87810 |
| Squamata | Iguanidae | Oplurus saxicola | 109 | 16 | 94331 | 98594 | 0.95677 |
| Squamata | Lamprophiidae | Alluaudina bellyi | 269 | 10 | 206465 | 256614 | 0.80457 |
| Squamata | Lamprophiidae | Alluaudina mocquardi | 330 | 1 | 243 | 243 | 1.00000 |
| Squamata | Lamprophiidae | Brygophis coulangesi | 800 | 2 | 628 | 628 | 1.00000 |
| Squamata | Lamprophiidae | Compsophis albiventris | 430 | 1 | 314 | 314 | 1.00000 |
| Squamata | Lamprophiidae | Compsophis boulengeri | 298 | 11 | 111693 | 143064 | 0.78072 |
| Squamata | Lamprophiidae | Compsophis fatsibe | 410 | 2 | 628 | 628 | 1.00000 |
| Squamata | Lamprophiidae | Compsophis infralineatus | 635 | 13 | 125021 | 159814 | 0.78229 |
| Squamata | Lamprophiidae | Compsophis laphystius | 410 | 14 | 100809 | 110534 | 0.91202 |
| Squamata | Lamprophiidae | Compsophis vinckei | 411 | 2 | 423 | 423 | 1.00000 |
| Squamata | Lamprophiidae | Compsophis zeny | 190 | 2 | 628 | 628 | 1.00000 |
| Squamata | Lamprophiidae | Dromicodryas bernieri | 740 | 31 | 324088 | 335297 | 0.96657 |
| Squamata | Lamprophiidae | Dromicodryas quadrilineatus | 800 | 25 | 233575 | 248193 | 0.94110 |
| Squamata | Lamprophiidae | Exallodontophis albignaci | 280 | 5 | 116001 | 222560 | 0.52121 |
| Squamata | Lamprophiidae | Heteroliodon fohy | 190 | 1 | 220 | 220 | 1.00000 |
| Squamata | Lamprophiidae | Heteroliodon lava | 365 | 2 | 628 | 628 | 1.00000 |
| Squamata | Lamprophiidae | Heteroliodon occipitalis | 263 | 9 | 164231 | 215480 | 0.76216 |
| Squamata | Lamprophiidae | Ithycyphus blanci | 400 | 1 | 314 | 314 | 1.00000 |
| Squamata | Lamprophiidae | Ithycyphus goudoti | 580 | 9 | 47778 | 53071 | 0.90027 |
| Squamata | Lamprophiidae | Ithycyphus miniatus | 1100 | 15 | 101088 | 132129 | 0.76507 |
| Squamata | Lamprophiidae | Ithycyphus oursi | 1050 | 19 | 101902 | 104572 | 0.97446 |
| Squamata | Lamprophiidae | Ithycyphus perineti | 1000 | 12 | 184466 | 271688 | 0.67896 |
| Squamata | Lamprophiidae | Langaha alluaudi | 730 | 8 | 127480 | 190880 | 0.66785 |
| Squamata | Lamprophiidae | Langaha madagascariensis | 660 | 20 | 243843 | 290016 | 0.84079 |
| Squamata | Lamprophiidae | Langaha pseudoalluaudi | 860 | 4 | 129382 | 183660 | 0.70446 |
| Squamata | Lamprophiidae | Leioheterodon geayi | 930 | 7 | 104472 | 115695 | 0.90300 |
| Squamata | Lamprophiidae | Leioheterodon madagascariensis | 1000 | 34 | 331540 | 348416 | 0.95156 |
| Squamata | Lamprophiidae | Leioheterodon modestus | 800 | 16 | 259080 | 335097 | 0.77315 |
| Squamata | Lamprophiidae | Liophidium apperti | 160 | 2 | 628 | 628 | 1.00000 |
| Squamata | Lamprophiidae | Liophidium chabaudi | 330 |  | 368 | 368 | 1.00000 |
| Squamata | Lamprophiidae | Liophidium maintikibo | 205 | 1 | 314 | 314 | 1.00000 |
| Squamata | Lamprophiidae | Liophidium pattoni | 329 | 1 | 314 | 314 | 1.00000 |
| Squamata | Lamprophiidae | Liophidium rhodogaster | 400 | 15 | 161547 | 165576 | 0.97567 |
| Squamata | Lamprophiidae | Liophidium sp aff rhodoscelis | 205 | 1 | 314 | 314 | 1.00000 |
| Squamata | Lamprophiidae | Liophidium therezieni | 610 | 1 | 314 | 314 | 1.00000 |
| Squamata | Lamprophiidae | Liophidium torquatum | 470 | 33 | 197764 | 222869 | 0.88736 |
| Squamata | Lamprophiidae | Liophidium trilineatum | 220 | 2 | 566 | 566 | 1.00000 |
| Squamata | Lamprophiidae | Liophidium vaillanti | 410 | 11 | 199846 | 243727 | 0.81996 |
| Squamata | Lamprophiidae | Liopholidophis dimorphus | 615 | 1 | 314 | 314 | 1.00000 |
| Squamata | Lamprophiidae | Liopholidophis dolicocercus | 770 | 7 | 122132 | 165987 | 0.73579 |
| Squamata | Lamprophiidae | Liopholidophis grandidieri | 1636 | 2 | 628 | 628 | 1.00000 |
| Squamata | Lamprophiidae | Liopholidophis rhadinaea | 429 | 6 | 182391 | 235967 | 0.77295 |
| Squamata | Lamprophiidae | Liopholidophis sexlineatus | 650 | 14 | 152454 | 156702 | 0.97289 |
| Squamata | Lamprophiidae | Liopholidophis varius | 359 | 5 | 39325 | 212442 | 0.18511 |
| Squamata | Lamprophiidae | Lycodryas carleti | 605 | 3 | 38543 | 223130 | 0.17274 |
| Squamata | Lamprophiidae | Lycodryas citrinus | 535 | 4 | 30837 | 139139 | 0.22163 |
| Squamata | Lamprophiidae | Lycodryas gaimardi | 675 | 6 | 78451 | 199988 | 0.39228 |
| Squamata | Lamprophiidae | Lycodryas granuliceps | 892 | 8 | 16119 | 20152 | 0.79988 |
| Squamata | Lamprophiidae | Lycodryas guentheri | 420 | 3 | 58387 | 211839 | 0.27562 |
| Squamata | Lamprophiidae | Lycodryas inopinae | 478 | 4 | 5895 | 18831 | 0.31303 |
| Squamata | Lamprophiidae | Lycodryas inornatus | 470 | 1 | 314 | 314 | 1.00000 |
| Squamata | Lamprophiidae | Lycodryas pseudogranuliceps | 564 | 8 | 299933 | 348624 | 0.86033 |
| Squamata | Lamprophiidae | Madagascarophis colubrinus | 700 | 39 | 305605 | 307801 | 0.99286 |
| Squamata | Lamprophiidae | Madagascarophis meridionalis | 580 | 4 | 143961 | 298616 | 0.48209 |
| Squamata | Lamprophiidae | Madagascarophis ocellatus | 550 | 5 | 31392 | 36826 | 0.85244 |
| Squamata | Lamprophiidae | Micropisthodon ochraceus | 470 | 9 | 190041 | 250592 | 0.75837 |
| Squamata | Lamprophiidae | Mimophis mahfalensis | 670 | 32 | 260435 | 363077 | 0.71730 |
| Squamata | Lamprophiidae | Pararhadinaea melanogaster | 247 | 1 | 228 | 228 | 1.00000 |
| Squamata | Lamprophiidae | Parastenophis betsileanus | 985 | 13 | 211833 | 245330 | 0.86346 |
| Squamata | Lamprophiidae | Phisalixella arctifasciata | 622 | 7 | 223266 | 297293 | 0.75099 |
| Squamata | Lamprophiidae | Phisalixella tulearensis | 1000 | 3 | 96063 | 109848 | 0.87450 |
| Squamata | Lamprophiidae | Phisalixella variabilis | 1000 | 9 | 367452 | 388563 | 0.94567 |
| Squamata | Lamprophiidae | Pseudoxyrhopus ambreensis | 333 | 2 | 533 | 533 | 1.00000 |
| Squamata | Lamprophiidae | Pseudoxyrhopus analabe | 260 | 2 | 628 | 628 | 1.00000 |
| Squamata | Lamprophiidae | Pseudoxyrhopus ankafinaensis | 945 | 1 | 314 | 314 | 1.00000 |
| Squamata | Lamprophiidae | Pseudoxyrhopus heterurus | 510 | 8 | 113736 | 158704 | 0.71665 |
| Squamata | Lamprophiidae | Pseudoxyrhopus imerinae | 362 | 5 | 48462 | 61623 | 0.78643 |
| Squamata | Lamprophiidae | Pseudoxyrhopus kely | 180 | 3 | 7125 | 64418 | 0.11061 |
| Squamata | Lamprophiidae | Pseudoxyrhopus microps | 1195 | 18 | 129513 | 129804 | 0.99775 |
| Squamata | Lamprophiidae | Pseudoxyrhopus oblectator | 329 | 3 | 2692 | 116029 | 0.02320 |
| Squamata | Lamprophiidae | Pseudoxyrhopus quinquelineatus | 460 | 14 | 369700 | 396214 | 0.93308 |
| Squamata | Lamprophiidae | Pseudoxyrhopus sokosoko | 285 | 6 | 6846 | 73526 | 0.09311 |
| Squamata | Lamprophiidae | Pseudoxyrhopus tritaeniatus | 850 | 14 | 124757 | 156609 | 0.79662 |
| Squamata | Lamprophiidae | Thamnosophis epistibes | 514 | 17 | 66658 | 69036 | 0.96556 |
| Squamata | Lamprophiidae | Thamnosophis infrasignatus | 576 | 19 | 52500 | 52697 | 0.99626 |
| Squamata | Lamprophiidae | Thamnosophis lateralis | 517 | 50 | 258275 | 267118 | 0.96689 |
| Squamata | Lamprophiidae | Thamnosophis martae | 598 | 2 | 622 | 622 | 1.00000 |
| Squamata | Lamprophiidae | Thamnosophis stumpffi | 482 | 7 | 27265 | 112324 | 0.24273 |
| Squamata | Scincidae | Amphiglossus alluaudi | 85 | 3 | 3486 | 87899 | 0.03966 |
| Squamata | Scincidae | Amphiglossus andranovahensis | 38 | 1 | 314 | 314 | 1.00000 |
| Squamata | Scincidae | Amphiglossus anosyensis | 58 | 10 | 34548 | 69404 | 0.49779 |
| Squamata | Scincidae | Amphiglossus ardouini | 132 | 3 | 3268 | 10697 | 0.30551 |
| Squamata | Scincidae | Amphiglossus astrolabi | 226 | 13 | 165643 | 183686 | 0.90177 |
| Squamata | Scincidae | Amphiglossus crenni | 85 | 7 | 100687 | 262828 | 0.38309 |
| Squamata | Scincidae | Amphiglossus elongatus | 100 | 1 | 314 | 314 | 1.00000 |
| Squamata | Scincidae | Amphiglossus frontoparietalis | 76 | 15 | 120360 | 128041 | 0.94001 |
| Squamata | Scincidae | Amphiglossus gastrostictus | 104 | 2 | 628 | 628 | 1.00000 |
| Squamata | Scincidae | Amphiglossus macrocercus | 110 | 7 | 53401 | 77422 | 0.68973 |
| Squamata | Scincidae | Amphiglossus mandady | 62 | 1 | 314 | 314 | 1.00000 |
| Squamata | Scincidae | Amphiglossus mandokava | 148 | 3 | 53153 | 220253 | 0.24133 |
| Squamata | Scincidae | Amphiglossus melanurus | 59 | 5 | 27572 | 188517 | 0.14626 |
| Squamata | Scincidae | Amphiglossus ornaticeps | 62 | 12 | 231460 | 239206 | 0.96762 |
| Squamata | Scincidae | Amphiglossus punctatus | 73 | 18 | 87638 | 92786 | 0.94452 |
| Squamata | Scincidae | Amphiglossus reticulatus | 212 | 7 | 117024 | 149076 | 0.78499 |
| Squamata | Scincidae | Amphiglossus sp phaeurus | 84 | 6 | 46459 |  |  |
| Squamata | Scincidae | Amphiglossus spilostichus | 81 | 1 | 314 | 314 | 1.00000 |
| Squamata | Scincidae | Amphiglossus splendidus | 106 | 10 | 26088 | 69212 | 0.37693 |
| Squamata | Scincidae | Amphiglossus stylus | 65 | 1 | 314 | 314 | 1.00000 |
| Squamata | Scincidae | Amphiglossus tanysoma | NA | 3 | 30698 | 76690 | 0.40029 |
| Squamata | Scincidae | Amphiglossus tsaratananensis | 69 | 3 | 1807 | 20816 | 0.08683 |
| Squamata | Scincidae | Androngo trivittatus | 147 | 14 | 41195 | 49153 | 0.83811 |
| Squamata | Scincidae | Cryptoblepharus boutonii cognatus | 52 | 4 | 308 | 6704 | 0.04593 |
| Squamata | Scincidae | Cryptoblepharus boutonii voelt | 52 | 1 | 552 | 552 | 1.00000 |
| Squamata | Scincidae | Cryptoblepharus boutonii voeltzkowi | 52 | 1 | 552 | 552 | 1.00000 |
| Squamata | Scincidae | Madascincus ankodabensis | 34 | 2 | 488 | 488 | 1.00000 |
| Squamata | Scincidae | Madascincus arenicola | 73 | 2 | 274 | 274 | 1.00000 |
| Squamata | Scincidae | Madascincus igneocaudatus | 78 | 10 | 31060 | 31068 | 0.99977 |
| Squamata | Scincidae | Madascincus macrolepis | 34 | 1 | 314 | 314 | 1.00000 |
| Squamata | Scincidae | Madascincus melanopleura | 55 | 56 | 123708 | 123960 | 0.99796 |
| Squamata | Scincidae | Madascincus mouroundavae | 66 | 12 | 148105 | 205158 | 0.72191 |
| Squamata | Scincidae | Madascincus nanus | 29 | 4 | 10863 | 148990 | 0.07291 |
| Squamata | Scincidae | Madascincus polleni | NA | 6 | 117455 | 166056 | 0.70733 |
| Squamata | Scincidae | Madascincus sp aff igneocaudatus Highlands | 78 | 2 | 629 | 629 | 1.00000 |
| Squamata | Scincidae | Madascincus sp aff polleni North | NA | 5 | 6860 | 24445 | 0.28065 |
| Squamata | Scincidae | Madascincus sp baeus | 29 | 1 | 314 | 314 | 1.00000 |
| Squamata | Scincidae | Madascincus stumpffi | NA | 10 | 51603 | 120054 | 0.42983 |
| Squamata | Scincidae | Paracontias brocchii | 97 | 2 | 533 | 533 | 1.00000 |
| Squamata | Scincidae | Paracontias fasika | 34 | 1 | 99 | 99 | 1.00000 |
| Squamata | Scincidae | Paracontias hafa | 69 | 1 | 314 | 314 | 1.00000 |
| Squamata | Scincidae | Paracontias hildebrandti | 46 | 6 | 17335 | 20522 | 0.84474 |
| Squamata | Scincidae | Paracontias holomelas | 158 | 3 | 87473 | 171517 | 0.51000 |
| Squamata | Scincidae | Paracontias kankana | 59 | 1 | 314 | 314 | 1.00000 |
| Squamata | Scincidae | Paracontias manify | 67 | 1 | 314 | 314 | 1.00000 |
| Squamata | Scincidae | Paracontias milloti | 42 | 2 | 449 | 449 | 1.00000 |
| Squamata | Scincidae | Paracontias minimus | 75 | 1 | 99 | 99 | 1.00000 |
| Squamata | Scincidae | Paracontias rothschildi | 52 |  | 99 | 99 | 1.00000 |
| Squamata | Scincidae | Paracontias sp vermisaurus | 54 | 1 | 314 | 314 | 1.00000 |
| Squamata | Scincidae | Paracontias tsararano | 66 | 1 | 314 | 314 | 1.00000 |
| Squamata | Scincidae | Pseudoacontias angelorum | 207 | 2 | 343 | 343 | 1.00000 |
| Squamata | Scincidae | Pseudoacontias menamainty | 224 | 1 | 254 | 254 | 1.00000 |
| Squamata | Scincidae | Pseudoacontias unicolor | 200 | 1 | 133 | 133 | 1.00000 |
| Squamata | Scincidae | Pygomeles braconnieri | 162 | 2 | 7871 | 10203 | 0.77148 |
| Squamata | Scincidae | Pygomeles petteri | 162 | 2 | 628 | 628 | 1.00000 |
| Squamata | Scincidae | Sirenoscincus yamagishii | 87 | 1 | 314 | 314 | 1.00000 |
| Squamata | Scincidae | Trachylepis aureopunctatus | 82 | 16 | 132470 | 142627 | 0.92879 |
| Squamata | Scincidae | Trachylepis boettgeri | 55 | 14 | 60028 | 89369 | 0.67169 |
| Squamata | Scincidae | Trachylepis dumasi | 55 | 4 | 107283 | 227116 | 0.47237 |
| Squamata | Scincidae | Trachylepis elegans | 59 | 63 | 304131 | 305523 | 0.99544 |
| Squamata | Scincidae | Trachylepis gravenhorstii | 94 | 85 | 295259 | 300007 | 0.98417 |
| Squamata | Scincidae | Trachylepis lavarambo | 61 | 1 | 107 | 107 | 1.00000 |
| Squamata | Scincidae | Trachylepis madagascariensis | 69 | 11 | 54391 | 74765 | 0.72749 |
| Squamata | Scincidae | Trachylepis nancycoutuae | 45 | 1 | 314 | 314 | 1.00000 |
| Squamata | Scincidae | Trachylepis tandrefana | 58 | 7 | 56778 | 129880 | 0.43716 |
| Squamata | Scincidae | Trachylepis tavaratra | 62 | 5 | 11641 | 33567 | 0.34678 |
| Squamata | Scincidae | Trachylepis vato | 55 | 13 | 155861 | 184979 | 0.84259 |
| Squamata | Scincidae | Trachylepis vezo | 54 | 1 | 271 | 271 | 1.00000 |
| Squamata | Scincidae | Trachylepis volamenaloha | 52 | 3 | 1978 | 20791 | 0.09515 |
| Squamata | Scincidae | Voeltzkowia fierinensis | 72 | 6 | 26211 | 45015 | 0.58227 |
| Squamata | Scincidae | Voeltzkowia lineata | 79 | 10 | 49853 | 51812 | 0.96219 |
| Squamata | Scincidae | Voeltzkowia mira | 80 | 2 | 6856 | 8496 | 0.80693 |
| Squamata | Scincidae | Voeltzkowia petiti | 56 |  | 194 | 194 | 1.00000 |
| Squamata | Scincidae | Voeltzkowia rubrocaudata | 89 | 7 | 92693 | 167006 | 0.55503 |
| Squamata | Scincidae | Voeltzkowia sp. pallida | 93 | 2 | 344 | 344 | 1.00000 |
| Squamata | Typhlopidae | Ramphotyphlops braminus | 175 | 13 | 113713 | 186368 | 0.61015 |
| Squamata | Typhlopidae | Typhlops arenarius | 220 | 5 | 179113 | 325012 | 0.55110 |
| Squamata | Typhlopidae | Typhlops decorsei | 600 | 4 | 289264 | 328393 | 0.88085 |
| Squamata | Typhlopidae | Typhlops domerguei | 150 | 1 | 314 | 314 | 1.00000 |
| Squamata | Typhlopidae | Typhlops madagascariensis | 410 | 1 | 228 | 228 | 1.00000 |
| Squamata | Typhlopidae | Typhlops microcephalus | 235 | 4 | 5402 | 21701 | 0.24895 |
| Squamata | Typhlopidae | Typhlops mucronatus | 378 | 7 | 58611 | 209993 | 0.27911 |
| Squamata | Typhlopidae | Typhlops ocularis | 342 | 3 | 4021 | 30345 | 0.13250 |
| Squamata | Typhlopidae | Typhlops reuteri | 95 | 2 | 542 | 542 | 1.00000 |
| Squamata | Xenotyphlopidae | Xenotyphlops grandidieri | 276 | 1 | 312 | 312 | 1.00000 |
| Testudines | Pelomedusidae | Pelomedusa subrufa | 250 | 9 | 240249 | 264436 | 0.90853 |
| Testudines | Pelomedusidae | Pelusios castanoides | 230 | 10 | 232866 | 347415 | 0.67028 |
| Testudines | Pelomedusidae | Pelusios subniger | 195 | 3 | 3274 | 3421 | 0.95725 |
| Testudines | Podocnemididae | Erymnochelys madagascariensis | 410 | 12 | 96683 | 124276 | 0.77797 |
| Testudines | Testudinidae | Astrochelys radiata | 400 | 27 | 18802 | 19048 | 0.98708 |
| Testudines | Testudinidae | Astrochelys yniphora | 446 | 2 | 382 | 382 | 1.00000 |
| Testudines | Testudinidae | Pyxis arachnoides | 116 | 26 | 17206 | 20627 | 0.83413 |
| Testudines | Testudinidae | Pyxis planicauda | 137 | 2 | 628 | 628 | 1.00000 |
