# Supplementary material for: Spatial Biodiversity Patterns of Madagascar's Amphibians and Reptiles
Source: PLoS One. 2016 Jan 6;11(1):e0144076. doi: 10.1371/journal.pone.0144076 (PMC4703303; doi:10.1371/journal.pone.0144076)
Supplement: S2 Table — (DOC) [file pone.0144076.s003.doc]

**Spatial biodiversity patterns of Madagascar's amphibians and reptiles**

Jason Brown, Neftali Sillero, Frank Glaw, Parfait Bora, David R. Vieites, Miguel Vences

**Supplementary Materials**

**S2 Table.** Summary of survey data used in the meta-analysis and references for the original data.

| **Site** | **Reference** |
| --- | --- |
| Andohahela s1 | Nussbaum et al. 1999 |
| Andohahela s2 | Nussbaum et al. 1999 |
| Andohahela s3 | Nussbaum et al. 1999 |
| Andohahela s4 | Nussbaum et al. 1999 |
| Andohahela s5 | Nussbaum et al. 1999 |
| Andohahela s6 | Nussbaum et al. 1999 |
| Manongarivo s1 | Rakotomalala 2002 |
| Manongarivo s2 | Rakotomalala 2002 |
| Manongarivo s3 | Rakotomalala 2002 |
| Manongarivo s4 | Rakotomalala 2002 |
| Zombitse s1 | Raxworthy et al. 1994 |
| Zombitse s2 | Raxworthy et al. 1994 |
| Zombitse s3 | Raxworthy et al. 1994 |
| Zombitse s4 | Raxworthy et al. 1994 |
| PN Tsimanampetsotsa r1 |  |
| PN Andohahela Parcelle2 r2 |  |
| Mikea r3 |  |
| Anjanaharibe Sud versant ouest s1 | Rakotomalala & Raselimanana 2003 |
| Anjanaharibe Sud versant ouest s2 | Rakotomalala & Raselimanana 2003 |
| Foret Betaolana site3 | Rakotomalala & Raselimanana 2003 |
| Foret Betaolana site4 | Rakotomalala & Raselimanana 2003 |
| Marojejy versant nord ouest s5 | Rakotomalala & Raselimanana 2003 |
| Marojejy versant nord ouest s6 | Rakotomalala & Raselimanana 2003 |
| Vohibasia s1 | Goodman et al. 1997 |
| Vohimena s1 | Goodman et al. 1997 |
| Tampolo s1 | Raselimanana et al. 1998 |
| Anjanaharibe sud e1 | Raxworthy et al. 1998 |
| Anjanaharibe sud e2 | Raxworthy et al. 1998 |
| Anjanaharibe sud e3 | Raxworthy et al. 1998 |
| Anjanaharibe sud e4 | Raxworthy et al. 1998 |
| Anjanaharibe sud w1 | Raxworthy et al. 1998 |
| Anjanaharibe sud w2 | Raxworthy et al. 1998 |
| HSDED Marojejy s1 t1 450 | Raselimanana et al. 2000 |
| HSDED Marojejy s2 t2 750 | Raselimanana et al. 2000 |
| HSDED Marojejy s3 t3 1225 | Raselimanana et al. 2000 |
| HSDED Marojejy s4 t4 1625 | Raselimanana et al. 2000 |
| HSDED Marojejy s5 t5 1875 | Raselimanana et al. 2000 |
| HSDED Marojejy s 80 | Raselimanana et al. 2000 |
| HSDED Marojejy s Mandena 70-100 | Raselimanana et al. 2000 |
| HSDED Marojejy s 800 close Andapa | Raselimanana et al. 2000 |
| RNI now PN Andringitra s1 c1 720 | Raxworthy & Nussbaum 1996b |
| RNI now PN Andringitra s2 c2 810 | Raxworthy & Nussbaum 1996b |
| RNI now PN Andringitra s3 c3 1210 | Raxworthy & Nussbaum 1996b |
| RNI now PN Andringitra s c4 1625 | Raxworthy & Nussbaum 1996b |
| RNI now PN Andringitra s5 c5 2075 | Raxworthy & Nussbaum 1996b |
| RS Pic Ivohibe s1 900 | Raselimanana 1999 |
| RS Pic Ivohibe s2 1200 | Raselimanana 1999 |
| RS Pic Ivohibe s3 1575 | Raselimanana 1999 |
| Corridor s1 1200 | Raselimanana 1999 |
| Corridor s2 900 | Raselimanana 1999 |
| Andranomay Anjozorobe s1 | Raselimanana 1998 |
| Befotaka Midongy s1 Rozabe | Bora et al. 2007 |
| Befotaka Midongy s2 Kilimagnarivo | Bora et al. 2007 |
| Tsingy de Bemaraha s1 | Bora et al. 2010 |
| Tsingy de Bemaraha s2 | Bora et al. 2010 |
| Tsingy de Bemaraha s3 | Bora et al. 2010 |
| Tsingy de Bemaraha s4 | Bora et al. 2010 |
| Tsingy de Bemaraha s5 | Bora et al. 2010 |
| Tsingy de Bemaraha s6 | Bora et al. 2010 |
| Tsingy de Bemaraha s7 | Bora et al. 2010 |
| Tsingy de Bemaraha s8 | Bora et al. 2010 |
| Tsingy de Bemaraha s9 | Bora et al. 2010 |
| Tsingy de Bemaraha s10 | Bora et al. 2010 |
| Nosy Be RNI Lokobe a1 | Andreone et al. 2003 |
| Nosy Be RNI Lokobe a2 | Andreone et al. 2003 |
| Tsaratanana s1 Andampy | Andreone et al. 2009 |
| Tsaratanana s2 Antsahamanara | Andreone et al. 2009 |
| Tsaratanana s3 Camp-Norbert | Andreone et al. 2009 |
| Tsaratanana s4 Camp-0 | Andreone et al. 2009 |
| Tsaratanana s5 Camp-1 | Andreone et al. 2009 |
| Montagne des Francais / Andavakoera w1 | D'Cruze et al. 2007 |
| Montagne des Francais / Andavakoera d1 | D'Cruze et al. 2007 |
| Montagne des Francais / Andavakoera d2 | D'Cruze et al. 2007 |
| Montagne des Francais / Andavakoera w2 | D'Cruze et al. 2007 |
| Central high plateau s1 Soamazaka | Andreone et al. 2007 |
| Central high plateau s2 Vohitsokina | Andreone et al. 2007 |
| Central high plateau s3 Farihimazava | Andreone et al. 2007 |
| Central high plateau s4 Vatolampy | Andreone et al. 2007 |
| Central high plateau s5 Antratrabe 1 | Andreone et al. 2007 |
| Central high plateau s6 Antratrabe 2 | Andreone et al. 2007 |
| Central high plateau s7 Ambatodradama | Andreone et al. 2007 |
| Central high plateau s8 Itremo | Andreone et al. 2007 |
| Central high plateau s9 Andrangoloaka | Andreone et al. 2007 |
| Ankarafantsika s1 | Ramanamanjato & Rabibisoa 2002 |
| Ankarafantsika s2 | Ramanamanjato & Rabibisoa 2002 |
| Ankarafantsika s3 | Ramanamanjato & Rabibisoa 2002 |
| Corridor Andringitra-Ranomafana s1 Ambatambe | Rakotomalala et al. 2001. |
| Corridor Andringitra-Ranomafana s2 Ankopakopaka | Rakotomalala et al. 2001. |
| Corridor Andringitra-Ranomafana s3 Mandriandry | Rakotomalala et al. 2001. |
| Corridor Andringitra-Ranomafana s4 Ambahaka | Rakotomalala et al. 2001. |
| Corridor Andringitra-Ranomafana s5 Andrambovato | Rakotomalala et al. 2001. |
| Corridor Andringitra-Ranomafana s6 Vinanitelo | Rakotomalala et al. 2001. |
| Corridor Andringitra-Ranomafana s7 Manambolo 1 | Rakotomalala et al. 2001. |
| Corridor Andringitra-Ranomafana s8 Manambolo 2 | Rakotomalala et al. 2001. |
| PN Ranomafana s9 | Rakotomalala et al. 2001. |
| PN Ranomafana s10 | Rakotomalala et al. 2001. |
| PN Ranomafana s11 | Rakotomalala et al. 2001. |
| Mikea s1 Ankazomafio | Raselimanana 2004 |
| Mikea s2 Abrahama Jiloriaky | Raselimanana 2004 |
| Mikea s3 Andalandomo | Raselimanana 2004 |
| Mikea s4 Ankindranoky | Raselimanana 2004 |
| Mikea s5 Ankotapiky | Raselimanana 2004 |
| Mikea s6 Maharihy | Raselimanana 2004 |

Andreone. F.. F. Glaw. F. Mattioli. R. Jesu. G. Schimmenti. J. E. Randrianirina & M. Vences (2009): The peculiar herpetofauna of some Tsaratanana rainforests and its affinities with Manongarivo and other massifs and forests of northern Madagascar.- Italian Journal of Zoology 76: 92-110

Andreone. F.. F. Glaw. R. A. Nussbaum. C. J. Raxworthy. M. Vences & J. E. Randrianirina (2003): The amphibians and reptiles of Nosy Be (NW Madagascar) and nearby islands: a case study of diversity and conservation of an insular fauna. – Journal of Natural History 37 (17): 2119-2149.

Andreone. F.. M. Vences. F. Glaw & J. E. Randrianirina (2007): Remarkable records of amphibians and reptiles on Madagascar’s central high plateau.- Tropical Zoology 20 (1): 19-39

Bora. P.. J. C. Randrianantoandro. R. Randrianavelona. E. F. Hantalalaina. R. R. Andriantsimanarilafy. D. Rakotondravony. O. R. Ramilijaona. M. Vences. R. K. B. Jenkins. F. Glaw & J. Köhler (2010): Amphibians and reptiles of the Tsingy de Bemaraha plateau. western Madagascar: checklist. biogeography and conservation.- Herpetological Conservation and Biology 5 (1): 111-125

Bora. P.. M. O. Randriambahiniarime. F. C. Rabemananjara. O. Ravoahangimalala Ramilijaona. F. Glaw & M. Vences (2007): A rapid assessment survey of the herpetofauna at Befotaka-Midongy National Park. south-eastern Madagascar.- Mitteilungen aus dem Museum für Naturkunde in Berlin. Zoologische Reihe 83 (2): 170-178

Goodman. S. M.. J.-B. Ramanamanjato & A. Raselimanana (1997): Chapitre 7. Les Amphibiens et les Reptiles. pp. 110-130. In: Inventaire Biologique Foret de Vohibasia et d'Isoky-Vohimena (O. Langrand & S. M. Goodman. eds.). – Recherches pour le developpement Série Sciences biologiques 12. Antananarivo. 197 pp.

D'Cruze. N.. J. Sabel. K. Green. J. Dawson. C. Gardner. J. Robinson. G. Starkie. M. Vences & F. Glaw (2007): The first comprehensive survey of amphibians and reptiles at Montagne des Français. Madagascar.- Herpetological Conservation and Biology 2 (2): 87-99.

Nussbaum. R. A.. C. J. Raxworthy. A. P. Raselimanana & J. B. Ramanamanjato (1999): Amphibians and reptiles of the Réserve Naturelle Integrale d’Andohahela. Madagascar. – Fieldiana Zoology (new series) 94: 155-173.

Rakotomalala. D. (2002): Chapitre 10. Diversité des reptiles et amphibiens de la Réserve Spéciale de Manongarivo. Madagascar. In: Gautier. L. & S. M. Goodman (eds.): Inventaire floristique et faunistique de la Réserve Spéciale de Manongarivo (NW Madagascar). – Boissera 59: 339-358.

Rakotomalala. D.. E. Raholimavo. P. Talata & E. Rajeriarison (2001): Chapitre 7. Les amphibiens et les reptiles du Parc National de Ranomafana et de la zone forestière le reliant au Parc National d'Andringitra. pp. -133-163 in Goodman. S. M. & V. R. Razafindratsita (eds.): Inventaire biologique du Parc National de Ranomafana et du couloir forestier qui la relie au Parc National d'Andringitra. – Recherches pour le developpement. série Sciences Biologiques 17: 1-243.

Rakotomalala. D. & A. Raselimanana (2003): Les amphibiens et des reptiles des massifs de Marojejy. d'Anjanaharibe-Sud et du couloir forestier de Betaolana. pp. 147-202. In: Nouveaux résultats d'inventaires biologiques faisant référence à l'altitude dans la région des massifs montagneux de Marojejy et d'Anjanaharibe-Sud (S. M. Goodman & L. Wilmé. eds.). – Recherches pour le developpement Série Sciences biologiques 19. Antananarivo. 302 pp.

Ramanamanjato. J. B. & N. Rabibisoa (2002): Evaluation rapide de la diversité biologique des reptiles et amphibiens de la Réserve Naturelle Intégrale d'Ankarafantsika. pp. 98-103 and 135-138. In: Alonso. L. E.. T. S. Schulenberg. S. Radilofe & O. Missa (eds): Une evaluation biologique de la Réserve Naturelle Intégrale d'Ankarafantsika. Madagascar. – Bulletin RAP d'evaluation rapide 23. Conservational International. Washington. DC.

Raselimanana. A. P. (1998): La diversité de la faune de reptiles et amphibiens. pp.43-49. In: Inventaire biologique Foret d'Andranomay Anjozorobe (D. Rakotondravony & S. Goodman eds.). – Recherches pour le developpement. série Sciences Biologiques 13: 1-110.

Raselimanana. A. P. (2004): L’herpétofaune de la forêt de Mikea. Dans Inventaire floristique et faunistique de la forêt de Mikea : Paysage écologique et diversité biologique d’une préoccupation majeure pour la conservation. eds. A. P. Raselimanana & S. M. Goodman. Recherches pour le Développement. Séries Sciences biologiques. 21: 37-52.

Raselimanana. A. P. (1999): L'Herpetofaune. pp. 81-97. In: Inventaire biologique de la réserve spéciale du pic d'Ivohibe et du couloir forestier qui la relie au Parc national d'Andringitra (S. Goodman and B. P. N. Rasolonandrasana. eds.). – Recherches pour le developpement. série Sciences Biologiques 15:1-181.

Raselimanana. A. P.. D. Rakotomalala & F. Rakotondraparany (1998): IX - Les reptiles et amphibiens: diversité et conservations. pp. 183-195. In: Inventaire biologique de la foret littorale de Tampolo (Fenoarivo Atsinanana) (J. Ratsirarson & S. Goodman eds.) Recherches pour le developpement. série Sciences Biologiques 14: 1-261.

Raselimanana. A. P.. C. J. Raxworthy & R. A. Nussbaum (2000): Herpetofaunal species diversity and elevational distribution within the Parc National de Marojejy. Madagascar. In: A floral and faunal inventory of the Parc National de Marojejy: with reference to elevational variation (S. M. Goodman ed.). Fieldiana Zoology (new series) 97: 157-174.

Raxworthy. C. J.. F. Andreone. R. A. Nussbaum. N. Rabibisoa & H. Randriamahazo (1998): Amphibians and reptiles of the Anjanaharibe-Sud Massif. Madagascar: elevational distribution and regional endemicity. – Fieldiana Zoology (new series) 90: 79-92.

Raxworthy. C. J. & R. A. Nussbaum (1996b): Amphibians and reptiles of the réserve naturelle intégrale d'Andringitra. Madagascar: a study of elevational distribution and local endemicity. pp. 158-170 in: Goodman. S. M. (ed.): A floral and faunal inventory of the eastern slopes of the réserve naturelle intégrale d'Andringitra. Madagascar: with reference to elevational variation. – Fieldiana Zoology (new series) 85: 158-170.

Raxworthy. C. J.. J. – B. Ramanamanjato & A. Raselimanana (1994): Les reptiles et les amphibiens. – In: Goodman. S. M. & O. Langrand (eds. 1994): Inventaire Biologique Foret de Zombitse. – Recherches pour le developpement Série Sciences biologiques No Spécial 1994. Antananarivo. pp. 41-57.
