# Supplementary material for: Spatial Biodiversity Patterns of Madagascar's Amphibians and Reptiles
Source: PLoS One. 2016 Jan 6;11(1):e0144076. doi: 10.1371/journal.pone.0144076 (PMC4703303; doi:10.1371/journal.pone.0144076)
Supplement: S3 Table — Data extracted from surveys listed in S1 Table. (DOC) [file pone.0144076.s004.doc]

**Spatial biodiversity patterns of Madagascar's amphibians and reptiles**

Jason Brown, Neftali Sillero, Frank Glaw, Parfait Bora, David R. Vieites, Miguel Vences

**Supplementary Materials**

**S3 Table**. Metadata and geographical location of herpetofaunal communities used for analysis. Data extracted from surveys listed in S1 Table.

| **Site** | **wet season 1/dry season 2/both 3** | **pitfall traps yes (1). no (0)** | **number of field techniques** | **Latitude (in red: not from orig. pub.)** | **Longitude (in red: not from orig. pub)** | **duration of survey(days)** | **altitude** | **bioclimatic region 1 humid. 2 subhumid. 3 dry. 4 subarid. 5 montane** | **Humid and subhumid. north 1 /south 2** | **Year of survey** | **period of survey** |
| --- | --- | --- | --- | --- | --- | --- | --- | --- | --- | --- | --- |
| Andohahela s1 | 1 | 1 | 3 | -24.6258 | 46.7653 | 11 | 440 | 1 | 2 | 1995 | 18-28oct 1995 |
| Andohahela s2 | 1 | 1 | 3 | -24.5933 | 46.7383 | 10 | 810 | 1 | 2 | 1995 | 28oct-6nov 1995 |
| Andohahela s3 | 1 | 1 | 3 | -24.5840 | 46.7347 | 10 | 1200 | 1 | 2 | 1995 | 7-16nov 1995 |
| Andohahela s4 | 1 | 1 | 3 | -24.5692 | 46.7308 | 10 | 1500 | 1 | 2 | 1995 | 17-26nov 1995 |
| Andohahela s5 | 1 | 1 | 3 | -24.5117 | 46.7217 | 8 | 1875 | 1 | 2 | 1995 | 27nov-3dec 1995 |
| Andohahela s6 | 1 | 1 | 3 | -24.8167 | 46.6100 | 8 | 120 | 4 |  | 1995 | 7-14dec 1995 |
| Manongarivo s1 | 1 | 0 | 2 | -13.9617 | 48.4333 | 7 | 400 | 2 | 1 | 1998 | 7-13nov 1998 |
| Manongarivo s2 | 1 | 0 | 2 | -13.9767 | 48.4233 | 15 | 785 | 2 | 1 |  | 9-16oct 1998/28feb-6mar 1999 |
| Manongarivo s3 | 1 | 1 | 2 | -14.0000 | 48.4283 | 14 | 1240 | 2 | 1 |  | 19-25oct 1998/8-14mar 1999 |
| Manongarivo s4 | 1 | 1 | 2 | 0.0000 | 0.0000 | 14 | 1600 | 2 | 1 |  | 27oct-3nov 1998/16-22mar 1999 |
| Zombitse s1 | 2 | 1 | 3 | -22.8432 | 44.7116 | 11 | 870 | 4 |  | 1993 | 11-21april 1993 |
| Zombitse s2 | 2 | 1 | 3 | -22.8833 | 44.7167 | 4 | 870 | 4 |  | 1993 | 15-18april 1993 |
| Zombitse s3 | 2 | 1 | 3 | -22.8833 | 44.7167 | 1 | 870 | 4 |  | 1993 | 18-Apr-93 |
| Zombitse s4 | 2 | 1 | 3 | -22.8333 | 44.6000 | 1 | 770 | 4 |  | 1993 | 20-Apr-93 |
| PN Tsimanampetsotsa r1 | na | 1 | 3 | -24.1113 | 43.8390 | 7 |  | 4 |  |  |  |
| PN Andohahela Parcelle2 r2 | na | 1 | 3 | -24.8881 | 46.5900 | 7 |  | 4 |  |  |  |
| Mikea r3 | na | 1 | 3 | -22.5000 | 43.3833 | 7 |  | 4 |  |  |  |
| Anjanaharibe Sud versant ouest s1 | 1 | 1 | 3 | -14.7833 | 49.4417 | 8 | 1200 | 2 | 1 | 1999 | 25oct-2nov 1999 |
| Anjanaharibe Sud versant ouest s2 | 1 | 1 | 3 | -14.5433 | 49.4250 | 8 | 1600 | 2 | 1 | 1999 | 3-10nov 1999 |
| Foret Betaolana site3 | 1 | 1 | 3 | -14.5383 | 49.4383 | 8 | 800 | 1 | 1 | 1999 | 7-14oct 1999 |
| Foret Betaolana site4 | 1 | 1 | 3 | -14.5433 | 49.4250 | 8 | 1200 | 1 | 1 | 1999 | 15-22oct 1999 |
| Marojejy versant nord ouest s5 | 1 | 1 | 3 | -14.4267 | 49.6083 | 19 | 810 | 1 | 1 | 2001 | 13-22oct 2001/5-13feb 2002 |
| Marojejy versant nord ouest s6 | 1 | 1 | 3 | -14.4367 | 49.6200 | 8 | 1175 | 1 | 1 | 2001 | 23-30oct 2001 |
| Vohibasia s1 | 1 | 1 | 1 | -22.4667 | 44.7500 | 6 | 780 | 4 |  | 1996 | 10-15jan 1996 |
| Vohimena s1 | 1 | 1 | 1 | -22.7167 | 44.8833 | 1 | 710 | 4 |  | 1996 | 10-15jan 1996 |
| Tampolo s1 | 2 | 1 | 3 | -17.2833 | 49.4167 | 17 | 10 | 2 | 1 |  | 2-18april xx |
| Anjanaharibe sud e1 | 1 | 1 | 3 | -14.7550 | 49.5050 | 13 | 950 | 1 | 1 | 1994 | 18-30oct 1994 |
| Anjanaharibe sud e2 | 1 | 1 | 3 | -14.7450 | 49.4617 | 12 | 1350 | 1 | 1 | 1994 | 1-12nov 1994 |
| Anjanaharibe sud e3 | 1 | 1 | 3 | -14.7417 | 49.4417 | 10 | 1700 | 1 | 1 | 1994 | 14-23nov 1994 |
| Anjanaharibe sud e4 | 1 | 1 | 3 | -14.7467 | 49.4167 | 5 | 2000 | 1 | 1 | 1994 | 24-28nov 1994 |
| Anjanaharibe sud w1 | 1 | 1 | 3 | -14.7667 | 49.4500 | 10 | 1100 | 1 | 1 | 1996 | 25jan-3feb 1996 |
| Anjanaharibe sud w2 | 1 | 1 | 3 | -14.7667 | 49.4333 | 7 | 1600 | 1 | 1 | 1996 | 5-11feb 1996 |
| HSDED Marojejy s1 t1 450 | 1 | 1 | 3 | -14.4367 | 49.7750 | 14 | 325 | 1 | 1 |  | 16nov 1992/3-5dec 1992/4-13oct 1996 |
| HSDED Marojejy s2 t2 750 | 1 | 1 | 3 | -14.4333 | 49.7617 | 28 | 850 | 1 | 1 |  | 16nov-2dec 1992/14-24oct 1996 |
| HSDED Marojejy s3 t3 1225 | 1 | 1 | 3 | -14.4367 | 49.7417 | 14 | 1350 | 1 | 1 |  | 27-30nov 1992/24oct-3nov 1996 |
| HSDED Marojejy s4 t4 1625 | 1 | 1 | 3 | -14.4400 | 49.7417 | 10 | 1700 | 1 | 1 | 1996 | 4-12/19nov 1996 |
| HSDED Marojejy s5 t5 1875 | 1 | 1 | 3 | -14.4400 | 49.7350 | 8 | 2133 | 1 | 1 |  | 30nov 1992/13-19nov 1996 |
| HSDED Marojejy s 80 | 1 | 0 | 1 | -14.4000 | -49.7000 | 1 | 80 | 1 | 1 |  |  |
| HSDED Marojejy s Mandena 70-100 | 1 | 0 | 1 | -14.4100 | -49.7000 | 1 | 100 | 1 | 1 |  |  |
| HSDED Marojejy s 800 close Andapa | 1 | 0 | 1 | -14.4200 | -49.7000 | 1 | 800 | 1 | 1 |  |  |
| RNI now PN Andringitra s1 c1 720 | 1 | 1 | 2 | -22.2222 | 47.0247 | 9 | 800 | 2 | 2 | 1993 | 14-21nov 1993/17dec 1993 |
| RNI now PN Andringitra s2 c2 810 | 1 | 1 | 2 | -22.2278 | 47.0036 | 8 | 860 | 2 | 2 | 1993 | 22-29nov 1993 |
| RNI now PN Andringitra s3 c3 1210 | 1 | 1 | 2 | -22.2228 | 46.9717 | 8 | 1350 | 2 | 2 | 1993 | 30nov-7dec 1993 |
| RNI now PN Andringitra s c4 1625 | 1 | 1 | 2 | -22.1942 | 46.9711 | 7 | 1700 | 2 | 2 | 1993 | 8-12dec/15-16dec 1993 |
| RNI now PN Andringitra s5 c5 2075 | 1 | 1 | 2 | -22.1694 | 46.9444 | 2 | 2300 | 2 | 2 | 1993 | 13-14dec 1993 |
| RS Pic Ivohibe s1 900 | 1 | 1 | 3 | -22.4833 | 46.9667 | 10 | 900 | 5 |  | 1997 | 6oct-19nov 1997 |
| RS Pic Ivohibe s2 1200 | 1 | 1 | 3 | -22.5000 | 47.0000 | 10 | 1200 | 5 |  | 1997 | 6oct-19nov 1997 |
| RS Pic Ivohibe s3 1575 | 1 | 1 | 3 | -22.5667 | 47.0000 | 10 | 1575 | 5 |  | 1997 | 6oct-19nov 1997 |
| Corridor s1 1200 | 1 | 1 | 3 | -22.5100 | 47.0000 | 10 | 1200 | 2 | 2 | 1997 | 6oct-19nov 1997 |
| Corridor s2 900 | 1 | 1 | 3 | -22.5200 | 47.0000 | 10 | 900 | 2 | 2 | 1997 | 6oct-19nov 1997 |
| Andranomay Anjozorobe s1 | 1 | 1 | 3 | -18.4800 | 47.9550 | 8 | 1300 | 2 | 2 | 1996 | 14-21dec 1996 |
| Befotaka Midongy s1 Rozabe | 1 | 1 | 3 | -23.7366 | 47.0230 | 7 | 850 | 1 | 2 | 2005 | 29sept-5oct 2005 |
| Befotaka Midongy s2 Kilimagnarivo | 1 | 1 | 3 | -23.7975 | 47.0096 | 7 | 890 | 1 | 2 | 2005 | 7-13oct 2005 |
| Tsingy de Bemaraha s1 | 1 | 0 | 2 | -18.6817 | 44.6258 | 6 | 103 | 3 |  | 2006 | 15-17mar 2006/2-4 april 2006 |
| Tsingy de Bemaraha s2 | 1 | 1 | 3 | -18.7086 | 44.7172 | 13 | 146 | 3 |  | 2006 | 13-30jan 2006/17-23mar 2006 |
| Tsingy de Bemaraha s3 | 1 | 1 | 3 | -18.7842 | 44.7794 | 4 | 177 | 3 |  | 2006 | 24-25mar 2006/31mar-1avr 2006 |
| Tsingy de Bemaraha s4 | 1 | 1 | 3 | -18.7972 | 44.8603 | 8 | 427 | 3 |  | 2006 | 26feb-7mar 2006/25-31mar 2006 |
| Tsingy de Bemaraha s5 | 1 | 1 | 3 | -18.6708 | 44.8239 | 10 | 286 | 3 |  | 2006 | 5-14feb 2006 |
| Tsingy de Bemaraha s6 | 1 | 1 | 3 | -18.6619 | 44.8258 | 10 | 403 | 3 |  | 2006 | 15-24feb 2006 |
| Tsingy de Bemaraha s7 | 1 | 1 | 3 | -18.7372 | 44.9247 | 10 | 571 | 3 |  | 2006 | 8-17mar 2006 |
| Tsingy de Bemaraha s8 | 1 | 1 | 3 | -19.0356 | 44.7747 | 11 | 65 | 3 |  | 2006 | 16-26nov 2006 |
| Tsingy de Bemaraha s9 | 1 | 1 | 3 | -19.1311 | 44.8089 | 10 | 57 | 3 |  | 2006 | 6-15 dec 2006 |
| Tsingy de Bemaraha s10 | 1 | 1 | 3 | -19.1403 | 44.8283 | 10 | 59 | 3 |  | 2006 | 27nov-6dec 2006 |
| Nosy Be RNI Lokobe a1 | 1 | 1 | 3 | -13.4167 | 48.3333 | 23 | 210 | 3 |  | 1993 | 0ct 1993 |
| Nosy Be RNI Lokobe a2 | 1 | 1 | 3 | -13.4000 | 48.3000 | 8 | 35 | 3 |  | 1999 | 1-Feb-99 |
| Tsaratanana s1 Andampy | 1 | 0 | 2 | -14.0422 | 48.7617 | 3 | 730 | 2 | 1 | 2001 | 2;10-11feb 2001 |
| Tsaratanana s2 Antsahamanara | 1 | 0 | 2 | -14.0450 | 48.7933 | 9 | 1100 | 2 | 1 | 2001 | 3-11feb 2001 |
| Tsaratanana s3 Camp-Norbert | 1 | 0 | 2 | -13.9480 | 48.4578 | 2 | 288 | 2 | 1 | 2003 | jan-feb 2003 |
| Tsaratanana s4 Camp-0 | 1 | 0 | 2 | -13.9755 | 48.4267 | 2 | 688 | 2 | 1 | 2003 | jan-feb 2003 |
| Tsaratanana s5 Camp-1 | 1 | 0 | 2 | -14.0432 | 48.7612 | 2 | 600 | 2 | 1 | 1997 | 14-15feb 1997 |
| Montagne des Francais / Andavakoera w1 | 3 | 1 | 3 | -12.3297 | 49.3675 | 16 | 140 | 3 |  | 2005 | 7april-15juin 2005/28juin-5sept2005/5oct-14dec 2005/3jan-7mar 2006 |
| Montagne des Francais / Andavakoera d1 | 3 | 1 | 3 | -12.3100 | 49.4000 | 16 | 318 | 3 |  | 2005 | 7april-15juin 2005/28juin-5sept2005/5oct-14dec 2005/3jan-7mar 2006 |
| Montagne des Francais / Andavakoera d2 | 3 | 1 | 3 | -12.3200 | 49.4000 | 16 | 334 | 3 |  | 2005 | 7april-15juin 2005/28juin-5sept2005/5oct-14dec 2005/3jan-7mar 2006 |
| Montagne des Francais / Andavakoera w2 | 3 | 1 | 3 | -12.3300 | 49.4000 | 16 | 100 | 3 |  | 2005 | 7april-15juin 2005/28juin-5sept2005/5oct-14dec 2005/3jan-7mar 2006 |
| Central high plateau s1 Soamazaka | 1 | 1 | 3 | -20.7563 | 47.2940 | 6 | 1650 | 2 | 1 | 2003 | 18-23jan 2003 |
| Central high plateau s2 Vohitsokina | 1 | 1 | 3 | -20.7052 | 47.2873 | 5 | 1620 | 2 | 1 | 2003 | 24-28jan 2003 |
| Central high plateau s3 Farihimazava | 1 | 1 | 3 | -20.8350 | 47.3325 | 5 | 1420 | 2 | 1 | 2003 | 30jan-3feb 2003 |
| Central high plateau s4 Vatolampy | 1 | 1 | 3 | -20.8280 | 47.3190 | 1 | 1580 | 2 | 1 | 2003 | 3-Feb-03 |
| Central high plateau s5 Antratrabe 1 | 1 | 1 | 3 | -19.5175 | 47.8157 | 4 | 1600 | 2 | 1 | 2003 | 9-12feb 2003 |
| Central high plateau s6 Antratrabe 2 | na | 1 | 3 | -19.5000 | 47.8000 | 4 |  | 2 | 1 |  |  |
| Central high plateau s7 Ambatodradama | 1 | 1 | 3 | -19.6475 | 46.0498 | 4 | 925 | 2 | 1 | 2004 | 18-21oct 2004 |
| Central high plateau s8 Itremo | 1 | 1 | 3 | -20.6022 | 46.57139 | 4 |  | 2 | 1 |  |  |
| Central high plateau s9 Andrangoloaka | 1 | 1 | 3 | -19.0333 | 47.9167 | 7 |  | 2 | 1 |  |  |
| Ankarafantsika s1 | na | 1 | 3 | -16.335 | 46.7925 | 7 |  | 3 |  |  |  |
| Ankarafantsika s2 | na | 1 | 3 | -16.66667 | 46.80167 | 7 |  | 3 |  |  |  |
| Ankarafantsika s3 | na | 1 | 3 | -16.66667 | 46.9503 | 7 |  | 3 |  |  |  |
| Corridor Andringitra-Ranomafana s1 Ambatambe | 1 | 1 | 3 | -21.8200 | 47.3550 | 8 | 625 | 1 | 2 | 2000 | 14-21nov 2000 |
| Corridor Andringitra-Ranomafana s2 Ankopakopaka | 1 | 1 | 3 | -21.8233 | 47.3367 | 7 | 645 | 1 | 2 | 2000 | 7-13nov 2000 |
| Corridor Andringitra-Ranomafana s3 Mandriandry | 1 | 1 | 3 | -21.5883 | 47.4850 | 10 | 750 | 1 | 2 | 2000 | 18-27oct 2000 |
| Corridor Andringitra-Ranomafana s4 Ambahaka | 1 | 1 | 3 | -21.7367 | 47.4083 | 8 | 750 | 1 | 2 | 2000 | 28oct-5nov 2000 |
| Corridor Andringitra-Ranomafana s5 Andrambovato | 1 | 1 | 3 | -21.5117 | 47.4100 | 7 | 1075 | 1 | 2 | 2000 | 13-19oct 2000 |
| Corridor Andringitra-Ranomafana s6 Vinanitelo | 1 | 1 | 3 | -21.7767 | 47.3467 | 7 | 1100 | 1 | 2 | 2000 | 23-29oct 2000 |
| Corridor Andringitra-Ranomafana s7 Manambolo 1 | 1 | 1 | 3 | -22.1433 | 47.0217 | 7 | 1300 | 1 | 2 | 1999 | 23-29nov 1999 |
| Corridor Andringitra-Ranomafana s8 Manambolo 2 | 1 | 1 | 3 | -22.1633 | 47.0417 | 6 | 1600 | 1 | 2 | 1999 | 1-6dec 1999 |
| PN Ranomafana s9 | 1 | 1 | 3 | -21.3017 | 47.4567 | 8 | 910 | 1 | 2 | 2000 | 6-13dec 2000 |
| PN Ranomafana s10 | 1 | 1 | 3 | -21.2017 | 47.4567 | 9 | 970 | 1 | 2 | 2000 | 26nov-4dec 2000 |
| PN Ranomafana s11 | 1 | 1 | 3 | -21.2900 | 47.4333 | 7 | 1025 | 1 | 2 | 2000 | 3-9oct 2000 |
| Mikea s1 Ankazomafio | 1 | 1 | 3 | -22.7783 | 43.5233 | 8 | 80 | 4 |  | 2003 | 13-20feb 2003 |
| Mikea s2 Abrahama Jiloriaky | 1 | 1 | 3 | -22.8000 | 43.4333 | 8 | 60 | 4 |  | 2003 | 20-27feb 2003 |
| Mikea s3 Andalandomo | 1 | 1 | 3 | -22.2650 | 43.4783 | 4 | 80 | 4 |  | 2003 | 3-6mar 2003 |
| Mikea s4 Ankindranoky | 1 | 1 | 3 | -22.2167 | 43.3300 | 5 | 50 | 4 |  | 2003 | 9-13mar 2003 |
| Mikea s5 Ankotapiky | 1 | 1 | 3 | -21.8750 | 43.3767 | 6 | 80 | 4 |  | 2003 | 15-20 mar 2003 |
| Mikea s6 Maharihy | 1 | 1 | 3 | -21.8667 | 43.6600 | 5 | 70 | 4 |  | 2003 | 22-26 mar 2003 |
