# Supplementary material for: Spatial Biodiversity Patterns of Madagascar's Amphibians and Reptiles
Source: PLoS One. 2016 Jan 6;11(1):e0144076. doi: 10.1371/journal.pone.0144076 (PMC4703303; doi:10.1371/journal.pone.0144076)
Supplement: S4 Table — Data extracted from surveys listed in S1 Table. (DOC) [file pone.0144076.s005.doc]

**Spatial biodiversity patterns of Madagascar's amphibians and reptiles**

Jason Brown, Neftali Sillero, Frank Glaw, Parfait Bora, David R. Vieites, Miguel Vences

**Supplementary Materials**

**S4 Table.** Species numbers of major amphibian and reptile groups used for comparison of herpetofaunal communities. Data extracted from surveys listed in S1 Table.

| **Site** | **amphiians and reptiles** | **amphibians** | **reptiles** | **proportion reptiles/amphibians** | **proportion amphibians/reptiles** | **Hyperoliidae** | **Microhylids (no Cophylinae)** | **Cophylinae** | **Mantellidae** | **Tortoises** | **Chameleons** | **Furcifer** | **Calumma** | **Brookesia** | **Iguanids** | **Gerrhosaurids** | **Zonosaurus** | **Tracheloptychus** | **Scincidae** | **Gekkonidae** | **Lamprophiidae** | **Typhlopidae / Xenotyphlopidae** |
| --- | --- | --- | --- | --- | --- | --- | --- | --- | --- | --- | --- | --- | --- | --- | --- | --- | --- | --- | --- | --- | --- | --- |
| Andohahela s1 | 38 | 24 | 14 | 0.368 | 0.632 | 0 | 0 | 3 | 21 | 0 | 2 | 0 | 1 | 1 | 0 | 1 | 1 | 0 | 3 | 2 | 6 | 0 |
| Andohahela s2 | 45 | 29 | 16 | 0.356 | 0.644 | 0 | 0 | 5 | 24 | 0 | 5 | 0 | 4 | 1 | 0 | 1 | 1 | 0 | 4 | 3 | 2 | 0 |
| Andohahela s3 | 42 | 24 | 18 | 0.429 | 0.571 | 0 | 0 | 7 | 17 | 0 | 5 | 0 | 4 | 1 | 0 | 1 | 1 | 0 | 3 | 4 | 5 | 0 |
| Andohahela s4 | 23 | 15 | 8 | 0.348 | 0.652 | 0 | 0 | 3 | 12 | 0 | 6 | 0 | 5 | 1 | 0 | 0 | 0 | 0 | 0 | 0 | 2 | 0 |
| Andohahela s5 | 14 | 5 | 9 | 0.643 | 0.357 | 0 | 0 | 2 | 3 | 0 | 5 | 0 | 4 | 1 | 0 | 0 | 0 | 0 | 1 | 2 | 1 | 0 |
| Andohahela s6 | 34 | 4 | 30 | 0.882 | 0.118 | 0 | 2 | 0 | 1 | 0 | 2 | 2 | 0 | 0 | 4 | 2 | 1 | 1 | 7 | 8 | 4 | 2 |
| Manongarivo s1 | 27 | 12 | 15 | 0.556 | 0.444 | 0 | 0 | 2 | 10 | 0 | 3 | 1 | 1 | 1 | 0 | 1 | 1 | 0 | 0 | 8 | 2 | 1 |
| Manongarivo s2 | 28 | 13 | 15 | 0.536 | 0.464 | 0 | 0 | 3 | 10 | 0 | 2 | 0 | 0 | 2 | 0 | 2 | 2 | 0 | 3 | 4 | 3 | 0 |
| Manongarivo s3 | 25 | 15 | 10 | 0.400 | 0.600 | 0 | 0 | 7 | 8 | 0 | 2 | 0 | 1 | 1 | 0 | 0 | 0 | 0 | 3 | 3 | 2 | 0 |
| Manongarivo s4 | 17 | 8 | 9 | 0.529 | 0.471 | 0 | 0 | 1 | 7 | 0 | 4 | 0 | 3 | 1 | 0 | 0 | 0 | 0 | 3 | 1 | 1 | 0 |
| Zombitse s1 | 21 | 0 | 21 | 1.000 | 0.000 | 0 | 0 | 0 | 0 | 0 | 2 | 2 | 0 | 0 | 1 | 0 | 0 | 0 | 5 | 7 | 5 | 1 |
| Zombitse s2 | 15 | 1 | 14 | 0.933 | 0.067 | 0 | 0 | 0 | 1 | 0 | 2 | 2 | 0 | 0 | 1 | 0 | 0 | 0 | 3 | 5 | 3 | 0 |
| Zombitse s3 | 7 | 0 | 7 | 1.000 | 0.000 | 0 | 0 | 0 | 0 | 0 | 0 | 0 | 0 | 0 | 0 | 0 | 0 | 0 | 2 | 5 | 0 | 0 |
| Zombitse s4 | 15 | 3 | 12 | 0.800 | 0.200 | 0 | 0 | 0 | 2 | 1 | 0 | 0 | 0 | 0 | 1 | 0 | 0 | 0 | 2 | 3 | 4 | 0 |
| PN Tsimanampetsotsa r1 | 42 | 3 | 39 | 0.929 | 0.071 | 0 | 1 | 0 | 1 | 2 | 3 | 3 | 0 | 0 | 3 | 4 | 3 | 1 | 8 | 11 | 6 | 2 |
| PN Andohahela Parcelle2 r2 | 35 | 4 | 31 | 0.886 | 0.114 | 0 | 2 | 0 | 1 | 0 | 2 | 2 | 0 | 0 | 4 | 2 | 1 | 1 | 7 | 8 | 5 | 2 |
| Mikea r3 | 50 | 6 | 44 | 0.880 | 0.120 | 1 | 2 | 0 | 2 | 1 | 3 | 3 | 0 | 0 | 2 | 3 | 2 | 1 | 7 | 8 | 17 | 2 |
| Anjanaharibe Sud versant ouest s1 | 54 | 32 | 22 | 0.407 | 0.593 | 0 | 0 | 7 | 25 | 0 | 6 | 0 | 4 | 2 | 0 | 1 | 1 | 0 | 4 | 5 | 4 | 1 |
| Anjanaharibe Sud versant ouest s2 | 26 | 16 | 10 | 0.385 | 0.615 | 0 | 0 | 9 | 7 | 0 | 6 | 0 | 5 | 1 | 0 | 0 | 0 | 0 | 1 | 2 | 1 | 0 |
| Foret Betaolana site3 | 22 | 10 | 12 | 0.545 | 0.455 | 0 | 0 | 5 | 5 | 0 | 4 | 0 | 3 | 1 | 0 | 1 | 1 | 0 | 2 | 3 | 2 | 0 |
| Foret Betaolana site4 | 31 | 21 | 10 | 0.323 | 0.677 | 0 | 0 | 10 | 11 | 0 | 3 | 0 | 1 | 2 | 0 | 0 | 0 | 0 | 1 | 3 | 3 | 0 |
| Marojejy versant nord ouest s5 | 68 | 36 | 32 | 0.471 | 0.529 | 0 | 0 | 11 | 25 | 0 | 5 | 0 | 2 | 3 | 0 | 3 | 3 | 0 | 7 | 8 | 9 | 0 |
| Marojejy versant nord ouest s6 | 50 | 30 | 20 | 0.400 | 0.600 | 0 | 0 | 12 | 18 | 0 | 7 | 0 | 3 | 4 | 0 | 1 | 1 | 0 | 2 | 5 | 4 | 0 |
| Vohibasia s1 | 26 | 5 | 21 | 0.808 | 0.192 | 1 | 3 | 0 | 1 | 1 | 3 | 2 | 0 | 1 | 2 | 0 | 0 | 0 | 3 | 6 | 5 | 0 |
| Vohimena s1 | 1 | 0 | 1 | 1.000 | 0.000 | 0 | 0 | 0 | 0 | 0 | 0 | 0 | 0 | 0 | 0 | 0 | 0 | 0 | 1 | 0 | 0 | 0 |
| Tampolo s1 | 47 | 16 | 31 | 0.660 | 0.340 | 1 | 0 | 5 | 9 | 0 | 3 | 0 | 1 | 2 | 0 | 2 | 2 | 0 | 4 | 9 | 10 | 1 |
| Anjanaharibe sud e1 | 40 | 20 | 20 | 0.500 | 0.500 | 0 | 0 | 5 | 14 | 0 | 4 | 1 | 2 | 1 | 0 | 1 | 1 | 0 | 3 | 5 | 6 | 0 |
| Anjanaharibe sud e2 | 45 | 31 | 14 | 0.311 | 0.689 | 0 | 0 | 8 | 23 | 0 | 5 | 0 | 5 | 0 | 0 | 1 | 1 | 0 | 2 | 4 | 1 | 0 |
| Anjanaharibe sud e3 | 24 | 18 | 6 | 0.250 | 0.750 | 0 | 0 | 8 | 10 | 0 | 4 | 0 | 4 | 0 | 0 | 0 | 0 | 0 | 1 | 0 | 1 | 0 |
| Anjanaharibe sud e4 | 10 | 8 | 2 | 0.200 | 0.800 | 0 | 0 | 4 | 4 | 0 | 1 | 0 | 1 | 0 | 0 | 0 | 0 | 0 | 0 | 0 | 1 | 0 |
| Anjanaharibe sud w1 | 50 | 34 | 16 | 0.320 | 0.680 | 0 | 0 | 7 | 27 | 0 | 5 | 0 | 4 | 1 | 0 | 1 | 1 | 0 | 5 | 1 | 3 | 1 |
| Anjanaharibe sud w2 | 38 | 21 | 17 | 0.447 | 0.553 | 0 | 0 | 10 | 11 | 0 | 7 | 0 | 4 | 3 | 0 | 0 | 0 | 0 | 5 | 2 | 3 | 0 |
| HSDED Marojejy s1 t1 450 | 73 | 35 | 38 | 0.521 | 0.479 | 0 | 0 | 6 | 29 | 0 | 7 | 1 | 3 | 3 | 0 | 3 | 3 | 0 | 6 | 12 | 8 | 1 |
| HSDED Marojejy s2 t2 750 | 76 | 36 | 40 | 0.526 | 0.474 | 0 | 0 | 8 | 28 | 0 | 8 | 1 | 3 | 4 | 0 | 3 | 3 | 0 | 8 | 9 | 9 | 2 |
| HSDED Marojejy s3 t3 1225 | 37 | 23 | 14 | 0.378 | 0.622 | 0 | 0 | 9 | 14 | 0 | 7 | 0 | 4 | 3 | 0 | 0 | 0 | 0 | 3 | 3 | 1 | 0 |
| HSDED Marojejy s4 t4 1625 | 18 | 10 | 8 | 0.444 | 0.556 | 0 | 0 | 2 | 8 | 0 | 5 | 0 | 3 | 2 | 0 | 0 | 0 | 0 | 0 | 1 | 2 | 0 |
| HSDED Marojejy s5 t5 1875 | 12 | 6 | 6 | 0.500 | 0.500 | 0 | 0 | 2 | 4 | 0 | 2 | 0 | 2 | 0 | 0 | 0 | 0 | 0 | 1 | 2 | 1 | 0 |
| HSDED Marojejy s 80 | 1 | 1 | 0 | 0.000 | 1.000 | 0 | 0 | 0 | 0 | 0 | 0 | 0 | 0 | 0 | 0 | 0 | 0 | 0 | 0 | 0 | 0 | 0 |
| HSDED Marojejy s Mandena 70-100 | 8 | 2 | 6 | 0.750 | 0.250 | 0 | 0 | 0 | 1 | 0 | 0 | 0 | 0 | 0 | 0 | 0 | 0 | 0 | 2 | 1 | 3 | 0 |
| HSDED Marojejy s 800 close Andapa | 1 | 0 | 1 | 1.000 | 0.000 | 0 | 0 | 0 | 0 | 0 | 0 | 0 | 0 | 0 | 0 | 0 | 0 | 0 | 0 | 0 | 1 | 0 |
| RNI now PN Andringitra s1 c1 720 | 44 | 27 | 17 | 0.386 | 0.614 | 1 | 0 | 3 | 22 | 0 | 3 | 0 | 1 | 2 | 0 | 1 | 1 | 0 | 4 | 2 | 7 | 0 |
| RNI now PN Andringitra s2 c2 810 | 50 | 36 | 14 | 0.280 | 0.720 | 0 | 1 | 5 | 30 | 0 | 2 | 0 | 1 | 1 | 0 | 1 | 1 | 0 | 5 | 3 | 3 | 0 |
| RNI now PN Andringitra s3 c3 1210 | 37 | 26 | 11 | 0.297 | 0.703 | 0 | 0 | 6 | 20 | 0 | 5 | 0 | 4 | 1 | 0 | 0 | 0 | 0 | 2 | 1 | 3 | 0 |
| RNI now PN Andringitra s c4 1625 | 12 | 8 | 4 | 0.333 | 0.667 | 0 | 0 | 3 | 5 | 0 | 3 | 0 | 2 | 1 | 0 | 0 | 0 | 0 | 0 | 0 | 1 | 0 |
| RNI now PN Andringitra s5 c5 2075 | 10 | 5 | 5 | 0.500 | 0.500 | 0 | 0 | 1 | 4 | 0 | 2 | 1 | 1 | 0 | 0 | 0 | 0 | 0 | 1 | 2 | 0 | 0 |
| RS Pic Ivohibe s1 900 | 33 | 20 | 13 | 0.394 | 0.606 | 0 | 0 | 2 | 18 | 0 | 5 | 0 | 4 | 1 | 0 | 0 | 0 | 0 | 5 | 2 | 1 | 0 |
| RS Pic Ivohibe s2 1200 | 35 | 17 | 18 | 0.514 | 0.486 | 0 | 0 | 6 | 11 | 0 | 7 | 0 | 5 | 2 | 0 | 0 | 0 | 0 | 7 | 3 | 1 | 0 |
| RS Pic Ivohibe s3 1575 | 21 | 9 | 12 | 0.571 | 0.429 | 0 | 0 | 2 | 7 | 0 | 4 | 0 | 4 | 0 | 0 | 1 | 1 | 0 | 4 | 1 | 2 | 0 |
| Corridor s1 1200 | 34 | 18 | 16 | 0.471 | 0.529 | 0 | 0 | 4 | 14 | 0 | 6 | 0 | 4 | 2 | 0 | 1 | 1 | 0 | 5 | 2 | 2 | 0 |
| Corridor s2 900 | 38 | 21 | 17 | 0.447 | 0.553 | 0 | 0 | 4 | 17 | 0 | 5 | 0 | 3 | 2 | 0 | 0 | 0 | 0 | 5 | 4 | 2 | 0 |
| Andranomay Anjozorobe s1 | 47 | 24 | 23 | 0.489 | 0.511 | 1 | 0 | 3 | 20 | 0 | 8 | 2 | 4 | 2 | 0 | 2 | 2 | 0 | 3 | 5 | 3 | 1 |
| Befotaka Midongy s1 Rozabe | 30 | 18 | 12 | 0.400 | 0.600 | 0 | 0 | 2 | 15 | 0 | 1 | 0 | 0 | 1 | 0 | 1 | 1 | 0 | 2 | 2 | 5 | 0 |
| Befotaka Midongy s2 Kilimagnarivo | 36 | 27 | 9 | 0.250 | 0.750 | 1 | 0 | 4 | 21 | 0 | 1 | 0 | 0 | 1 | 0 | 2 | 2 | 0 | 3 | 1 | 2 | 0 |
| Tsingy de Bemaraha s1 | 11 | 2 | 9 | 0.818 | 0.182 | 0 | 0 | 0 | 2 | 0 | 2 | 2 | 0 | 0 | 2 | 0 | 0 | 0 | 1 | 4 | 0 | 0 |
| Tsingy de Bemaraha s2 | 43 | 14 | 29 | 0.674 | 0.326 | 2 | 1 | 1 | 9 | 0 | 5 | 2 | 0 | 3 | 1 | 2 | 2 | 0 | 4 | 10 | 6 | 0 |
| Tsingy de Bemaraha s3 | 15 | 5 | 10 | 0.667 | 0.333 | 0 | 0 | 0 | 4 | 0 | 0 | 0 | 0 | 0 | 1 | 0 | 0 | 0 | 1 | 7 | 1 | 0 |
| Tsingy de Bemaraha s4 | 36 | 13 | 23 | 0.639 | 0.361 | 0 | 2 | 2 | 8 | 0 | 6 | 3 | 0 | 3 | 0 | 1 | 1 | 0 | 1 | 7 | 5 | 1 |
| Tsingy de Bemaraha s5 | 13 | 3 | 10 | 0.769 | 0.231 | 0 | 0 | 0 | 3 | 0 | 4 | 1 | 0 | 3 | 0 | 1 | 1 | 0 | 1 | 3 | 1 | 0 |
| Tsingy de Bemaraha s6 | 23 | 6 | 17 | 0.739 | 0.261 | 0 | 2 | 0 | 4 | 0 | 4 | 1 | 0 | 3 | 0 | 1 | 1 | 0 | 6 | 4 | 1 | 1 |
| Tsingy de Bemaraha s7 | 10 | 3 | 7 | 0.700 | 0.300 | 0 | 1 | 0 | 2 | 0 | 4 | 2 | 0 | 2 | 0 | 0 | 0 | 0 | 0 | 1 | 2 | 0 |
| Tsingy de Bemaraha s8 | 27 | 6 | 21 | 0.778 | 0.222 | 0 | 0 | 2 | 4 | 0 | 3 | 1 | 0 | 2 | 0 | 1 | 1 | 0 | 5 | 7 | 4 | 1 |
| Tsingy de Bemaraha s9 | 37 | 8 | 29 | 0.784 | 0.216 | 0 | 2 | 1 | 5 | 0 | 5 | 3 | 0 | 2 | 1 | 2 | 2 | 0 | 6 | 9 | 5 | 1 |
| Tsingy de Bemaraha s10 | 13 | 4 | 9 | 0.692 | 0.308 | 0 | 1 | 1 | 2 | 0 | 2 | 2 | 0 | 0 | 1 | 1 | 1 | 0 | 3 | 1 | 1 | 0 |
| Nosy Be RNI Lokobe a1 | 56 | 12 | 44 | 0.786 | 0.214 | 0 | 0 | 5 | 6 | 0 | 6 | 1 | 2 | 3 | 0 | 4 | 4 | 0 | 5 | 14 | 11 | 2 |
| Nosy Be RNI Lokobe a2 | 31 | 8 | 23 | 0.742 | 0.258 | 0 | 0 | 3 | 5 | 0 | 4 | 1 | 2 | 1 | 0 | 3 | 3 | 0 | 2 | 10 | 4 | 0 |
| Tsaratanana s1 Andampy | 11 | 7 | 4 | 0.364 | 0.636 | 0 | 0 | 2 | 5 | 0 | 2 | 1 | 1 | 0 | 0 | 0 | 0 | 0 | 0 | 1 | 1 | 0 |
| Tsaratanana s2 Antsahamanara | 35 | 21 | 14 | 0.400 | 0.600 | 0 | 0 | 8 | 13 | 0 | 5 | 0 | 4 | 1 | 0 | 0 | 0 | 0 | 1 | 3 | 2 | 1 |
| Tsaratanana s3 Camp-Norbert | 16 | 9 | 7 | 0.438 | 0.563 | 0 | 0 | 1 | 8 | 0 | 2 | 0 | 0 | 2 | 0 | 0 | 0 | 0 | 0 | 3 | 1 | 0 |
| Tsaratanana s4 Camp-0 | 23 | 15 | 8 | 0.348 | 0.652 | 0 | 0 | 2 | 13 | 0 | 0 | 0 | 0 | 0 | 0 | 1 | 1 | 0 | 0 | 5 | 1 | 0 |
| Tsaratanana s5 Camp-1 | 23 | 16 | 7 | 0.304 | 0.696 | 0 | 0 | 3 | 13 | 0 | 2 | 0 | 1 | 1 | 0 | 0 | 0 | 0 | 0 | 2 | 2 | 1 |
| Montagne des Francais / Andavakoera w1 | 43 | 8 | 35 | 0.814 | 0.186 | 0 | 0 | 2 | 5 | 1 | 3 | 2 | 0 | 1 | 0 | 1 | 1 | 0 | 4 | 9 | 13 | 1 |
| Montagne des Francais / Andavakoera d1 | 36 | 5 | 31 | 0.861 | 0.139 | 0 | 0 | 1 | 3 | 0 | 4 | 3 | 0 | 1 | 0 | 2 | 2 | 0 | 2 | 9 | 10 | 1 |
| Montagne des Francais / Andavakoera d2 | 40 | 6 | 34 | 0.850 | 0.150 | 0 | 0 | 1 | 4 | 0 | 3 | 3 | 0 | 0 | 0 | 2 | 2 | 0 | 2 | 11 | 14 | 1 |
| Montagne des Francais / Andavakoera w2 | 52 | 8 | 44 | 0.846 | 0.154 | 0 | 0 | 2 | 5 | 0 | 6 | 3 | 0 | 3 | 0 | 2 | 2 | 0 | 5 | 11 | 16 | 1 |
| Central high plateau s1 Soamazaka | 21 | 12 | 9 | 0.429 | 0.571 | 0 | 0 | 0 | 11 | 0 | 2 | 2 | 0 | 0 | 1 | 1 | 1 | 0 | 1 | 1 | 2 | 0 |
| Central high plateau s2 Vohitsokina | 13 | 7 | 6 | 0.462 | 0.538 | 0 | 0 | 0 | 6 | 0 | 2 | 2 | 0 | 0 | 1 | 0 | 0 | 0 | 1 | 0 | 2 | 0 |
| Central high plateau s3 Farihimazava | 13 | 8 | 5 | 0.385 | 0.615 | 0 | 0 | 1 | 6 | 0 | 3 | 3 | 0 | 0 | 0 | 0 | 0 | 0 | 1 | 0 | 1 | 0 |
| Central high plateau s4 Vatolampy | 15 | 10 | 5 | 0.333 | 0.667 | 1 | 0 | 2 | 7 | 0 | 4 | 0 | 4 | 0 | 0 | 0 | 0 | 0 | 1 | 0 | 0 | 0 |
| Central high plateau s5 Antratrabe 1 | 16 | 12 | 4 | 0.250 | 0.750 | 2 | 0 | 0 | 9 | 0 | 2 | 2 | 0 | 0 | 0 | 1 | 1 | 0 | 0 | 0 | 1 | 0 |
| Central high plateau s6 Antratrabe 2 | 6 | 4 | 2 | 0.333 | 0.667 | 0 | 0 | 0 | 4 | 0 | 2 | 0 | 2 | 0 | 0 | 0 | 0 | 0 | 0 | 0 | 0 | 0 |
| Central high plateau s7 Ambatodradama | 14 | 9 | 5 | 0.357 | 0.643 | 0 | 0 | 0 | 8 | 0 | 0 | 0 | 0 | 0 | 0 | 0 | 0 | 0 | 1 | 0 | 3 | 0 |
| Central high plateau s8 Itremo | 13 | 10 | 3 | 0.231 | 0.769 | 1 | 0 | 0 | 8 | 0 | 2 | 1 | 1 | 0 | 1 | 0 | 0 | 0 | 0 | 0 | 0 | 0 |
| Central high plateau s9 Andrangoloaka | 17 | 9 | 8 | 0.471 | 0.529 | 0 | 0 | 1 | 8 | 0 | 5 | 3 | 2 | 0 | 0 | 1 | 1 | 0 | 1 | 0 | 1 | 0 |
| Ankarafantsika s1 | 33 | 7 | 26 | 0.788 | 0.212 | 0 | 2 | 1 | 3 | 1 | 4 | 3 | 0 | 1 | 1 | 1 | 1 | 0 | 2 | 6 | 7 | 2 |
| Ankarafantsika s2 | 44 | 9 | 35 | 0.795 | 0.205 | 1 | 1 | 1 | 5 | 0 | 3 | 2 | 0 | 1 | 2 | 1 | 1 | 0 | 3 | 12 | 10 | 1 |
| Ankarafantsika s3 | 42 | 9 | 33 | 0.786 | 0.214 | 1 | 1 | 1 | 5 | 1 | 3 | 2 | 0 | 1 | 2 | 1 | 1 | 0 | 3 | 12 | 8 | 0 |
| Corridor Andringitra-Ranomafana s1 Ambatambe | 26 | 18 | 8 | 0.308 | 0.692 | 2 | 0 | 3 | 13 | 0 | 0 | 0 | 0 | 0 | 0 | 2 | 2 | 0 | 0 | 1 | 4 | 1 |
| Corridor Andringitra-Ranomafana s2 Ankopakopaka | 24 | 20 | 4 | 0.167 | 0.833 | 0 | 1 | 4 | 15 | 0 | 0 | 0 | 0 | 0 | 0 | 0 | 0 | 0 | 2 | 0 | 0 | 0 |
| Corridor Andringitra-Ranomafana s3 Mandriandry | 26 | 21 | 5 | 0.192 | 0.808 | 0 | 0 | 4 | 17 | 0 | 2 | 0 | 2 | 0 | 0 | 0 | 0 | 0 | 2 | 3 | 0 | 0 |
| Corridor Andringitra-Ranomafana s4 Ambahaka | 44 | 30 | 14 | 0.318 | 0.682 | 0 | 0 | 5 | 25 | 0 | 6 | 0 | 5 | 1 | 0 | 0 | 0 | 0 | 3 | 1 | 2 | 0 |
| Corridor Andringitra-Ranomafana s5 Andrambovato | 39 | 27 | 12 | 0.308 | 0.692 | 0 | 0 | 4 | 23 | 0 | 5 | 0 | 4 | 1 | 0 | 1 | 1 | 0 | 2 | 3 | 3 | 0 |
| Corridor Andringitra-Ranomafana s6 Vinanitelo | 47 | 33 | 14 | 0.298 | 0.702 | 0 | 0 | 4 | 28 | 0 | 6 | 0 | 4 | 2 | 0 | 1 | 1 | 0 | 3 | 1 | 3 | 0 |
| Corridor Andringitra-Ranomafana s7 Manambolo 1 | 31 | 19 | 12 | 0.387 | 0.613 | 1 | 0 | 1 | 16 | 0 | 5 | 0 | 4 | 1 | 0 | 2 | 2 | 0 | 3 | 1 | 1 | 0 |
| Corridor Andringitra-Ranomafana s8 Manambolo 2 | 28 | 20 | 8 | 0.286 | 0.714 | 0 | 0 | 5 | 15 | 0 | 6 | 0 | 5 | 1 | 0 | 0 | 0 | 0 | 0 | 0 | 2 | 0 |
| PN Ranomafana s9 | 21 | 16 | 5 | 0.238 | 0.762 | 0 | 0 | 6 | 10 | 0 | 1 | 0 | 1 | 0 | 0 | 0 | 0 | 0 | 1 | 0 | 3 | 0 |
| PN Ranomafana s10 | 37 | 27 | 10 | 0.270 | 0.730 | 0 | 2 | 3 | 21 | 0 | 3 | 0 | 2 | 1 | 0 | 1 | 1 | 0 | 2 | 2 | 2 | 0 |
| PN Ranomafana s11 | 47 | 31 | 16 | 0.340 | 0.660 | 0 | 0 | 6 | 25 | 0 | 5 | 0 | 4 | 1 | 0 | 0 | 0 | 0 | 4 | 3 | 3 | 0 |
| Mikea s1 Ankazomafio | 26 | 0 | 26 | 1.000 | 0.000 | 0 | 0 | 0 | 0 | 1 | 2 | 2 | 0 | 0 | 2 | 3 | 2 | 1 | 4 | 6 | 7 | 1 |
| Mikea s2 Abrahama Jiloriaky | 29 | 1 | 28 | 0.966 | 0.034 | 0 | 1 | 0 | 0 | 1 | 2 | 2 | 0 | 0 | 2 | 3 | 2 | 1 | 6 | 5 | 7 | 1 |
| Mikea s3 Andalandomo | 25 | 0 | 25 | 1.000 | 0.000 | 0 | 0 | 0 | 0 | 0 | 2 | 2 | 0 | 0 | 2 | 3 | 2 | 1 | 3 | 5 | 8 | 1 |
| Mikea s4 Ankindranoky | 27 | 2 | 25 | 0.926 | 0.074 | 0 | 1 | 0 | 1 | 1 | 2 | 2 | 0 | 0 | 2 | 3 | 2 | 1 | 5 | 4 | 6 | 2 |
| Mikea s5 Ankotapiky | 31 | 1 | 30 | 0.968 | 0.032 | 0 | 0 | 0 | 1 | 1 | 2 | 2 | 0 | 0 | 2 | 3 | 2 | 1 | 5 | 4 | 11 | 1 |
| Mikea s6 Maharihy | 24 | 6 | 18 | 0.750 | 0.250 | 1 | 2 | 0 | 2 | 1 | 1 | 1 | 0 | 0 | 2 | 2 | 1 | 1 | 3 | 4 | 3 | 1 |
